# A Modular and Convergent “Stick and Click” Conjugation Platform Enables Fast Antibody Conjugate Library Synthesis

**DOI:** 10.1021/acs.bioconjchem.5c00461

**Published:** 2026-01-07

**Authors:** Connor Livingstone, Simon Nicolle, Gavin Jones, Craig Jamieson

**Affiliations:** † GSK Medicines Research Centre, Gunnels Wood Road, Stevenage, U.K., SG1 2NY; ‡ Department of Pure and Applied Chemistry, University of Strathclyde, 295 Cathedral Street, Glasgow, U.K., G1 1XL

## Abstract

The preparation of antibody drug conjugates (ADC) most often relies on a linear sequence to elaborate the small molecule component, followed by a final bioconjugation step to attach it to its immunoglobulin partner. This linear and iterative approach is incompatible with expedient parallel synthesis and process automation. Here, we describe the design and implementation of a general modular platform for the assembly of ADCs that enables facile variation in the nature of the payload, the linker composition, and the type of bioconjugation technique used. A library of antibody conjugates bringing together several different antibodies and payloads was prepared in a convergent fashion using a range of conjugation methods, as well as cleavable or noncleavable linker technology. Aside from offering a direct comparison of different conjugation method performances, this approach enables a more targeted optimization strategy of conjugate properties by deconvoluting bioconjugation and payload attachment.

## Graphical abstract

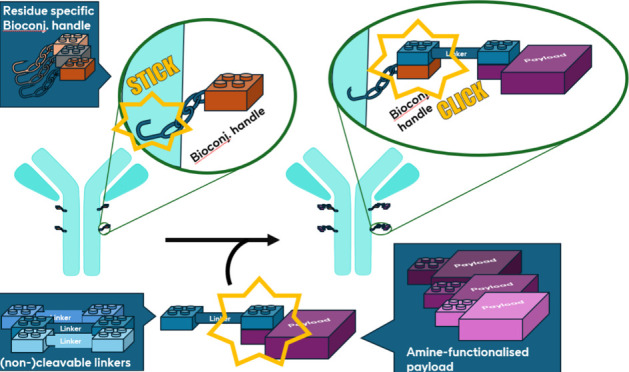

## Introduction

The emergence of antibody–drug conjugates (ADCs) sees these precision medicines playing a growing role in the therapeutic management of hematological tumors and solid tumors.^1^ A therapeutic ADC is typically composed of a monoclonal antibody (mAb) to which small molecule assets are covalently attached through a bioconjugation step. Synthesis of the small molecule component of ADCs is generally carried out in a linear fashion prior to conjugation with the mAb to generate the target ADC (Scheme 1).^2^ Taking into account that all components of the small molecule conjugate (i.e., conjugation method leading,^3^ linker,^4^ payload^5^) can have a significant impact on the biophysical properties of the final conjugate, and that optimization work often requires an iterative approach to identify the best combination, a linear strategy requires *de novo* synthesis and longer iteration times. A further limitation of this approach lies in the fact that the bioconjugation handles used in the small molecule component are usually reactive entities that commonly can only be carried through a limited number of synthesis steps executed under carefully selected experimental conditions. Conversely, it is noteworthy that despite the diversity in bioconjugation strategies targeting specific residues (e.g., targeting tryptophan, tyrosine, methionine, arginine, etc.) reported in the literature,^6–8^ marketed therapeutic ADCs are typically functionalized through lysine and cysteine-conjugation methods.^9^ We reasoned that an approach that segregates the bioconjugation method and the ligand-payload combination would bring better convergence and modularity to the process, as well as allowing a wider variety of biofunctionalization methods to be evaluated for a given target conjugate.

**Scheme 1:**
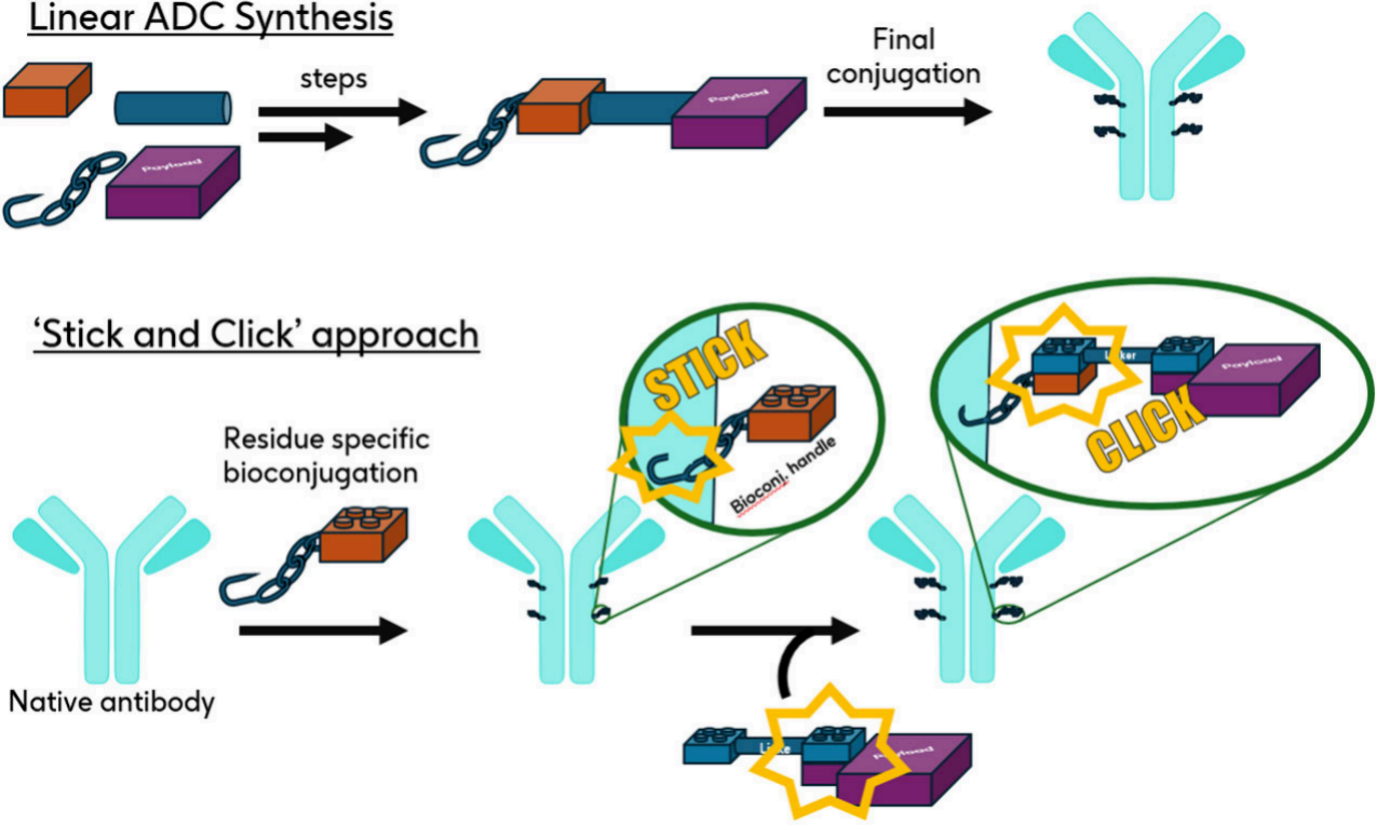
Linear and Convergent “Stick and Click” Approach to ADC Synthesis

In our process design (“Stick and Click” Scheme 1), the target antibody is initially functionalized by a bio-orthogonal reactive handle (“Stick” step) able to react with a linker-payload fragment functionalized with the complementary reactive handle to assemble the full ADC (“Click” step). Among the multiple “click” chemistry methods developed, we selected the widely used bicyclononyne (BCN)-azide strain-promoted azidealkyne cycloaddition (SPAAC) as the preferred bio-orthogonal chemistry approach due to its metal-free implementation, minimal molecular footprint, and good reagent reactivity/stability balance.^10–12^ To enable the expedient variation of payload, we ensured that the linker-payload portion could be assembled in one simple step and telescoped to the final click assembly by using a carbamate moiety derived from *p*-nitrophenyl (PNP) carbonate precursors,^13^ which are therefore applicable to amine-functionalized payloads. Prior examples of site selective post-translational modifications of native proteins introducing a click handle for further modification have been described,^14–19^ but to the best of our knowledge, and despite some notable examples showcasing a late-stage click approach to assemble ADCs,^11,20–24^ no attempt at generalizing this method to demonstrate full modularity of each ADC constitutive element has yet been presented.

In the current study, we report the conceptualization and realization of a modular platform for the construction of ADCs whereby each element (payload/linker/conjugation handle) can be easily modified. Such an approach allowed for rapid data generation to directly establish a comparison between several residue-targeting bioconjugation strategies.

## Results and Discussion

To demonstrate the concept of a modular platform which could readily enable the integration of a range of bioconjugation methods, we selected several approaches including site-selective methods that are less prominent in the field of ADC synthesis (Figure 1). First, a methionine-targeting azide-containing bioconjugation reagent **1** was prepared based on previous work by Toste et al.^25,26^ Methionine targeting is an emerging strategy and exploits a redox approach using finely tuned oxaziridine with the potential to achieve controlled drug-antibody ratio (DAR) profiles due to the low surface expression of lipophilic methionine species.

**Figure 1:**
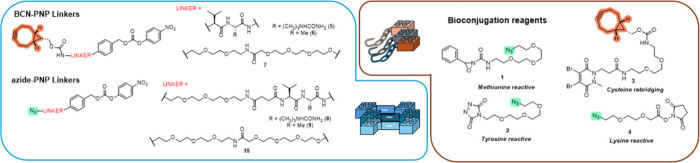
Reagents and activated linkers used in this work.

Second, we sought an efficient tyrosine-targeting reagent. A number of diazo derivatives have been described to enable selective tyrosine functionalization.^27–30^ While application to full length mAb derivatives is not frequently reported, preliminary results suggested the possibility to achieve a controlled DAR profile,^28,31^ presumably due to the lower surface expression of tyrosine compared with more hydrophilic residues, such as lysine. We evaluated several azide-contained reagents based on triazolediones (TADs)^28,32^ or *N*-methylphthalazinedione^29^ and identified reagent **2** ^33^ as the most robust in our hands in terms of stability and desired bioconjugation profile (see SI Section 5b,c).

Additionally, a cysteine rebridging reagent **3** ^34^ was selected. Several methods that enable cysteine conjugation with dual-electrophile reagents have been reported.^22,34–38^ The cysteine rebridging approach presents the advantage of achieving a controlled DAR and therefore more homogeneous conjugates, as well as improving the stability of the resulting ADCs. Here a BCN-functionalized rebridging reagent was chosen due to the incompatibility of an azide moiety with the phosphine-based reductant (TCEP) used during bioconjugation.

Finally, a simple *N*-hydroxysuccinimide reactive lysine reagent **4** ^39,40^ was also prepared to provide a comparison with this established bioconjugation method.

We then turned our attention to the design of the reactive linkers. This component is a crucial element of the current study as it must combine both orthogonal handles to enable payload attachment on one hand and undergo a click process with the prefunctionalized mAb on the other. Additionally, the nature of the linker influences the stability of the ADC *in vivo* and importantly also controls the release of the payload within the target cell/tissue.^41,42^ In the present design, noncleavable linkers (**7** and **10**) were included, as well as cathepsin-sensitive protease-cleavable valine-citrulline and valine-alanine linkers in association with a self-immolative *p*-aminobenzylcarbamate (PABC) spacer to enable traceless payload release (**5**, **6**, **8**, and **9**).^43^ Linkers **5**–**7** bearing a BCN SPAAC-handle are appropriate for any bioconjugation conditions tolerant to the presence of an azide handle such as methionine and tyrosine conjugation with reagents **1** and **2**, respectively. In contrast, the cysteine rebridging bioconjugation employs a TCEP reduction step incompatible with azides; therefore, linkers **8**–**10** incorporating a strained alkyne instead of an azide click component were prepared and used in conjunction with reagent **3**.

### Preliminary Scoping of Residue Specific Conjugation Methods

With all reagents in hand, each bioconjugation method was first assessed, and conditions delivering a similar average DAR (aDAR) were established. For this we employed systems representative of therapeutic antibodies: an anti-HER2 monoclonal IgG1 based on Trastuzumab (denoted **αHER2**), an anti-IL4 IgG1 antibody based on Pascolizumab (denoted **αIL4**), and a proprietary heterodimeric antibody possessing the same Fab regions as the anti-HER2 mAb (denoted **Het**). Trastuzumab has been applied as both a therapeutic antibody (Herceptin)^44^ and as an ADC (Kadcyla and Enhertu), while Pascolizumab was developed as a treatment for asthma.^45^ A summary of the average DAR (aDAR) and DAR analysis obtained with these three antibodies after initial method optimization is shown on Figure 2.

**Figure 2:**
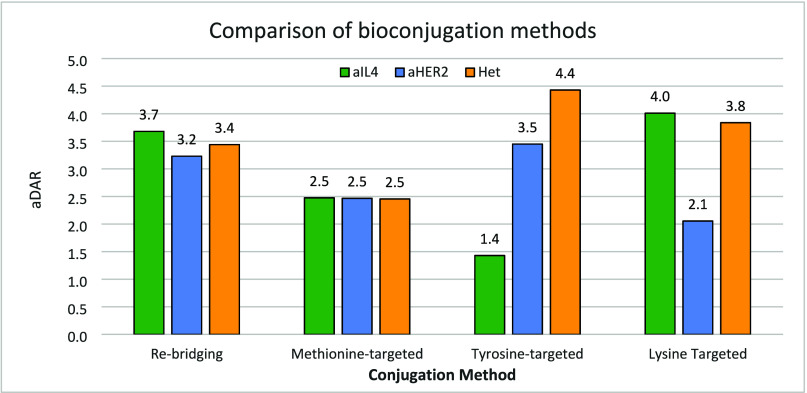
aDAR comparison of three antibodies using each conjugation methods. Rebridging: 20 equiv **3**, 20 equiv TCEP, 18 h, 4 °C, pH 8.5 BBS; methionine conjugation: 7–10 equiv **1**, 18–20 h, 4 °C, pH 7.2 PBS; tyrosine conjugation: 50 equiv **2**, 9% organic solvent, 72 h, 4 °C, pH 7.2 PBS; lysine conjugation: 10 equiv **4**, 18 h, 4 °C, pH 7.2 PBS.

The disulfide rebridging method using reagent **3** gave satisfactory levels of conjugation across the three antibodies studied after optimization of the initial reduction conditions. The associated aDAR was in a narrow range [3.2–3.7] across the antibody set, as ascertained by mass-spectrometry (Figures SI-11 to SI-14). Although an aDAR = 4 mixture was not achieved in this particular case, a Trastuzumab conjugate with DAR = 4 was previously reported with a related rebridging reagent.^23^ As previously reported,^37^ half antibody species were detected in SDS-PAGE and LC-MS analyses, believed to originate from cross-linking of the C226–C229 cysteines within each heavy chain rather than C226–C226 and C229–C229 cross-linking across the two heavy chains.

Conjugation of the 3 antibodies (possessing 10 to 12 methionine residues in their primary sequences, see Table SI-1) using oxaziridine **1** (7–10 equiv) led to comparable results, as assessed by LCMS (Figures **SI-1** to **SI-3**). Masses corresponding to oxidation (+16/+[O]) were detected, putatively assigned to methionine or cysteine oxidation products, although these species could not be quantified.

Derivatization of full native antibodies with tyrosine-targeting conjugation reagent **2** has been reported previously, although characterization of the conjugation parameters were not.^28,32^ A preliminary study on the impact of reagent **2** stoichiometry on the aDAR of **αHER2**-, **Het**-, and **αIL4**-conjugates was carried out. Increasing equivalence of triazoledione **2** led to increasing aDAR values for each antibody generating heterogeneous DAR mixtures. For instance, **αHER2** conjugation gave rise to DAR 1–7 species (see Figure 3). Comparing the **αHER2**, **Het**, and **αIL4** mAbs revealed similar aDAR were obtained for the **αHER2** and **Het** mAbs (which share equivalent Fab domains), and a 2–3-fold lower aDAR was obtained for **αIL4** across a range of conditions (Figure SI-8). Investigations in the conjugation profile for **αHER2** and **αIL4** using fragments antigen-binding (Fab) pointed to a differentiated level of conjugation in the complementarity determining regions (CDR) for each of these two antibodies (see SI Section 5a, Figures SI-9 and SI-10).

**Figure 3:**
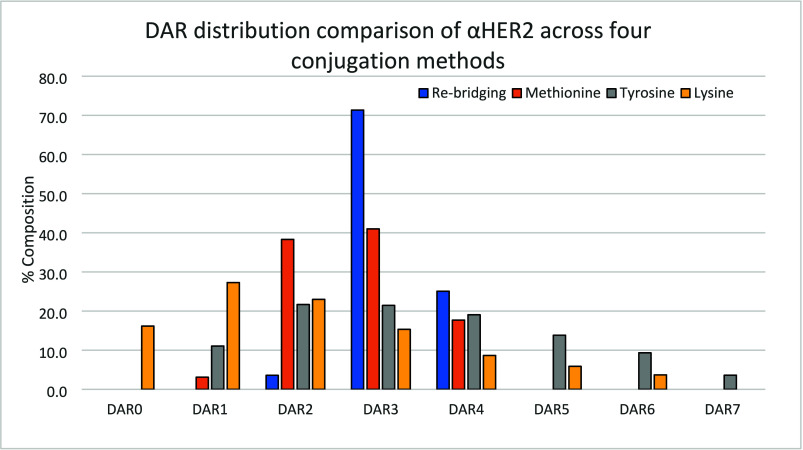
DAR analysis of three conjugation methods applied to **αHER2**. Rebridging conjugation: 20 equiv **3**, 20 equiv TCEP, 18 h, 4 °C, pH 8.5 BBS; methionine conjugation: 10 equiv **1**, 20 h, 4 °C, pH 7.2 PBS; tyrosine conjugation: 50 equiv **2**, 2% organic solvent 72 h, 4 °C, pH 7.2 PBS; lysine conjugation: 10 equiv **4**, 18 h, 4 °C, pH 7.2 PBS).

For comparison, conjugation with a lysine-targeting *N*-hydroxysuccinimide reagent **4** was carried out, targeting a similar aDAR range. It is established that improved selectivity and/or lower aDAR can be achieved in lysine conjugation operating at pH < 8.0.^46^ Using 10 equiv of reagent **4** in pH 7.2 PBS buffer delivered conjugated mAbs with aDAR in the 2–4 range. Under these unoptimized conditions, **αHER2** was consistently less conjugated that the other two mAbs, and all three DAR profiles showed significant heterogeneity in the DAR population (Figure SI-15, **αHER2** data shown on Figure 3).

Next, we sought to understand the potential impact of conjugation on the antigen HER2 binding of **αHER2** (based on Trastuzumab) derived mAbs. To this end, affinity for the soluble extracellular domain of HER2 was measured by SPR for the 3 conjugated HER2-specific mAbs mentioned above. Compared to the unconjugated **αHER2** antibody (*K*_D_ = 0.67 nM) both cysteine and methionine conjugated mAbs remained within 3-fold of their initial binding affinity (*K*_D_ = 0.81 nM and *K*_D_ = 1.35 nM, respectively), whereas the tyrosine conjugated mAb was approximately 10-fold weaker (*K*_D_ = 6.24 nM). The latter result can be rationalized with the observation that conjugation of multiple tyrosines in the Fab region of **αHER2** does occur, potentially including residues directly involved in antigen binding (see SI Section 5a, Figures SI-9 and SI-10), although this was not investigated further.

To appreciate the heterogeneity of the products obtained through the three different conjugation methods studied, a DAR analysis of the species obtained under optimization conditions for each methods with **αHER2** is shown on Figure 3. Tyrosine and lysine conjugation methods led to a wide range of DAR, while the methionine and cysteine rebridging methods provided more narrow distributions.

### Library Generation Using the Modular Platform

In parallel to the preliminary exploration into the bioconjugation methods presented above, the reactive linkers presented in Figure 1 were synthesized (details given in SI). With these in hand, the application of this modular platform for library synthesis could be demonstrated. Three representative payloads were selected: biotin **11**, the E3 ligase VHL binder **12**, and fluorescein **13** covering a range of calculated logP as a representative set (cLogP calculated in the range [−1.21; 1.45], see Table SI-6). In particular, neopentylamine VHL **12** was selected to probe the use of sterically hindered amines. Using the three antibodies previously described (**αHER2, αIL4**, and **Het**), the same three conjugation methods (cysteine rebridging using **3**, tyrosine-targeted using **2**, and methionine targeted using **1**) and the three linker compositions (Valine-citrulline **5** and **8**, Valine-alanine **6** and **9**, PEG-chain **7**, and **10**), 45 out of 81 possible combinations were targeted. In the first step (Figure 4, step A), the mAb was derivatized with the click handle bearing conjugation reagent **1**, **2**, or **3**. Protein recovery and DAR profiles obtained postconjugation were aligned well with the previous results (**αHER2**-cysteine rebridging aDAR = 3.7; **αIL4**-cysteine rebridging aDAR = 3.7; **Het**-cysteine rebridging aDAR = 3.4; **αHER2**-methionine aDAR = 2.7; **αHER2**-tyrosine aDAR = 4.0). In parallel, linker-payload fragments were generated using the efficient carbamate synthesis enabled by the reactive PNP carbonate of reactive linkers **5–7** (Figure 4, **Step B**). Full conversion in this step was achieved as ascertained by LCMS, and the resulting solutions of azide- or strained-alkyne functionalized linker-payload fragments were directly used in a final “click” step to assemble the full ADC (Figure 4, **Step C**).

**Figure 4:**
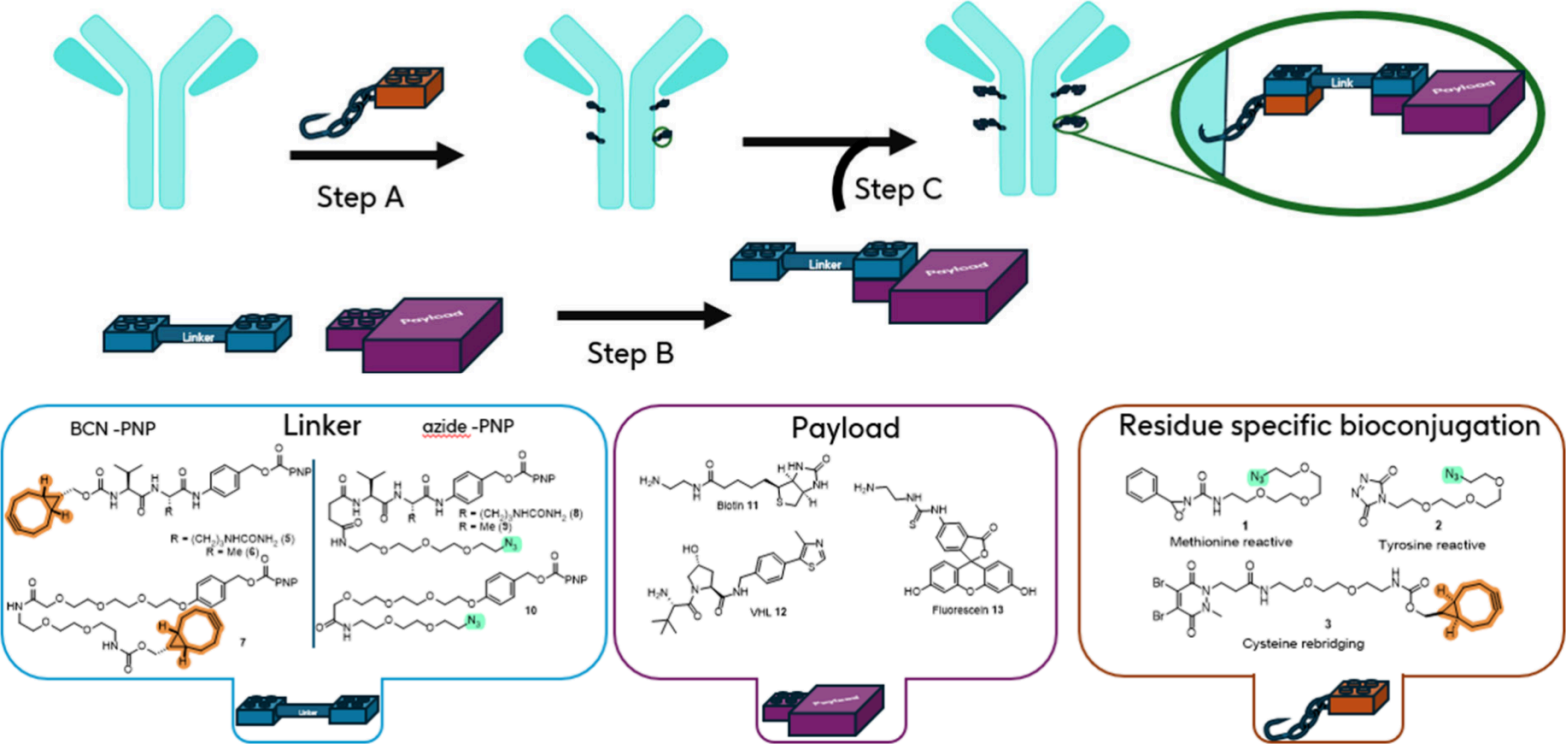
Procedure and composition of the 45 conjugation products library using **αHER2**, **αIL4**, or **Het**. **Step A**: bioconjugation step; using reagent **3** (20 equiv), TCEP (10 equiv), antibody (1000 μL, 3 mg/mL) in pH 8.5 BBS; using reagent **2** (15 equiv), antibody (1000 μL, 3 mg/mL) in pH 7.2 PBS; using reagent **1** (10 equiv), antibody (1000 μL, 3 mg/mL) in pH 7.2 PBS. **Step B**: Linkers **5–10** were combined with Payloads **11–13**, DMSO, rt, 2–24 h, DIPEA (5 equiv) was added when using hydrochloride salts. **Step C**: the payload-linker solutions from Step A (20 equiv) were incubated with the conjugated antibody solution from Step B (40 or 100 μL, 2 mg/mL in PBS) at rt for 24 h.

All resulting conjugates were analyzed by UV–vis and mass spectrometry to establish yields (protein recovery) and DAR analysis, respectively, and the results are summarized in Figure 5. From 45 targeted combinations, 40 are included in this analysis and 5 could not be satisfactorily analyzed due to complex MS signals or poor signal-to-noise in the MS data. Nevertheless, this data set demonstrates the expedient and direct comparison across the bioconjugation methods, linker, and payload used in this experiment.

**Figure 5:**
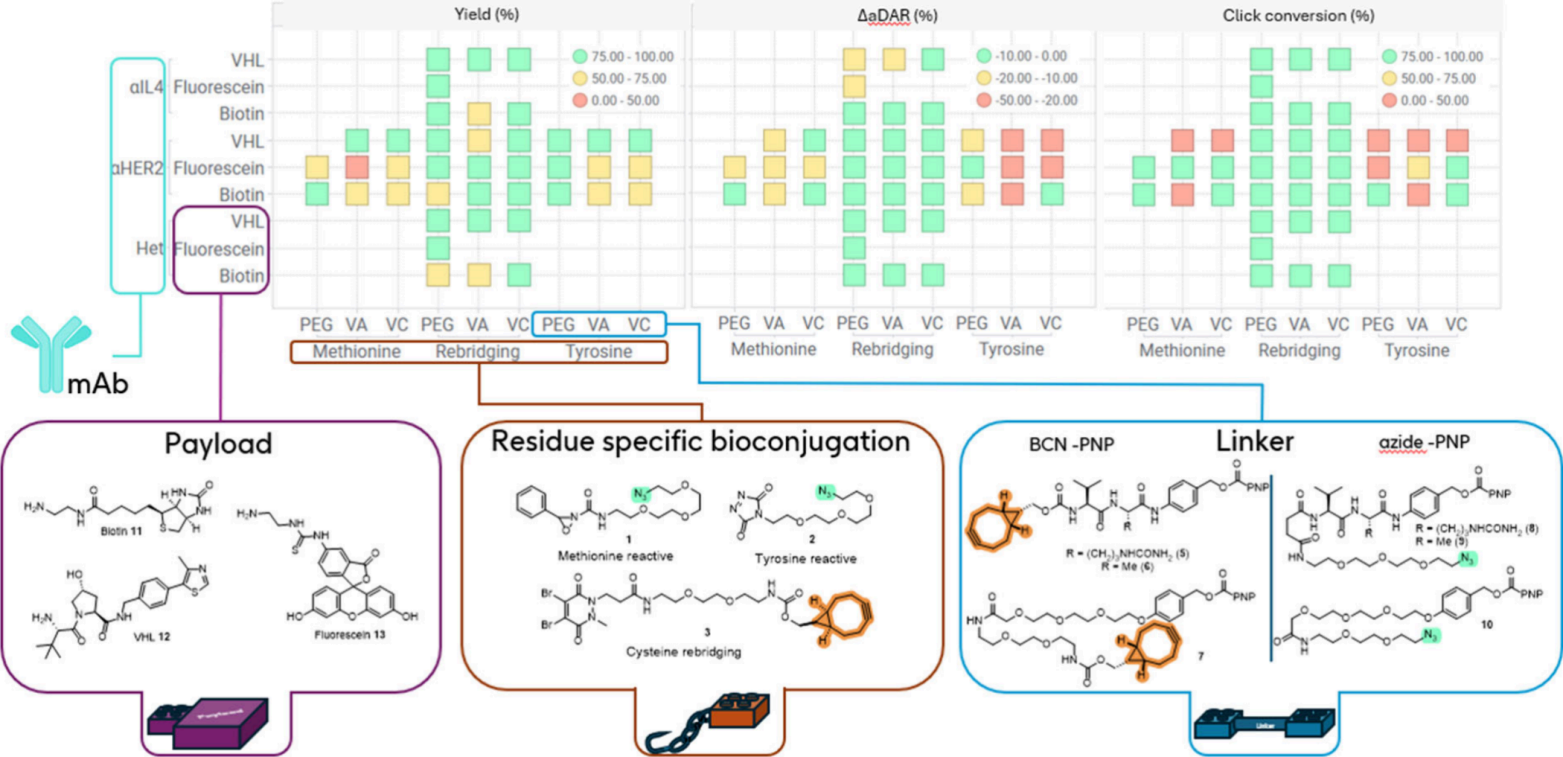
Summary of data obtained by UV–vis and MS analyses for the library of 40 conjugates. Yields (% protein recovery) were determined by UV–vis spectroscopy. ΔaDAR represents the change in aDAR following the final click step. The click conversion was determined by integration of MS signals assigned to the conjugated species showing complete or incomplete attachment of the linker-payload fragment. Linkers used: VA = Valine-alanine, VC = Valine-citrulline, PEG = noncleavable PEG-chain.

First, as evident from the yield data, the protein recovery following the 3-step procedure was in general higher across linker and payloads when using the cysteine rebridging conjugation method, as illustrated by the results obtained for the **αHER2** mAb across the three conjugation methods. Second, particular attention was given to the variation in average DAR (aDAR) before and after “click” attachment of the linker-payload fragment, noting any relative difference in the values as ΔaDAR (%). A high negative ΔaDAR was found to be indicative for the loss of high-DAR species in the mixture post click attachment of the linker-payload, potentially through mechanisms such as protein aggregation. High negative ΔaDAR (%) cases were frequently associated with the samples first conjugated with the tyrosine-targeting reagent **2**, and to a lesser extent to the samples conjugated with methionine-targeting reagent **1**. In contrast, using the cysteine rebridging methodology led to a lower drop in aDAR following the final click step.

These observations can be rationalized when considering the physicochemical properties of the linker-payload used. Specifically, the cysteine rebridging methodology requires the linkers to be functionalized with an azide for the final click step, whereas in the tyrosine and methionine-targeting approaches, the linkers are functionalized with the larger, more lipophilic SPAAC-alkyne (BCN) click handle. As a result, the alkyne-containing linker-payload combinations display a higher calculated logP than the corresponding azide-containing linker-payload combinations (see Table SI-6). Analysis of the clogP and ΔaDAR (%) trend shows that loss of high-DAR species post click step occurs for more lipophilic linker-payload combinations (Figure SI-17).

Finally, the conversion in the click step was derived from DAR analysis, as a number of samples with incomplete conversion following the click step were observed (reaction time: 24 h). As shown in Figure 5, low conversion in the click step was observed only with the tyrosine- and methionine-targeting approaches. Once again, this can be rationalized when considering the physicochemical properties of the linker-payload combination, as the higher lipophilicity of this click reaction partner impacts its aqueous solubility leading to lower click conversion. Analysis of the conversion versus the cLogP trend indeed shows that low conversion in the click step is strongly associated with high cLogP linker-payload combinations (Figure SI-17).

To assess the impact of the click conjugation on antigen binding, the affinity of some of the successfully elaborated prototype ADCs for the soluble extracellular domain of HER2 was tested by SPR. The results obtained did not deviate from the affinity measured for the conjugated ADC preclick step, as demonstrated by PEG-VHL cysteine-conjugated **αHER2** (pre click: *K*_D_ = 0.81 nM; postclick: *K*_D_ = 0.84 nM) and PEG-fluorescein methionine-conjugated **αHER2** (pre click: *K*_D_ = 1.35 nM; postclick: *K*_D_ = 1.20 nM). This illustrates that deconvoluting bioconjugation and payload attachment allows for a robust understanding of the final ADC antigen binding, which is greatly determined by the bioconjugation step.

### Improving the On-Protein Click Step

To explore possible solutions to the issues observed in some cases with the click step, we decided to further examine the combination of Val-Cit-alkyne linker **5** and VHL payload **12** which performed poorly in the library building experiment described in Figure 5 (only 2% conversion in the click step, as determined from MS analysis). Consistent with this previous finding in the array format, no conversion to the desired final ADC was achieved in a “click” reaction between the linker-payload reagent combination **5**–**12** and **met-αHER2** (**αHER2** conjugated with methionine-targeting reagent **1**, structure shown on Figure 5).

Analyzing the composition of the product obtained, species matching partial reaction of the available azide groups conjugated on the mAb were observed (approximately 30% partially clicked species to 65% unreacted **met-αHER2**, Figure 6). To improve the performance of the “click” step, PEG-ylated analogue linker-payload combinations were prepared by the reaction of VHL payload **12** and new reactive linkers **14–16**. A marked improvement of the click step outcome with increasing PEG-chain length was observed, and the 6-PEG chain linker-payload combination **16** and **12** furnished 59% conversion to the fully clicked ADC. Further improvement was achieved by increasing the organic solvent content, leading to a much improved 77% conversion to the desired ADC. This result illustrates the critical importance of considering the solubility of the linker-payload fragment in the relevant medium for the design of ADC libraries using this approach.

**Figure 6:**
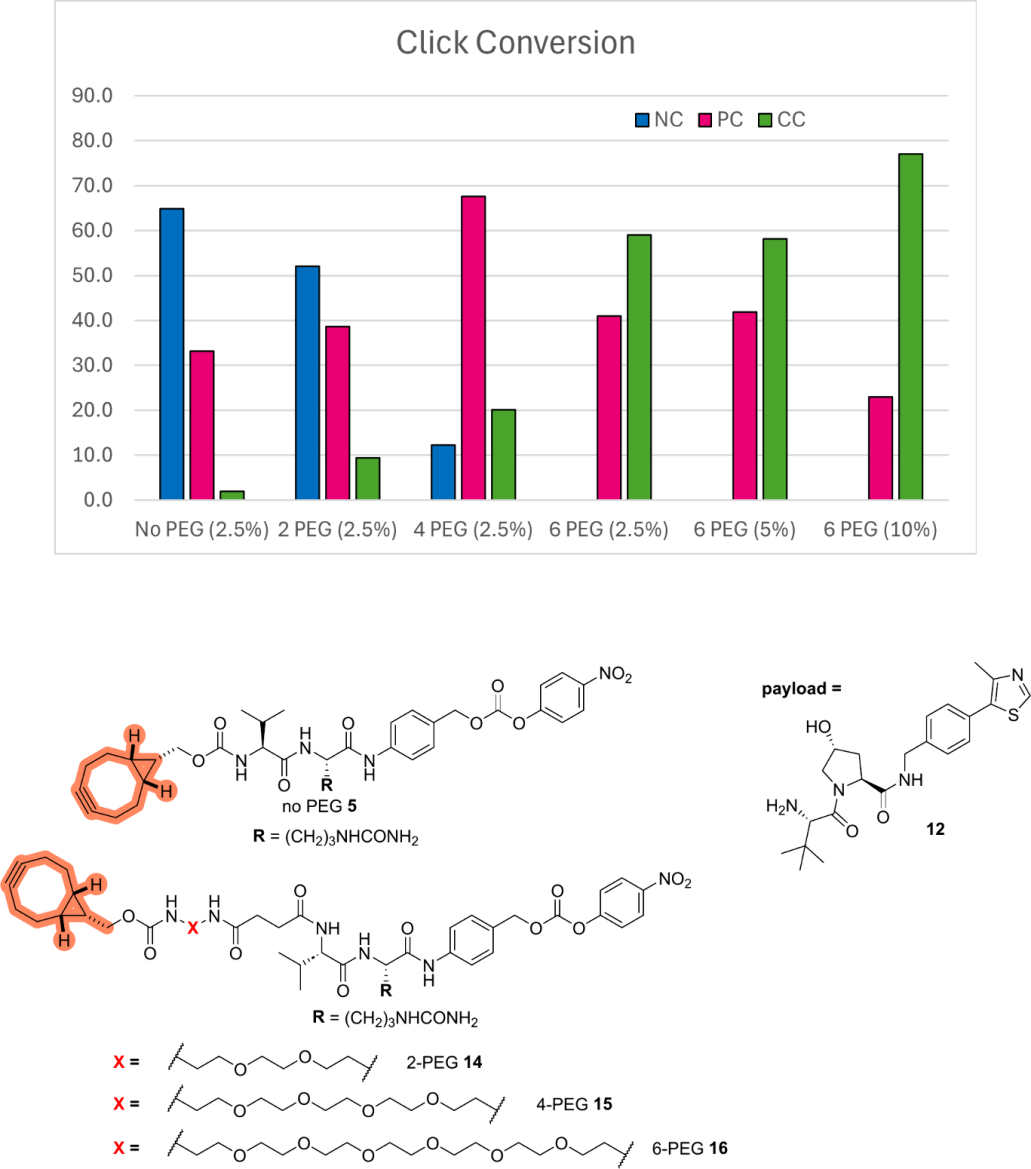
Click conversion of methionine functionalized αHER2 using combinations of linkers **5**, **14–16**, and payload **12** at different percentages of organic solvent, value given within parentheses. NC = non-clicked protein, PC = partially clicked protein, CC = fully clicked protein. aDAR of **αHER2** conjugated with methionine-targeting reagent **1**, **met-αHER2** was 2.7.

### Comparison of Convergent vs Linear Route

Finally, we sought to generate a direct comparison between the convergent and modular approaches developed here and the corresponding linear route. For this, the fully elaborated cysteine rebridging reagent **17** was generated and used to prepare a Val-Cit-Biotin **αIL4** ADC. In parallel, we generated the same ADC using the convergent approach outlined in Scheme 2. The results presented in Table 1 illustrate that the aDAR and DAR ranges obtained for both approaches were very similar. Interestingly, a small deviation from the conjugation protocol used for the cysteine rebridging (the final organic solvent content was 8% here, while it is 2% in the general method used for the experiment described in Figure 5) led to a higher aDAR here (4.24 here vs 3.7 in prior experiment). DAR = 5 and DAR = 6 species were also observed (Figures SI-19 and SI-20) The monofunctionalization of one or several dibromopyridazinedione reagents could rationalize this last observation. Overall, the linear method required extended preparative times (by half a day) in comparison to the convergent approach.

**Scheme 2:**
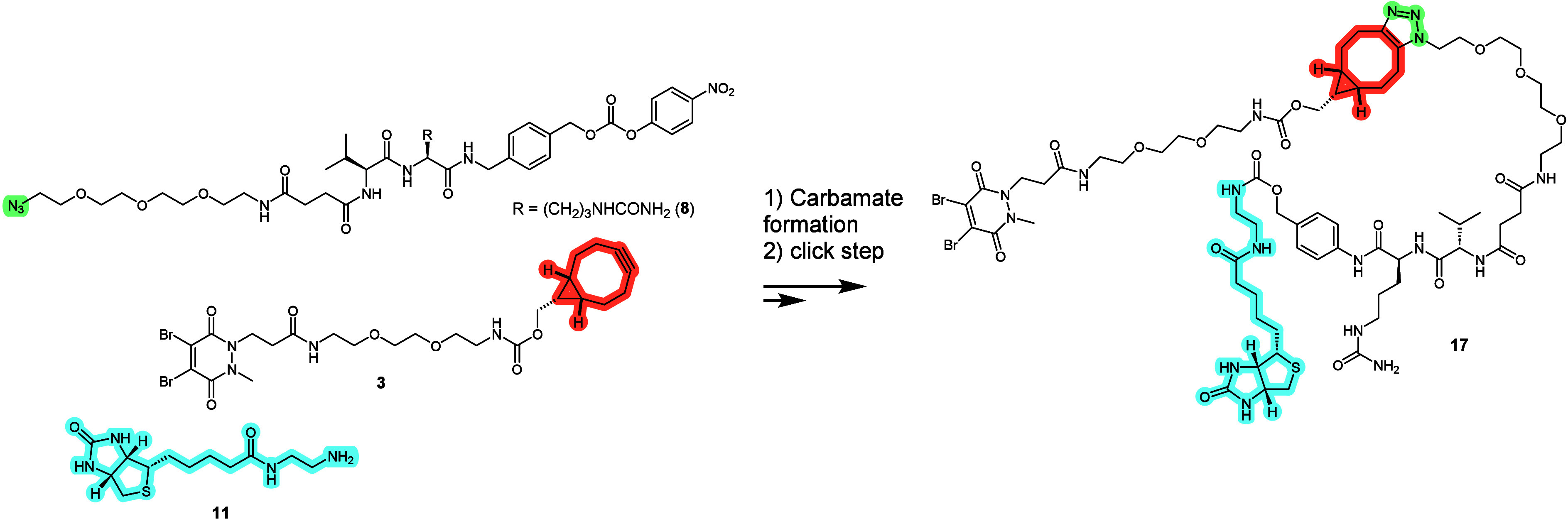
Linear route to payload-bearing conjugation reagent **17**

**Table 1:** Comparison of aDAR and DAR range obtained following a linear route using payload-bearing conjugation reagent **17** or a convergent route with reactive linker **8**, cysteine bioconjugation reagent **3** and payload **11** using the procedure described in Figure 4 to give a Val-Cit-Biotin-**αIL4** ADC

methodology	payload	aDAR	DAR range
Linear	Biotin (**11**) using **17**	4.06	3–6
Convergent	BCN (**3**) only	4.24	3–6
	Biotin (**11**)	4.21	3–6

## Conclusion

The design and implementation of a convergent and modular approach to the preparation of small molecule-antibody conjugates which enables combinatorial variation of each component have been presented. This approach, termed “Stick and Click”, was demonstrated using three therapeutic mAbs, relevant linker technology, nonclassical residue-targeting conjugation manifolds (cysteine rebridging, tyrosine, and methionine-targeting chemistries) along with varied amine-containing small molecule payloads. Following initial optimization for each conjugation method and preparation of the reactive linkers, a library of 40 conjugates was rapidly prepared to demonstrate application of this platform. The principal advantage of this approach over conventional and linear conjugation protocols is that it enables parallel preparation of conjugates allowing facile variation of each component, paving the way for a fully automated tailored ADC preparation process.

Decoupling the bioconjugation and payload attachment steps also enables control of the DAR profile at the first bioconjugation step, as full DAR retention was demonstrated post “Click” attachment of the linker-payload combination in the majority of cases. For this to be realized, particular attention needs to be given to the physicochemical properties of the reactive linkers to ensure a successful click step, namely, minimizing lipophilicity and ensuring sufficient aqueous solubility. To this end, a reactive linker optimization strategy to address poor click conversion is also presented.

Cases where highly cytotoxic payloads are used represent another advantageous use case for this convergent approach, as it minimizes the number of steps carrying material subjected to strict exposure limits and obviates the need for purification of cytotoxin-containing small molecules. Finally, this work also offers a rare comparison of nonclassical conjugations methods to achieve comparable ADC constructs, highlighting the cysteine rebridging method employing dibromopyridazinediones as particularly appropriate for this modular approach.

## Materials and Methods

### Materials

All material used in this study were obtained from commercial suppliers, unless stated. ((1R,8S,9s)-Bicyclo[6.1.0]­non-4-yn-9-yl)­methyl (2,5-dioxopyrrolidin-1-yl) carbonate (endo-BCN-NHS carbonate) was obtained from Sichem. Other reagents were obtained from Merck KGaA or CombiBlocks.

### Small Molecule Synthesis

The synthesis routes for small molecule components used here, including linkers **5–10** and bioconjugation reagents **1–4**, are shown in Schemes SI-1, SI-2, SI-3, SI-6, and SI-10. The detailed preparations and analytical characterizations are given in the relevant sections of the Supporting Information.

### Preparative HPLC

Automated preparative HPLC was carried out on either Teledyne ISCO EZPrep or ACCQPrep using a Sunfire or Xselect C18 column (150 mm × 30 mm internal diameter, 5 μm packing diameter) at ambient temperature, utilizing an appropriate solvent system and elution gradient as determined by analytical LCMS (acetonitrile/water with one of the following modifier: formic acid, trifluoroacetic acid, or ammonium bicarbonate).

### Mass Directed Auto-Preparative (MDAP) HPLC

MDAP purifications were conducted on a Waters FractionLynx system comprising a Waters 600 pump with extended pump heads, Waters 2700 autosampler, Waters 996 diode array, and Gilson 202 fraction collector. The high-performance liquid chromatography (HPLC) separation was conducted on a Sunfire or Xselect C18 column (150 mm × 30 mm internal diameter, 5 μm packing diameter) at ambient temperature, utilizing an appropriate solvent system and elution gradient as determined by analytical LCMS (i.e., formic acid, trifluoroacetic acid, or ammonium bicarbonate modifier). Mass spectra were recorded on a Waters ZQ mass spectrometer using alternate-scan positive and negative electrospray ionization (ES^+^ and ES^–^) with a scan range of 100 to 1000 AMU, scan time of 0.27 s, and interscan delay of 0.05 s. The software used was MassLynx 3.5 with FractionLynx 4.1.

### High Resolution Mass Spectrometry (HRMS)

High-resolution mass spectra were recorded on a Waters XEVO G2-XS quadrupole time-of-flight mass spectrometer, using positive electrospray ionization (ES^+^) with a scan range of 100 to 1200 AMU. Analytes were separated using an Acquity UPLC CSH C18 column (100 mm × 2.1 mm, 1.7 μm packing diameter). UPLC conditions were 0.8 mL/min flow rate, 50 °C, and injection volume 0.2 μL. Gradient elution with (A) water containing 0.1% (v/v) formic acid and (B) acetonitrile containing 0.1% (v/v) formic acid. Gradient conditions were initially 3% B, increasing linearly to 100% B over 8.5 min, remaining at 100% B for 0.5 min then decreasing linearly to 3% B over 0.5 min followed by an equilibration period of 0.5 min prior to the next injection. Mass to charge ratios (*m*/*z*) are reported in atomic mass units with error in ppm.

### Analytical LCMS

Reaction mixtures and products were analyzed by LCMS using either method A or B: *Method A*: Column 50 mm × 2.1 mm ID, Acquity UPLC BEH C18 column. Flow rate 1 mL/min. Temperature 40 °C, UV detection range 210 to 350 nm. Mass spectrum: Recorded on a mass spectrometer using alternate-scan positive and negative mode electrospray ionization. Solvents A: 10 mM solution of ammonium bicarbonate in water, B: acetonitrile; the following gradient was used: *t* = 0 min 99% (A): 1% (B); *t* = 1.5 min 3% (A) :97% (B); *t* = 1.9 min 3% (A): 97% (B); *t* = 2.0 min 99% (A): 1% (B); *Method B*: Column 50 mm × 2.1 mm ID, Acquity UPLC BEH C18 column. Flow rate: 1 mL/min. Temperature 40 °C. UV detection range 210 to 350 nm. Mass spectrum: Recorded on a mass spectrometer using alternate-scan positive and negative mode electrospray ionization. Solvents A: 0.1% v/v solution of formic acid in water, B: 0.1% v/v solution of formic acid in acetonitrile; the following gradient was used: *t* = 0 min. 97% (A): 3% (B); *t* = 1.5 min 0% (A): 100% (B); *t* = 1.9 min 0% (A): 100% (B); *t* = 2.0 min 97% (A): 3% (B).

### NMR

^1^H NMR spectra were recorded in either CDCl_3_, CD_3_OD, or CD_3_SOCD_3_ on either a Bruker AV or DXP 400/500/600 spectrometer working at 400, 500, and 600 MHz, respectively. Chemical shifts (δ) are reported in parts per million (ppm), and coupling constants are reported in Hz and refer to ^3^ *J*_H–H_ interactions unless otherwise stated. Couplings are defined as singlet = s, doublet = d, triplet = t, quartet = q, broad = br, apparent = app. Chemical shifts (δ) are reported in parts per million (ppm). The internal standard used was either tetramethylsilane or the residual protonated solvent, at 7.26 ppm for CDCl_3_ or 2.50 ppm for DMSO-*d*_6_. ^13^C NMR spectra were recorded in either CDCl_3_ or DMSO-*d*_6_ on either a Bruker AV or DXP 400/500/600 spectrometer working at 101, 126, and 151 MHz, respectively.

### SDS-PAGE (Sodium Dodecyl Sulfate–Polyacrylamide Gel Electrophoresis)

Sample was mixed 3:1 with 4× LDL sample buffer (Invitrogen, NP0007) of which 15 μL was loaded per well of a 15 or 17-well, 4–12% NuPAGE SDS-PAGE gel (Invitrogen, NP0336 or NP0329, respectively). A 5 μL portion of Novex Sharp prestained protein standard (Invitrogen, LC5800) was added to an empty well as a standard size marker. Samples were then separated by electrophoresis for 45 min at a constant 200 V in 1× MES buffer (Invitrogen, NP0002). The gel was then stained overnight in Instant Blue Coomassie stain (Sigma, ISB1).

### Protein Mass Spectrometry General Procedure

Sample containing approximately 10 pmol of protein was injected onto a Zorbax Poroshell 300SB-C3 guard column (2.1 mm × 12.5 mm), desalted by washing with 0.1% formic acid in 5% acetonitrile, and then eluted with 0.1% formic acid in 90% acetonitrile at a flow rate of 0.5 mL min^–1^. The eluent was directed to the ESI interface (a standard Z-spray source fitted with an electrospray probe) of a Micromass Q-Tof API-US mass spectrometer. The source temperature and desolvation temperature were set to 100 and 150 °C, respectively. The capillary voltage was 3.0 kV, and the sample cone voltage was 35 V. The mass spectrometer was routinely calibrated using the ion series from a solution of 0.05% phosphoric acid in 50% isopropanol. A calibrant sample (0.05% phosphoric acid in 50% isopropyl alcohol) was injected at the start of each run to enable mass correction of the charge envelope against external standards. External mass correction is performed by comparing observed values for two of the principal ions of the phosphoric acid ion series with the true *m*/*z* values, then applying a modified instrument calibration to correct the *m*/*z* spectrum for any differences. To ensure that software-generated masses accurately represent the components of each sample, raw data were externally mass corrected, as necessary, before deconvolution using the MaxEnt 1 algorithm of MassLynx, version 4.1.

Unless otherwise indicated, samples were deglycosylated using PNGaseF, the general procedure for which was as follows: 0.5 μL of PNGaseF was added to a solution of antibody (25 μL, 1 mg/mL in 7.2 pH PBS), and the samples were incubated at 37 °C for 3 h or at rt for 20 h. Those samples that have not undergone deglycosylation will be stated.

### Determination of Protein Concentration

Absorption of sample at 280 nm was measured using a Nanodrop ND-1000 or Nanodrop One, from which the concentration was calculated based on the theoretical extinction coefficient of the Ab using the formula described by Pace et al.^47^

$$C=\frac{A\left(280\right)}{\varepsilon {\left(280\right)}^{*}l}$$

where *C* = concentration (M), *A*_280_ = absorption at 280 nm, ε_280_ = molar extinction coefficient at 280 nm, calculated as ε = 5500*­(#Tryptophan) + 1490*­(#Tyrosine) + 125*­(#Cystine), *l* = cell length.

### Drug-Antibody Ratio Calculation

aDAR values were calculated using the response intensity from the mass spectrum associated with the appropriate mass adduct, with an error of ±40 D to account for minor fluctuations and any possible post-translational modifications. For samples containing the glycan, the three major glycoforms were summated to provide the total intensity for the specific DAR. For cysteine rebridged species, the half bodies species were not included in the aDAR calculation.

aDAR was then calculated using the following formula;

$$aDAR=\frac{0^{*}DAR0+1^{*}DAR1+2^{*}DAR2+\cdot \cdot \cdot nDARn}{DAR0+DAR1+DAR2+\cdot \cdot \cdot DARn}$$

where DAR*n* = intensity response for a given DAR value, *n*.

Click conversions were determined from the DAR analysis, where species showing incomplete conversion of the on-protein click handles were observed on the deconvoluted mass spectrum. Response intensities from the mass spectrum were aggregated for full clicked species (**CC**, masses corresponding to mAb + ***x*** [bioconjugation reagent] + ***x*** [click partner]), partially clicked species (**PC**, masses corresponding to mAb + ***x*** [bioconjugation reagent] + ***x-n*** [click partner], where ***n*** is 1, 2, . . ., ***x***-1), and non-clicked species (**NC**, masses corresponding to mAb + ***x*** [bioconjugation reagent]). The conversion of the click step (% click) was calculated using the following formula:

$$\%\;click=\frac{FC}{FC+CC+NC}$$

### Antibody Generation General Procedure

Antibodies were generated in mammalian cell lines (HEK293-6E) by transient transfection of cells in the exponential growth phase with mammalian expression plasmids encoding antibody heavy and light chains (1:1 HC:LC) using a lipid transfection reagent and cultured for approximately 1 week at 37 °C, 5% CO_2_ with shaking at 125 rpm.

Cell supernatants were harvested by centrifugation, from which antibodies were purified by protein A affinity chromatography followed by size exclusion chromatography. The fractions were analyzed by SDS-PAGE and analytical SEC. The appropriate fractions were combined and buffer exchanged to PBS dilution/ultrafiltration (using Merck Millipore Amicon Ultra Centrifugal filters, 30 kDa molecular weight cutoff, PES membrane).

Purification: The supernatant was purified by a protein A column, followed by size exclusion chromatography. The fractions were analyzed by SDS-PAGE and ASEC. The appropriate fractions were combined and buffer exchanged to PBS via centrifuge (using Merck Millipore Amicon Ultra Centrifugal filters, 30 kDa molecular weight cutoff, PES membrane).

### General Methionine Conjugation Procedure

The following general procedure is defined for methionine conjugation: A solution of oxaziridine **1** (7–10 molar equiv, 10 mM in MeCN) was added to antibody (3 mg/mL, 20 μM, in pH 7.2 PBS), and the reaction was incubated at 4 °C for 16 to 20 h. After this time, the reaction mixture was diluted with PBS (minimum 2 fold dilution) and purified by ultrafiltration (30 kDa MW cutoff PES membrane, 500 μL, 5 × 5 min @ 12000 rcf, 20 °C). The filtrand was diluted to the nearest graduation, value recorded to give yield, and transferred to an Eppendorf vial. The samples were analyzed by UV–vis to determine the protein concentration, as well as mass spectrometry and SDS-PAGE.

### General Tyrosine Conjugation Procedure

A solution of triazoledione **2** (2 μL, *N* molar equivalents, *N* mM in MeCN, 2% total organic solvent in the reaction mixture; to ensure that the total percentage of organic solvent is consistent, the concentration of the reagent in acetonitrile must be varied. For example, to achieve 20 mol equiv of **2** a 20 mM solution must be used.) was added to antibody (100 μL, 3 mg/mL, 20 μM, in pH 7.2 PBS), and the reaction was incubated at 4 °C for 16–20 h. After this time, the reaction mixture was diluted with PBS (minimum 2 fold dilution) and purified by ultrafiltration (30 kDa MW cutoff PES membrane, 500 μL, 5 × 5 min @ 12000 rcf, 20 °C). The filtrand was diluted to the nearest graduation, value recorded to give yield, and transferred to an Eppendorf vial. The samples were analyzed by UV–vis to determine the protein concentration, as well as mass spectrometry and SDS-PAGE.

### General Procedure for Cysteine Rebridging

A solution of pyridazinedione **3** (20 molar equiv, 20 mM in DMSO) was added to antibody (3 mg/mL, 20 μM, in pH 8.5 BBS), and the reaction was incubated at 4 °C for 1 h. After this time, a solution of TCEP (2 μL, 10 molar eq, 10 mM in pH 8.5 BBS) was added, and the reaction was incubated for a further 16 to 20 h. After this time, the reaction was diluted with PBS (minimum 2-fold dilution) and purified by ultrafiltration (30 kDa MW cutoff PES membrane, 500 μL, 5 × 5 min @ 12000 rcf, 20 °C). The filtrand was diluted to the nearest graduation, the value was recorded to give yield, and it was transferred to an Eppendorf vial. The samples were analyzed by UV–vis to determine the protein concentration, as well as mass spectrometry and SDS-PAGE.

### General Lysine Conjugation Procedure

NHS ester **4** (1 μL for 5 equiv or 2 μL of 10 equiv, 10 mM in DMSO) was added to αIL4, αHER2, or Het solution (100 μL, 3 mg/mL, 20 μM, pH 7.2 PBS), and the reaction was incubated at 4 °C for 18 h. After this time, each reaction was diluted to 500 μL with pH 7.2 PBS and purified by ultrafiltration (4 × 5 min, 12000 rcf, 30 kDa MW cutoff PES membrane). After the final spin, the vials were diluted to the nearest graduation and then transferred to a 500 μL Eppendorf vial. The samples were analyzed by UV–vis to determine the protein concentration, as well as mass spectrometry and SDS-PAGE.

### Library Building Experiments

#### Payload Attachment Chemistry General Procedure

A solution of amine **11–13** (50 μL, 30 mM in DMSO, 1.5 molar equiv or 50 μL, 50 mM in DMSO, 2.5 molar equiv) was added to the activated linker solution (50 μL, 20 mM in DMSO), and the reaction was stirred at r.t. for 2 to 24 h. Reactions were monitored by LCMS for complete consumption of the activated linker. Once complete, the crude reaction mixture was used without further purification as a 10 mM solution of the linker-payload fragment. For hydrochloride salts, triethylamine (5 molar equiv) was added to reaction mixture.

#### On-protein Click Step General Procedure

The linker-payload fragment (10 mM in DMSO, crude, 20 molar equiv) was added to functionalized antibody (2 mg/mL, 13 μM, pH 7.2 PBS), and the reaction was incubated at rt for 24 h. After this time, the reactions were diluted to 500 μL (PBS) and purified by ultrafiltration (30 kDa MW cutoff PES membrane, 500 μL, 8 × 4 min @ 12000 rcf, 20 °C). The filtrand was diluted to the nearest graduation; the value was recorded to calculate yield, and the solution was transferred to an Eppendorf vial. The samples were analyzed by UV–vis to determine the protein concentration, as well as mass spectrometry and SDS-PAGE.

#### Library Building Experiment using Cysteine Rebridging

The bioconjugation procedure followed in accordance with the rebridging general procedure (see above), using 500 μL of each antibody solution (3 mg/mL, 20 μM), 20 μL of rebridging reagent **3** (10 mM in DMSO), and 20 μL of TCEP (10 mM in pH 8.5 BBS). Payload attachment chemistry followed the general procedure, using 20 μL of activated linker (20 mM in DMSO), 20 μL of payload (30 mM in DMSO), and 0.35 μL DIPEA (5 molar equiv) for payload **12**. Click chemistry follows a general procedure, using functionalized antibody (40 μL, 2 mg/mL, 13 μM in PBS), linker-payload fragment (1.2 μL in DMSO, 10 mM, 20 molar equiv).

#### Library Building Experiment Using Three Conjugation Methods

The bioconjugation procedure was followed in accordance with the general procedure described above. For the rebridging procedure, **αHER2** solution (500–1000 μL, 3 mg/mL, 20 μM in pH 8.5 pH BBS), 20 μL of rebridging reagent **3** (10 or 20 mM in DMSO, 20 molar equiv), and 20 μL of TCEP (10 mM in BBS, 10 molar equiv) were used. For the methionine targeted conjugation procedure, **αHER2** solution (1000 μL, 3 mg/mL, 20 μM in pH 7.2 pH PBS) and 20 μL of reagent **1** (10 mM in MeCN, 10 molar equiv) were used. For the tyrosine targeted conjugation procedure, **αHER2** solution (1000 μL, 3 mg/mL, 20 μM in pH 7.2 PBS) and 20 μL of reagent **2** (15 mM in MeCN, 15 molar equiv, 2.0% total organic solvent in the reaction mixture) were used. Payload attachment chemistry followed the general procedure described above, using 50 μL of activated linker solution (20 mM in DMSO), 50 μL of payload solution (30 mM in DMSO), and additionally 1 μL of DIPEA (5 molar equiv) for payload **12**. The Click chemistry step followed the general procedure described above, using the functionalized antibody (50–100 μL, 2 mg/mL in PBS), linker-payload fragment (in DMSO, 10 mM, 20 molar equiv). The reaction was carried out on a 96-well plate.

### HER2 Binding by SPR

Binding of human HER2 to anti-HER2 antibodies and ADC was assessed using nonregenerative capture kinetics on a LSA (Carterra) SPR instrument. Antibodies/ADC were captured on a HC30m biosensor (Carterra) using a standard amine coupling protocol (Cytiva, Amine Coupling Kit type 2). Recombinant human HER2 extracellular domain (Sigma, SRP6405) was prepared in HBS-EP+, pH 7.6 (Teknova) and injected over the captured antibodies as a concentration series including eight 2× serial dilutions with the highest concentration of 32 nM. The association phase was 300 s, followed by a dissociation phase of 900 s. A buffer only (0 nM analyte) injection was used to double reference the binding curves. The assay was run at 25 °C in HBS-EP+ pH 7.6 buffer.

## Supplementary Material



## Notes

Relative expressions of relevant residues for the antibodies used in this study; the chemical synthesis for active linkers and conjugation reagents and copies of NMR and LCMS spectra; the results from initial exploration and optimization of bioconjugation conditions for methionine-targeting, tyrosine-targeting, cysteine-targeting, and lysine-targeting methods; summary table for the library building experiment presenting mass spectroscopy, yield and click conversion data; SDS-PAGE analysis; cLogP calculations (PDF)
