# Supplementary material for: A Modular and Convergent “Stick and Click” Conjugation Platform Enables Fast Antibody Conjugate Library Synthesis
Source: Bioconjug Chem. 2026 Jan 7;37(1):40–51. doi: 10.1021/acs.bioconjchem.5c00461 (PMC12863055; doi:10.1021/acs.bioconjchem.5c00461)
Supplement: Supplementary file 1 [file bc5c00461_si_001.pdf]

A modular and convergent "Stick and Click" conjugation platform enables fast antibody conjugate library synthesis and a direct comparison of residue specific conjugation methods.

Connor Livingstone,[1,2] Dr. Simon Nicolle,*[1] Dr. Gavin Jones,[1] Prof. Craig Jamieson[2]

*corresponding author
[1]GSK
Medicines Research Centre, Gunnels Wood Road, Stevenage, UK, SG1 2NY
E-mail: simon.x.nicolle@gsk.com

[2]Dept. of Pure and Applied Chemistry
University of Strathclyde
295 Cathedral Street, Glasgow, UK, G1 1XL

1. Antibodies targeted: Relative expression of relevant residues 2
2. Synthesis of exemplar payloads 3
3. Active Linker synthesis 5
   a. Protease-cleavable linkers Val-Cit and Val-Ala compounds **5,6,8,9** 5
   b. Non-cleavable linkers **7** and **10** 22
4. Methionine-targeting bioconjugation 28
   a. Preparation of methionine conjugation compound 1 28
   b. Methionine bioconjugation to several antibodies 29
5. Tyrosine-targeting bioconjugation 31
   a. Synthesis of tyrosine conjugation reagent **2** 31
   b. Reagent selection and preparation 32
   c. Bioconjugation trial with tyrosine conjugation reagents **2** and **C** 34
   d. Exploration of tyrosine-targeted bioconjugation using reagent **2** 35
   e. Understanding the aDAR difference between antibodies αHER2 and αIL4 36
6. Re-bridging Cysteine conjugation 38
   a. Preparation of re-bridging reagent **3** 38
   b. Evaluation of cysteine re-bridging conjugation 41
7. Lysine conjugation 43
   a. Preparation of reagent **4** 43
   b. Bioconjugation procedure 45
8. Library building experiments Figure 5 45
   a. Summary table 45
   b. SDS-PAGE 48
   c. Relation between aDAR changes, click conversion and calculated LogP of the linker-payload fragment 49

9. Improving the Click conversion 50
10. Comparison of linear and convergent approach to a Val-Cit-Biotin αIL4 ACD and a Val-Cit-Fluorescein αIL4 ACD 51
a. Linear approach 51
b. Convergent Approach 52
c. SDS-PAGE – convergent vs linear approach 53
11. HNMR and LCMS data for key compounds 1-10 54

Safety/hazard statement: no unexpected or unusually high safety hazards were encountered

This manuscript does not present animal and/or human studies

1. Antibodies targeted: Relative expression of relevant residues

Amino Acid	Ab domain	# residues		
		**αIL4 ***	**αHER2 $**	**Het^**
Tyrosine	Whole antibody	56	62	64
	∟ HC	18	21	22
	∟ VH	6	9	9
	∟ HC CDR	4	6	6
	∟ LC	10	10	10
	∟ VL	6	6	6
	∟ LC CDR	2	2	2
Methionine	Whole antibody	12	10	10
	∟ HC	5	4	4
	∟ VH	3	2	2
	∟ HC CDR	1	1	1
	∟ LC	1	1	1
	∟ VL	1	1	1
	∟ LC CDR	1	0	0
Lysine	Whole antibody	94	90	86
	HC	33	32	30
	∟ VH	5	4	4
	∟ HC CDR	2	1	1
	LC	14	13	13
	∟ VL	6	5	5
	∟ LC CDR	1	0	0
Cysteine	Whole antibody	32	32	32
	∟ HC	11	11	11
	∟ VH	2	2	2
	∟ HC CDR	0	0	0
	∟ LC	5	5	5
	∟ VL	2	2	2
	∟ LC CDR	0	0	0

Table SI-1. Comparison of the distribution of Bioconjugation-Targeted Residues across Test Antibodies *__αIL4__ antibody is based on Pascolizumab (UNII N1IOA09R6A) but contains single P to L

change in the VH domain and switch from nG1m17, nG1m1 to G1m17,1 IgG1 allotype. $**αHER2** antibody sequence is based on Trastuzumab (UNII P188ANX8CK) and shares the same variable domain but with a switch from G1m17, nG1m1 to G1m17, 1 IgG1 allotype. ^**Het** antibody sequence is based on Trastuzumab (UNII P188ANX8CK) and shares the same variable domain but possesses multiple modifications to the constant region to promote heterodimerisation.

2. Synthesis of exemplar payloads

2,5-Dioxopyrrolidin-1-yl 5-((3a*S*,4*S*,6a*R*)-2-oxohexahydro-1*H*-thieno[3,4-*d*]imidazol-4-yl)pentanoate

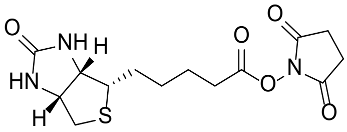

N-hydroxysuccinimide (104 mg, 0.9 mmol) was added to a solution of EDC (188 mg, 0.98 mmol) and 5-((3a*S*,4*S*,6a*R*)-2-oxohexahydro-1*H*-thieno[3,4-*d*]imidazol-4-yl)pentanoic acid (200 mg, 0.819 mmol) in DMF (5 mL) and the reaction mixture was stirred at r.t. for 16 h. After this time the reaction mixture was concentrated under reduced pressure. The resulting solid was suspended in cold ethanol (5 mL) and then filtered under vacuum. The filtrand was further washed with cold ethanol (5 x 20 mL) and then dried in air and under vacuum to afford 2,5-dioxopyrrolidin-1-yl 5-((3a*S*,4*S*,6a*R*)-2-oxohexahydro-1*H*-thieno[3,4-d]imidazol-4-yl)pentanoate (199 mg, 0.583 mmol, 71.2 % yield) as a white solid.

^{1}H NMR (400 MHz, CD_3SOCD_3, 303 K) δ (ppm) = 6.41 (s, 1H), 6.35 (s, 1H), 4.32 (dd, *J* = 7.5, 5.3 Hz, 1H), 4.24 - 4.08 (m, 1H), 3.15 - 3.07 (m, 1H), 2.90 - 2.78 (m, 5H), 2.68 (t, *J* = 7.3 Hz, 2H), 2.59 (d, *J* = 12.5 Hz, 1H), 1.72 - 1.59 (m, 3H), 1.59 - 1.35 (m, 3H). **^{13}C NMR** (101 MHz, CD_3SOCD_3, 303 K) δ (ppm) = 170.7, 169.4, 163.1, 61.5, 59.7, 55.7, 40.4, 30.5, 28.3, 28.0, 25.9, 24.8.

Consistent with previously reported analytical data.[1]

tert-Butyl (2-(5-((3a*S*,4*S*,6a*R*)-2-oxohexahydro-1*H*-thieno[3,4-*d*]imidazol-4-yl)pentanamido)ethyl)carbamate

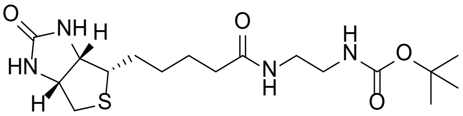

Triethylamine (0.12 mL, 0.86 mmol) and a solution of *tert*-butyl (2-aminoethyl)carbamate (141 mg, 0.879 mmol) in DMF (1 mL) were added to a solution of 2,5-dioxopyrrolidin-1-yl 5-((3a*S*,4*S*,6a*R*)-2-oxohexahydro-1*H*-thieno[3,4-*d*]imidazol-4-yl)pentanoate (150 mg, 0.44 mmol) in DMF (3.5 mL) and the reaction was stirred for 18 h at r.t.. The reaction mixture was concentrated under reduced pressure, redissolved in methanol and adsorbed onto Florisil®. The residue was purified by flash column chromatography (RediSep silica 12 g, 0-10% methanol/DCM and 10 column volumes of 10% methanol in DCM). All fractions containing product were combined and concentrated under reduced pressure. The resulting residue was then dried under vacuum to afford *tert*-butyl (2-(5-((3aS,4S,6aR)-2-

oxohexahydro-1*H*-thieno[3,4-*d*]imidazol-4-yl)pentanamido)ethyl)carbamate (132 mg, 0.31 mmol, 70 % yield) as a white solid.

^{1}H NMR (400 MHz, CD_3SOCD_3, 303 K) δ (ppm) = 7.76 (br t, *J* = 5.4 Hz, 1H), 6.75 (br t, *J* = 5.3 Hz, 1H), 6.41 (s, 1H), 6.34 (s, 1H), 4.33 - 4.29 (m, 1H), 4.14 (ddd, *J* = 7.6, 4.6, 1.7 Hz, 1H), 3.13 - 3.09 (m, 1H), 3.09 - 3.02 (m, 2H), 2.96 (q, *J* = 6.2 Hz, 2H), 2.83 (dd, *J* = 12.5, 5.1 Hz, 1H), 2.59 (d, *J* = 12.2 Hz, 1H), 2.05 (t, *J* = 7.5 Hz, 2H), 1.67 - 1.21 (m, 6H), 1.38 (s, 9H). **^{13}C NMR** (101 MHz, CD_3SOCD_3, 303 K) δ (ppm) = 172.6, 163.2, 156.1, 78.1, 61.5, 59.7, 55.8, 40.3, 39.1, 35.7, 28.70, 28.66, 28.5, 25.7. 1C not observed, suspected to be under the solvent peak.

Consistent with previously reported analytical data.[1]

N-(2-Aminoethyl)-5-((3*aS*,4*S*,6*aR*)-2-oxohexahydro-1*H*-thieno[3,4-*d*]imidazol-4-yl)pentanamide 11

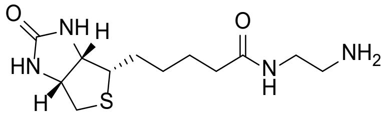

4M HCl in 1,4-dioxane (524 µL, 2.1 mmol) was added to *tert*-butyl (2-(5-((3a*S*,4*S*,6a*R*)-2-oxohexahydro-1*H*-thieno[3,4-*d*]imidazol-4-yl)pentanamido)ethyl)carbamate (90 mg, 0.21 mmol) and the suspension was stirred at r.t. for 20 h. The reaction mixture was concentrated und er reduced pressure to afford an off-white gum. This residue was dissolved in methanol (5 mL) and water (~1 mL) and then loaded onto an SPE NH_2 cartridge (2g, primed with methanol) and eluted using methanol (5 x 25 mL). The filtrate was collected and concentrated under reduced pressure and then dried under vacuum to afford *N*-(2-aminoethyl)-5-((3aS,4S,6aR)-2-oxohexahydro-1*H*-thieno[3,4-*d*]imidazol-4-yl)pentanamide **11** (55 mg, 0.19 mmol, 92 % yield) as a white solid.

^{1}H NMR (400 MHz, CD_3SOCD_3, 303 K) δ (ppm) = 7.73 (br t, *J* = 5.1 Hz, 1H), 6.42 (s, 1H), 6.35 (s, 1H), 4.31 (dd, *J* = 7.6, 5.1 Hz, 1H), 4.18 - 4.09 (m, 1H), 3.15 - 3.07 (m, 1H), 3.03 (q, *J* = 6.4 Hz, 2H), 2.83 (dd, *J* = 12.5, 5.1 Hz, 1H), 2.61 - 2.53 (m, 3H), 2.07 (t, *J* = 7.5 Hz, 2H), 1.67 - 1.57 (m, 1H), 1.57 - 1.42 (m, 3H), 1.41 - 1.22 (m, 2H), 1.18 (t, *J* = 7.1 Hz, 1H). **^{13}C NMR** (101 MHz, CD_3SOCD_3, 303 K) δ (ppm) = 172.5, 163.2, 61.5, 59.7, 55.9, 42.6, 41.8, 40.3, 35.7, 28.7, 28.5, 25.8.

Consistent with previously reported analytical data.[2]

1-(2-Aminoethyl)-3-(3',6'-dihydroxy-3-oxo-3*H*-spiro[isobenzofuran-1,9'-xanthen]-5-yl)thiourea 13

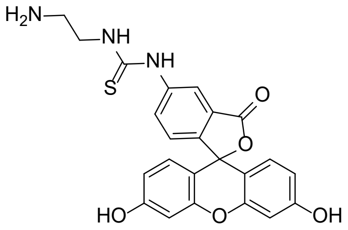

A solution of ethane-1,2-diamine (26 µL, 0.385 mmol) in methanol (6 mL) was added dropwise over 2 minutes to a solution of 3',6'-dihydroxy-5-((thioxo-l5-methylene)amino)-3*H*-spiro[isobenzofuran-1,9'-xanthen]-3-one (100 mg, 0.257 mmol) in methanol (4 mL). The reaction was then stirred at r.t. for 2 h. The reaction mixture was concentrated under reduced pressure and the residue was resuspended in

~2 mL of methanol and filtered by gravity. The filtrand was washed with methanol (4 x 10 mL) and then left to dry in air to afford 1-(2-aminoethyl)-3-(3',6'-dihydroxy-3-oxo-3*H*-spiro[isobenzofuran-1,9'-xanthen]-5-yl)thiourea **13** (88 mg, 0.196 mmol, 76 % yield) as a dark orange solid.

^{1}H NMR (400 MHz, CD_3SOCD_3, 303 K) δ (ppm) = 8.36 - 8.16 (m, 1H), 7.87 - 7.64 (m, 1H), 7.23 - 7.02 (m, 1H), 6.67 (d, *J* = 8.8 Hz, 2H), 6.59 - 6.54 (m, 2H), 6.51 (dd, *J* = 2.3, 8.9 Hz, 2H), 3.65 - 3.42 (m, 2H), 2.81 (t, *J* = 6.1 Hz, 2H) **^{13}C NMR** (101 MHz, $CDCl_3$, 303 K) δ (ppm) = 185.8, 173.7, 164.7, 161.7, 157.1, 153.9, 146.6, 134.2, 131.8, 129.3, 117.8, 115.0, 107.5, 104.2, 74.8, 74.8, 74.4, 66.6, 48.9, 33.8, 26.1, 24.8, 22.9. Multiple species present, possible degradation or ring opening of lactone. ^{13}C NMR not provided in original report.[3] **LCMS**: Formic, RT = 0.55 min. $[M+H]^+$ = 450.2, 90% pure by UV.

3. Active Linker synthesis

a. Protease-cleavable linkers Val-Cit and Val-Ala compounds **5,6,8,9**

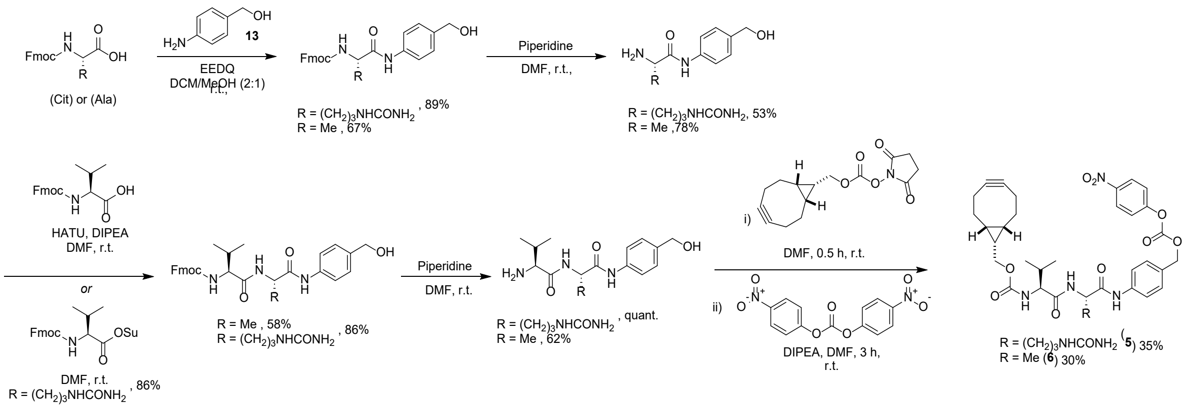

Scheme SI-1. Synthesis route for the preparation of protease-cleavable BCN-functionalised reactive linkers **5** and **6**

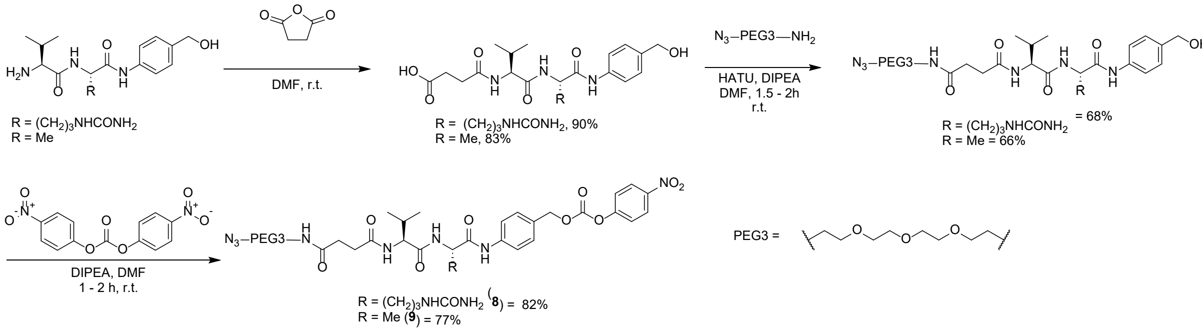

Scheme SI-2. Synthesis route for the preparation of protease-cleavable azide-functionalised reactive linkers **8** and **9**

9*H*-Fluoren-9-yl)methyl (*S*)-(1-((4-(hydroxymethyl)phenyl)amino)-1-oxo-5-ureidopentan-2-yl)carbamate

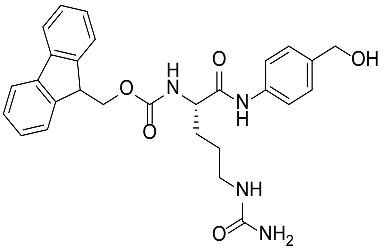

(4-Aminophenyl)methanol (1.12 g, 9.1 mmol) was added to a solution of (*S*)-2-((((9*H*-fluoren-9-yl)methoxy)carbonyl)amino)-5-ureidopentanoic acid (3 g, 7.6 mmol) in DCM (42 mL) and methanol (21 mL). Ethyl 2-ethoxyquinoline-1(2*H*)-carboxylate (2.80 g, 11 mmol) was added, and the reaction was stirred for 18.5 h. After this time the reaction mixture had solidified to a gel. The reaction was diluted with diethyl ether (150 mL) and the mixture was then stirred for 45 min. This was then filtered under gravity, washed with diethyl ether (3 x 75 mL) and the solid was collected and dried under vacuum to afford (9*H*-fluoren-9-yl)methyl (*S*)-(1-((4-(hydroxymethyl)phenyl)amino)-1-oxo-5-ureidopentan-2-yl)carbamate (3.36 g, 6.7 mmol, 89 % yield) as a white solid.

^{1}H NMR (400 MHz, CD_3SOCD_3, 303 K) δ (ppm) = 9.97 (s, 1H), 7.89 (d, *J* = 7.6 Hz, 2H), 7.81 - 7.70 (m, 2H), 7.68 - 7.62 (m, 1H), 7.62 - 7.53 (m, 2H), 7.45 - 7.29 (m, 4H), 7.25 (d, *J* = 8.6 Hz, 2H), 6.00 (br t, *J* = 5.5 Hz, 1H), 5.42 (s, 2H), 5.09 (t, *J* = 5.7 Hz, 1H), 4.45 (d, *J* = 5.7 Hz, 2H), 4.33 - 4.16 (m, 4H), 3.13 - 2.92 (m, 2H), 1.78 - 1.54 (m, 2H), 1.54 - 1.36 (m, 2H) **^{13}C NMR** (101 MHz, CD_3SOCD_3, 303 K) δ (ppm) = 171.4, 159.4, 156.6, 144.4, 144.3, 141.2, 138.0, 137.9, 128.1, 127.5, 127.4, 125.8, 120.6, 119.4, 66.2, 63.1, 55.4, 47.2, 39.0, 29.8, 27.4. Additional C from slight inequivalence of dibenzofulvene. **LCMS** formic, rt = 0.94 mins, $[M+H]^+$ = 503.2, **IR** (ATIR, cm^{-1}): 3279 (br.), 1652, 1531, 1449, 1414, 1280, 1251, 1084, 1033, 738, 645, 532.

Consistent with previously reported analytical data.[4, 5]

(*S*)-2-Amino-*N*-(4-(hydroxymethyl)phenyl)-5-ureidopentanamide

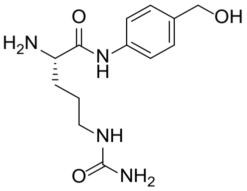

Piperidine (0.12 mL, 1.2 mmol) was added to a solution of (9*H*-fluoren-9-yl)methyl (*S*)-(1-((4-(hydroxymethyl)phenyl)amino)-1-oxo-5-ureidopentan-2-yl)carbamate (250 mg, 0.50 mmol) in DMF (2.5 mL) and the reaction was stirred, at r.t. for 1 h. The reaction was diluted with water (100 mL) and cooled in an ice bath before being filtered under vacuum. The residue was then washed with water (3 x 50 mL) and the filtrate was collected and concentrated under reduced pressure to afford a colourless gum. The residue was purified by reverse phase flash column chromatography (C18 30 g, 0-30% 10 mM ammonium carbonate/Acetonitrile). The appropriate fractions were combined and concentrated *in vacuo* to afford (S)-2-amino-*N*-(4-(hydroxymethyl)phenyl)-5-ureidopentanamide (74 mg, 0.26 mmol, 53 % yield) as a colourless solid.

^{1}H NMR (400 MHz, CD_3SOCD_3, 303 K) δ (ppm) = 10.26 - 9.38 (m, 1H), 7.65 - 7.51 (m, 2H), 7.24 (d, *J* = 8.6 Hz, 2H), 5.93 (br t, *J* = 5.5 Hz, 1H), 5.35 (s, 2H), 5.07 (br t, *J* = 5.5 Hz, 1H), 4.43 (d, *J* = 4.9 Hz, 2H), 3.37 - 3.24 (m, 1H), 3.05 - 2.89 (m, 2H), 2.18 - 1.72 (m, 2H), 1.78 - 1.56 (m, 1H), 1.56 - 1.32 (m, 3H) **^{13}C NMR** (101 MHz, CD_3SOCD_3, 303 K) δ (ppm) = 174.8, 159.2, 138.0, 137.7, 127.4, 119.3, 63.1, 55.7, 39.5, 33.1, 27.2, **LCMS** HpH, rt = 0.43 mins, $[M+H]^+$ = 280.1, **IR** (ATIR, cm^{-1}): 3335 (br.), 2932, 1652, 1604, 1538, 1414, 1312, 1014, 826

Consistent with previously reported analytical data.[4]

(9*H*-Fluoren-9-yl)methyl ((*S*)-1-(((*S*)-1-((4-(hydroxymethyl)phenyl)amino)-1-oxo-5-ureidopentan-2-yl)amino)-3-methyl-1-oxobutan-2-yl)carbamate

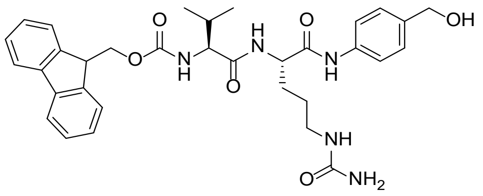

2,5-dioxopyrrolidin-1-yl (((9*H*-fluoren-9-yl)methoxy)carbonyl)-L-valinate (6.5 g, 15 mmol) was added to a solution of (*S*)-2-amino-*N*-(4-(hydroxymethyl)phenyl)-5-ureidopentanamide (5.7 g, 14.2 mmol) in DMF (20 mL) and the reaction was stirred at r.t. for 2 h. The reaction mixture was concentrated under reduced pressure, diluted with ether (500 mL) and then filtered by vacuum and the filtrand was washed with ether (4 x 200 mL) The filtrand was collected in a tared vial and dried under vacuum to afford (9*H*-fluoren-9-yl)methyl ((*S*)-1-(((*S*)-1-((4-(hydroxymethyl)phenyl)amino)-1-oxo-5-ureidopentan-2-yl)amino)-3-methyl-1-oxobutan-2-yl)carbamate (9.2 g, 12 mmol, 86 % yield) (80% purity by NMR) as a beige solid.

^{1}H NMR (400 MHz, CD_3SOCD_3, 303 K) δ (ppm) = 9.96 (s, 1H), 8.16 - 8.01 (m, 1H), 7.89 (d, *J* = 7.4 Hz, 2H), 7.80 - 7.69 (m, 2H), 7.62 - 7.52 (m, 2H), 7.45 - 7.37 (m, 3H), 7.33 (dt, *J* = 7.4, 1.1 Hz, 2H), 7.24 (d, *J* = 8.6 Hz, 2H), 5.97 (br t, *J* = 5.7 Hz, 1H), 5.40 (s, 2H), 5.08 (t, *J* = 5.7 Hz, 1H), 4.44 (m, 3H), 4.36 - 4.20 (m, 3H), 4.00 - 3.90 (m, 1H), 3.10 - 2.89 (m, 2H), 2.12 - 1.91 (m, 1H), 1.76 - 1.56 (m, 2H), 1.56 - 1.33 (m, 2H), 0.94 - 0.82 (m, 6H). **^{13}C NMR** (101 MHz, CD_3SOCD_3, 303 K) δ (ppm) = 171.7, 170.8, 159.3, 156.6, 144.4, 144.2, 141.2, 138.0, 137.9, 128.1, 127.5, 127.4, 125.8, 120.6, 119.4, 66.2, 63.1, 60.6, 53.5, 47.2, 39.1, 30.9, 30.0, 27.2, 19.7, 18.7. **LCMS** HpH, rt = 1.03 mins, $[M+H]^+$ = 602.2.

Consistent with previously reported analytical data.[4]

(*S*)-2-((*S*)-2-amino-3-methylbutanamido)-*N*-(4-(hydroxymethyl)phenyl)-5-ureidopentanamide

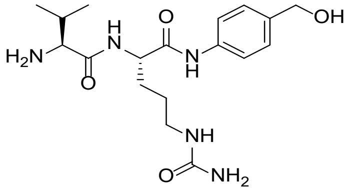

Piperidine (0.67 mL, 6.8 mmol) was added to a solution of (9*H*-fluoren-9-yl)methyl ((*S*)-1-(((*S*)-1-((4-(hydroxymethyl)phenyl)amino)-1-oxo-5-ureidopentan-2-yl)amino)-3-methyl-1-oxobutan-2-yl)carbamate (3.1 g, 3.35 mmol) in DMF (10 mL) and the reaction was stirred at r.t. for 3 h. The reaction

mixture was diluted with water (500 mL) and filtered by vacuum. The filtrand was washed with water (3 x 200 mL) and the washings were combined and concentrated under reduced pressure. The residue was purified by reverse phase flash column chromatography (C18 200 g, 0-30% 10 mM aqueous ammonium carbonate/Acetonitrile). The appropriate fractions were combined and concentrated under reduced pressure and dried under vacuum to afford (*S*)-2-((*S*)-2-amino-3-methylbutanamido)-*N*-(4-(hydroxymethyl)phenyl)-5-ureidopentanamide (1.28 g, 3.4 mmol, quant. yield) as a white solid.

^{1}H NMR (400 MHz, CD_3SOCD_3, 303 K) δ (ppm) = 10.01 (s, 1H), 8.10 (br s, 1H), 7.61 - 7.48 (m, 2H), 7.24 (d, *J* = 8.6 Hz, 2H), 5.97 (br t, *J* = 5.6 Hz, 1H), 5.39 (s, 2H), 5.08 (br s, 1H), 4.54 - 4.37 (m, 3H), 3.09 - 2.90 (m, 3H), 2.00 - 1.82 (m, 1H), 1.81 - 1.54 (m, 4H), 1.54 - 1.31 (m, 2H), 0.89 (d, *J* = 6.8 Hz, 3H), 0.79 (d, *J* = 6.8 Hz, 3H) **^{13}C NMR** (101 MHz, CD_3SOCD_3, 303 K) δ (ppm) = 175.0, 171.0, 159.3, 138.0, 137.9, 127.4, 119.4, 63.1, 60.2, 53.0, 39.2, 31.8, 30.6, 27.2, 20.0, 17.4 **LCMS** HpH, rt = 0.52 mins, $[M+H]^+$ = 380.2, **IR**(ATIR, cm $^{-1}$): 3297, 2939, 1630, 1532, 1412, 1390, 1342, 1252, 1205, 1181, 1131, 1056, 986, 685, 508.

Consistent with previously reported analytical data.[4]

4-(((*S*)-1-(((*S*)-1-((4-(Hydroxymethyl)phenyl)amino)-1-oxo-5-ureidopentan-2-yl)amino)-3-methyl-1-oxobutan-2-yl)amino)-4-oxobutanoic acid

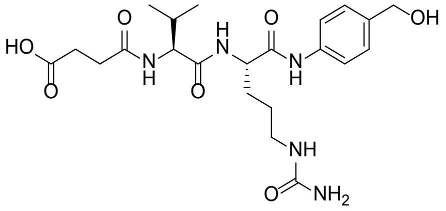

Succinic anhydride **23** (200 mg, 2.0 mmol) was added to a solution of (S)-2-((S)-2-amino-3-methylbutanamido)-*N*-(4-(hydroxymethyl)phenyl)-5-ureidopentanamide (750 mg, 2.0 mmol) and triethylamine (0.41 mL, 2.9 mmol) in DMF (15 mL) and the reaction was stirred for 1 h at r.t.. The reaction mixture was concentrated under reduced pressure and then was purified by reverse phase flash column chromatography (C18 120 g, 0-60% 0.1% formic acid in water/0.1% formic acid in acetonitrile). The appropriate fractions were combined and concentrated in vacuo to afford 4-(((*S*)-1-(((*S*)-1-((4-(hydroxymethyl)phenyl)amino)-1-oxo-5-ureidopentan-2-yl)amino)-3-methyl-1-oxobutan-2-yl)amino)-4-oxobutanoic acid (854 mg, 1.781 mmol, 90 % yield), as a white solid.

^{1}H NMR (400 MHz, CD_3SOCD_3, 303 K) δ (ppm) = 12.08 (br s, 1H), 9.78 (s, 1H), 8.05 (d, *J* = 7.6 Hz, 1H), 7.92 (d, *J* = 8.3 Hz, 1H), 7.62 - 7.52 (m, 2H), 7.29 - 7.19 (m, 2H), 6.02 (br t, *J* = 5.4 Hz, 1H), 5.43 (br s, 2H), 5.07 (t, *J* = 5.7 Hz, 1H), 4.50 - 4.33 (m, 3H), 4.19 (dd, *J* = 6.6, 8.3 Hz, 1H), 3.07 - 2.91 (m, 2H), 2.49 - 2.40 (m, 4H), 2.07 - 1.93 (m, 1H), 1.78 - 1.54 (m, 2H), 1.53 - 1.31 (m, 2H), 0.88 (br d, *J* = 6.8 Hz, 3H), 0.85 (br d, *J* = 6.8 Hz, 3H) **^{13}C NMR** (101 MHz, CD_3SOCD_3, 303 K) δ (ppm) = 174.5, 172.1, 171.6, 170.8, 159.4, 138.0, 137.9, 127.4, 119.3, 63.1, 58.4, 53.7, 39.1, 30.8, 30.5, 29.9, 29.7, 27.3, 19.7, 18.5, **LCMS** formic, rt = 0.55 mins, $[M+H]^+$ = 480.2, **IR**(ATIR, cm^{-1}): 3271, 2955, 1710, 1631, 1528, 1391, 1243, 1009, 707. **HRMS (ESI)** molecular formula($C_{22}H_{33}N_5O_7$) *m/z* found $[M+H]^+$ 480.2465, $C_{22}H_{34}N_5O_7^+$ requires 480.2453

(*rac*-(1*R*,8*S*,9s)-bicyclo[6.1.0]non-4-yn-9-yl)methyl ((*S*)-3-methyl-1-(((*S*)-1-((4-((((4-nitrophenoxy)carbonyl)oxy)methyl)phenyl)amino)-1-oxo-5-ureidopentan-2-yl)amino)-1-oxobutan-2-yl)carbamate 5

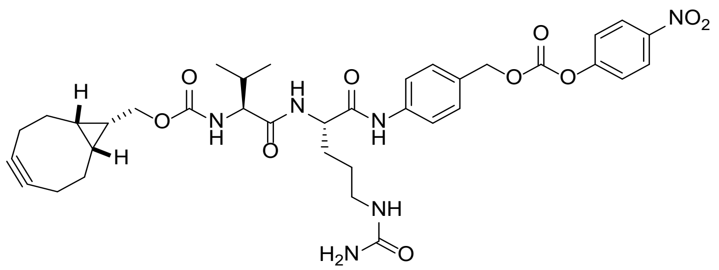

((1*R*,8*S*,9s)-Bicyclo[6.1.0]non-4-yn-9-yl)methyl (2,5-dioxopyrrolidin-1-yl) carbonate (42 mg, 0.14 mmol) was added to a solution of (*S*)-2-((*S*)-2-amino-3-methylbutanamido)-*N*-(4-(hydroxymethyl)phenyl)-5-ureidopentanamide (50 mg, 0.13 mmol) in DMF (1 mL) and the reaction was stirred for 0.5 h at r.t.. Bis(4-nitrophenyl) carbonate (0.12 g, 0.40 mmol) and DIPEA (68 mg, 92 µL, 0.53 mmol) were added and the reaction was stirred for a further 3 h. The reaction mixture was directly purified by Prep-HPLC (EZPrep, Formic, 30-85% modified acetonitrile/modified water). The appropriate fractions were combined and concentrated under reduced pressure. This was then dried under vacuum to afford (*rac*-(1*R*,8*S*,9s)-bicyclo[6.1.0]non-4-yn-9-yl)methyl ((*S*)-3-methyl-1-(((*S*)-1-((4-((((4-nitrophenoxy)carbonyl)oxy)methyl)phenyl)amino)-1-oxo-5-ureidopentan-2-yl)amino)-1-oxobutan-2-yl)carbamate **5** (33 mg, 46 µmol, 35 %) as a colourless solid.

^{1}H NMR (400 MHz, CD_3SOCD_3, 303 K) δ (ppm) = 10.10 (s, 1H), 8.35 - 8.28 (m, 2H), 8.04 (br d, *J* = 7.6 Hz, 1H), 7.69 - 7.61 (m, 2H), 7.61 - 7.53 (m, 2H), 7.46 - 7.38 (m, 2H), 7.11 (br d, *J* = 8.6 Hz, 1H), 5.98 (s, 1H), 5.41 (s, 2H), 5.25 (s, 2H), 4.52 - 4.38 (m, 1H), 4.07 (d, *J* = 8.1 Hz, 2H), 3.90 (br t, *J* = 7.7 Hz, 1H), 3.10 - 2.91 (m, 2H), 2.26 - 2.09 (m, 6H), 1.99 (qd, *J* = 13.4, 6.7 Hz, 1H), 1.76 - 1.67 (m, 1H), 1.65 - 1.34 (m, 5H), 1.34 - 1.21 (m, 1H), 0.87 (br dd, *J* = 6.8, 14.4 Hz, 8H). **^{13}C NMR** (101 MHz, CD_3SOCD_3, 303 K) δ (ppm) = 171.7, 164.7, 159.3, 156.9, 155.8, 152.4, 145.6, 139.9, 139.8, 129.9, 127.4, 125.8, 123.1, 119.5, 119.3, 99.4, 63.1, 62.1, 60.7, 53.4, 39.1, 30.8, 30.1, 29.0, 27.2, 21.3, 20.1, 19.7, 18.6, 18.1. Complex NMR as sample partially degraded. **LCMS**: formic, RT = 1.15 min, $[M+H]^+$ = 721.2, 95% purity by UV-Vis. **IR** (cm^{-1}, ATIR): 3285, 2924, 1761, 1645, 1520, 1346, 1209, 1023. **HRMS (ESI)** molecular formula ($C_{36}H_{44}N_6O_{10}$) *m/z* found $[M+H]^+$ 721.3188, $C_{36}H_{45}N_6O_{10}^+$ requires 721.3192.

***N*1-(2-(2-(2-(2-Azidoethoxy)ethoxy)ethoxy)ethyl)-*N*4-((*S*)-1-(((*S*)-1-((4-(hydroxymethyl)phenyl)amino)-1-oxo-5-ureidopentan-2-yl)amino)-3-methyl-1-xobutan-2-yl)succinamide**

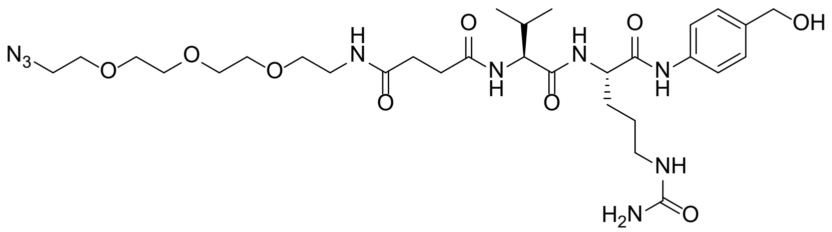

HATU (104 mg, 0.274 mmol) was added to a solution of 4-(((*S*)-1-(((*S*)-1-((4-(hydroxymethyl)phenyl)amino)-1-oxo-5-ureidopentan-2-yl)amino)-3-methyl-1-oxobutan-2-yl)amino)-4-oxobutanoic acid (75 mg, 0.156 mmol) and DIPEA (0.055 mL, 0.31 mmol) in *N,N*-Dimethylformamide (DMF) (1 mL). The reaction was stirred for 10 minutes before 2-(2-(2-(2-azidoethoxy)ethoxy)ethoxy)ethan-1-amine **26** (51.2 mg, 0.24 mmol) was added and the reaction was stirred at r.t. for 1.5 h. The reaction mixture was directly purified by prep-HPLC (formic modified, 5-55% 0.1% formic acid modified acetonitrile/0.1% formic acid modified water) the appropriate fractions

were combined and concentrated under reduced pressure and dried under vacuum to afford *N*1-(2-(2-(2-(2-azidoethoxy)ethoxy)ethoxy)ethyl)-*N*4-((*S*)-1-(((*S*)-1-((4-(hydroxymethyl)phenyl)amino)-1-oxo-5-ureidopentan-2-yl)amino)-3-methyl-1-oxobutan-2-yl)succinimide (72 mg, 0.11 mmol, 68 % yield) as yellow solid.

^{1}H NMR (400 MHz, CD_3SOCD_3, 303 K) δ (ppm) = 9.54 (s, 1H), 8.08 (d, *J* = 7.8 Hz, 1H), 8.01 (d, *J* = 7.6 Hz, 1H), 7.95 (t, *J* = 5.5 Hz, 1H), 7.60 (d, *J* = 8.4 Hz, 2H), 7.24 (d, *J* = 8.4 Hz, 2H), 6.14 - 5.89 (m, 1H), 4.44 (s, 2H), 4.38 - 4.31 (m, 1H), 4.17 - 4.09 (m, 1H), 3.62 - 3.58 (m, 2H), 3.58 - 3.44 (m, 8H), 3.41 - 3.29 (m, 4H), 3.15 (q, *J* = 6.0 Hz, 2H), 3.07 - 2.92 (m, 2H), 2.50 - 2.31 (m, 4H), 2.07 (qd, *J* = 6.8, 13.0 Hz, 1H), 1.84 - 1.75 (m, 1H), 1.65 (dtd, *J* = 13.9, 9.4, 4.9 Hz, 1H), 1.53 - 1.32 (m, 2H), 0.91 (br d, *J* = 6.8 Hz, 3H), 0.88 (br d, *J* = 6.8 Hz, 3H). **^{13}C NMR** (101 MHz, CD_3SOCD_3, 303 K) δ (ppm) = 173.3, 172.3, 171.6, 170.8, 159.3, 137.9, 137.9, 127.3, 119.3, 70.25, 70.18, 70.16, 70.0, 69.7, 69.5, 63.1, 58.9, 53.8, 50.5, 39.2, 39.1, 31.15, 31.07, 30.3, 29.4, 27.4, 19.6, 18.3. **IR** (ATIR, cm^{-1}): 3266 (br.), 2870, 2103, 1628, 1536, 1413, 1384, 1298, 1104, 1011. 842, 1011, 842, 712, 557, 490. **LCMS**: high pH, rt = 0.68 mins, $[M+H]^+$ = 680.3. **HRMS (ESI)** molecular formula ($C_{30}H_{50}N_9O_9$) *m/z* found $[M+H]^+$ 680.3721, $C_{30}H_{51}N_9O_9^+$ requires 680.3726.

4-((18*S*,21*S*)-1-Azido-18-isopropyl-13,16,19-trioxo-21-(3-ureidopropyl)-3,6,9-trioxa-2,17,20-triazadocosan-22-amido)benzyl (4-nitrophenyl) carbonate 8

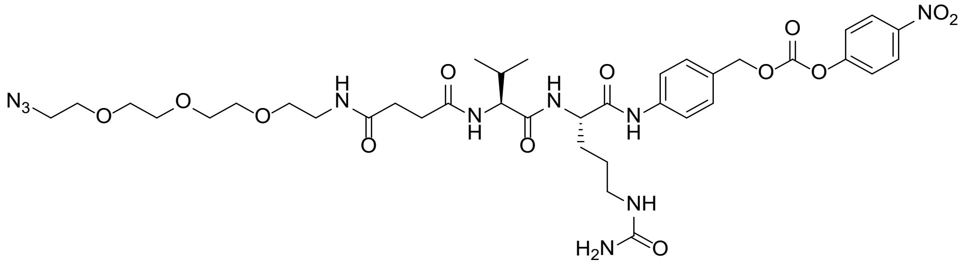

Bis(4-nitrophenyl) carbonate (90 mg, 0.30 mmol) was added to a solution of *N*1-(2-(2-(2-(2-azidoethoxy)ethoxy)ethoxy)ethyl)-*N*4-((*S*)-1-(((*S*)-1-((4-(hydroxymethyl)phenyl)amino)-1-oxo-5-ureidopentan-2-yl)amino)-3-methyl-1-oxobutan-2-yl)succinimide (67 mg, 99 µmol) and DIPEA (51 mg, 69 µL, 0.39 mmol) in DMF (1 mL) and the reaction was stirred at r.t. for 2 h. The reaction mixture was directly purified by Prep-HPLC (EZPrep, 40-95% 0.1% formic acid in MeCN/0.1% formic acid in water) and the appropriate fractions were combined and concentrated under a stream of nitrogen to afford 4-((18S,21S)-1-azido-18-isopropyl-13,16,19-trioxo-21-(3-ureidopropyl)-3,6,9-trioxa-12,17,20-triazadocosan-22-amido)benzyl (4-nitrophenyl) carbonate **8** (68 mg, 80 µmol, 82 %) as a white solid.

^{1}H NMR (400 MHz, CD_3SOCD_3, 303 K) δ (ppm) = 9.71 (s, 1H), 8.32 (d, *J* = 8.2 Hz, 2H), 8.11 (d, *J* = 7.8 Hz, 1H), 8.02 (d, *J* = 7.6 Hz, 1H), 7.95 (t, *J* = 5.6 Hz, 1H), 7.74 - 7.68 (m, 2H), 7.61 - 7.54 (m, 2H), 7.42 (d, *J* = 8.6 Hz, 2H), 5.98 (t, *J* = 5.7 Hz, 1H), 5.40 (s, 2H), 5.25 (s, 2H), 4.44 - 4.32 (m, 1H), 4.14 (dd, *J* = 7.8, 5.9 Hz, 1H), 3.61 - 3.57 (m, 2H), 3.57 - 3.48 (m, 6H), 3.48 - 3.43 (m, 2H), 3.40 - 3.37 (m, 2H), 3.37 - 3.29 (m, 5H), 3.16 (q, *J* = 6.0 Hz, 2H), 3.08 - 2.91 (m, 2H), 2.50 - 2.34 (m, 4H), 2.12 - 2.02 (m, 1H), 1.85 - 1.61 (m, 2H), 1.54 - 1.33 (m, 2H), 0.91 (br d, *J* = 6.8 Hz, 3H), 0.88 (br d, *J* = 6.8 Hz, 3H). **^{13}C NMR** (101 MHz, CD_3SOCD_3, 303 K) δ (ppm) = 173.3, 172.3, 171.7, 171.2, 159.3, 155.8, 152.4, 145.6, 139.8, 129.9, 129.8, 125.9, 123.0, 119.5, 70.7, 70.25, 70.18, 70.16, 70.0, 69.7, 69.5, 58.8, 53.9, 50.5, 39.13, 39.08, 31.2, 31.1, 30.4, 29.3, 27.5, 19.6, 18.4. **LCMS**: Formic, RT = 0.98 min, $[M+H]^+$ = 845.3, 97% purity by UV-Vis. **IR** (ATIR, cm^{-1}): 3269, 3075, 2922, 2870, 2103, 1753, 1630, 1523, 1346, 1275, 1211, 1106. **HRMS**: molecular formula ($C_{37}H_{52}N_{10}O_{13}$) *m/z* found $[M+H]^+$ 845.3778, $C_{37}H_{53}N_{10}O_{13}^+$ requires 845.3788.

rac-((1_R_,8_S_,9_s_)-Bicyclo[6.1.0]non-4-yn-9-yl)methyl ((6_S_,9_S_)-1-amino-6-((4-(hydroxymethyl)phenyl)carbamoyl)-9-isopropyl-1,8,11,14-tetraoxo-18,21-dioxa-2,7,10,15-tetraazatricosan-23-yl)carbamate

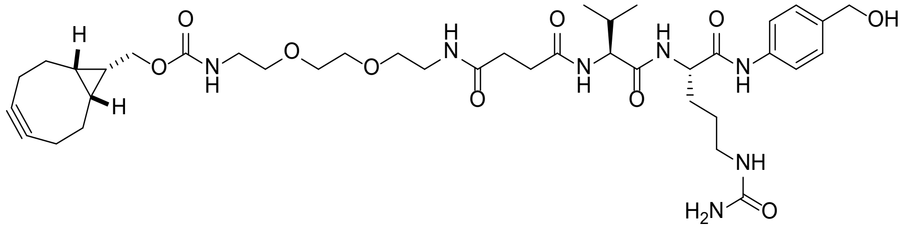

HATU (52 mg, 0.14 mmol) was added to a solution of 4-(((*S*)-1-(((*S*)-1-((4-(hydroxymethyl)phenyl)amino)-1-oxo-5-ureidopentan-2-yl)amino)-3-methyl-1-oxobutan-2-yl)amino)-4-oxobutanoic acid (50 mg, 0.10 mmol) and DIPEA (55 µL, 0.32 mmol) in DMF (0.5 mL). This was then stirred at r.t. for 10 mins before a solution of (*rac*-(1*R*,8*S*,9*s*)-bicyclo[6.1.0]non-4-yn-9-yl)methyl (2-(2-(2-aminoethoxy)ethoxy)ethyl)carbamate (41 mg, 0.13 mmol) in DMF(0.5 mL) was added and the reaction was stirred for a further 1.5 h. The reaction mixture was directly purified by prep-HPLC (ACCQ Prep, 17.5 min, 10-55% 0.1% formic acid modified acetonitrile/0.1% formic acid modified water) (**NOTE**: The UV detector was changed to 300 nm to prevent degradation; however, compound did not absorb at this wavelength and was found by LCMS). The appropriate fractions were concentrated under reduced and was dried under vacuum to afford ((1*R*,8*S*,9*s*)-bicyclo[6.1.0]non-4-yn-9-yl)methyl ((6*S*,9*S*)-1-amino-6-((4-(hydroxymethyl)phenyl)carbamoyl)-9-isopropyl-1,8,11,14-tetraoxo-18,21-dioxa-2,7,10,15-tetraazatricosan-23-yl)carbamate (68 mg, 0.087 mmol, 83 % yield, 90% purity) as a white solid.

^{1}H NMR (400 MHz, CD_3SOCD_3, 303 K) δ (ppm) = 9.55 (s, 1H), 8.08 (d, *J* = 8.1 Hz, 1H), 8.01 (d, *J* = 7.8 Hz, 1H), 7.95 (t, *J* = 5.5 Hz, 1H), 7.59 (d, *J* = 8.6 Hz, 2H), 7.24 (d, *J* = 8.8 Hz, 2H), 7.07 (br t, *J* = 5.5 Hz, 1H), 6.09 - 5.83 (m, 1H), 5.75 - 4.80 (m, 2H), 4.44 (s, 2H), 4.38 - 4.29 (m, 1H), 4.13 (dd, *J* = 7.7, 6.0 Hz, 1H), 4.04 (br d, *J* = 7.8 Hz, 2H), 3.51 - 3.42 (m, 4H), 3.42 - 3.29 (m, 4H), 3.20 - 3.07 (m, 4H), 3.07 - 2.90 (m, 2H), 2.49 - 2.29 (m, 4H), 2.28 - 2.02 (m, 6H), 1.86 - 1.73 (m, 1H), 1.65 (dtd, *J* = 4.8, 9.5, 13.9 Hz, 1H), 1.59 - 1.33 (m, 4H), 1.33 - 1.18 (m, 1H), 0.97 - 0.81 (m, 8H). One absent proton corresponding to BCN, possibly lost due to rounding. **^{13}C NMR** (101 MHz, CD_3SOCD_3, 303 K) δ (ppm) = 173.2, 172.3, 171.6, 170.8, 159.3, 156.9, 137.9, 137.9, 127.3, 119.3, 99.5, 70.0, 69.9, 69.6, 69.5, 63.1, 61.8, 58.8, 53.8, 40.6, 39.2, 39.1, 31.14, 31.06, 30.4, 29.4, 29.1, 27.4, 21.3, 20.0, 19.6, 18.3, 18.1. **IR**(ATIR, cm^{-1}): 3268, 2919, 1627, 1533, 1250, 1100, 842, 557. **LCMS**: formic, rt = 0.84 mins, $[M+H]^+$ = 786.4. **HRMS (ESI)** molecular formula ($C_{39}H_{59}N_7O_{10}$) *m/z* found $[M+H]^+$ 786.4399, $C_{39}H_{60}N_7O_{10}^+$ requires 786.4396

***rac*-((1*R*,8*S*,9*s*)-Bicyclo[6.1.0]non-4-yn-9-yl)methyl ((6*S*,9*S*)-1-amino-9-isopropyl-6-((4-((((4-nitrophenoxy)carbonyl)oxy)methyl)phenyl)carbamoyl)-1,8,11,14-tetraoxo-18,21-dioxa-2,7,10,15-tetraazatricosan-23-yl)carbamate 14**

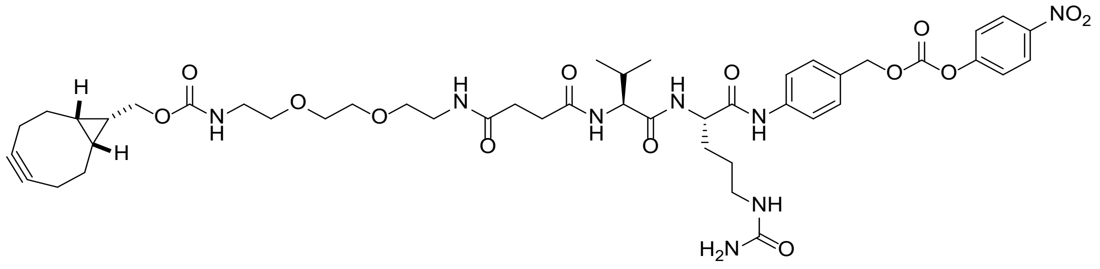

DIPEA (21 µL, 0.12 mmol) was added to a solution of ((1*R*,8*S*,9*s*)-bicyclo[6.1.0]non-4-yn-9-yl)methyl ((6*S*,9*S*)-1-amino-6-((4-(hydroxymethyl)phenyl)carbamoyl)-9-isopropyl-1,8,11,14-tetraoxo-18,21-dioxa-2,7,10,15-tetraazatricosan-23-yl)carbamate (24 mg, 0.031 mmol) and bis(4-nitrophenyl) carbonate (28 mg, 0.092 mmol) in DMF (0.5 mL) and the reaction was stirred at r.t. for 1 h. The reaction mixture was directly purified by prep-HPLC (ACCQ Prep, 15-85% 0.1% formic acid modified acetonitrile/0.1% formic acid modified water) and the appropriate fractions were concentrated under reduced pressure. The residue was transferred in methanol and concentrated under a stream of nitrogen. This was then dried in vacuum to afford (*rac*-(1R,8*S*,9*s*)-bicyclo[6.1.0]non-4-yn-9-yl)methyl ((6*S*,9*S*)-1-amino-9-isopropyl-6-((4-((((4-nitrophenoxy)carbonyl)oxy)methyl)phenyl)carbamoyl)-1,8,11,14-tetraoxo-18,21-dioxa-2,7,10,15-tetraazatricosan-23-yl)carbamate **14** (17 mg, 0.018 mmol, 59 % yield) as a white solid.

^{1}H NMR (400 MHz, CD_3SOCD_3, 303 K) δ (ppm) = 9.72 (br s, 1H), 8.32 (br d, *J* = 8.8 Hz, 2H), 8.11 (br d, *J* = 7.8 Hz, 1H), 8.05 - 7.98 (m, 1H), 7.95 (br s, 1H), 7.71 (br d, *J* = 8.3 Hz, 2H), 7.64 - 7.51 (m, 2H), 7.51 - 7.35 (m, 2H), 7.06 (br s, 1H), 5.97 (br s, 1H), 5.39 (br s, 2H), 5.32 - 5.19 (m, 2H), 4.35 (br s, 1H), 4.14 (br t, *J* = 6.4 Hz, 1H), 4.08 - 3.96 (m, 2H), 3.55 - 3.26 (m, 1H), 3.20 - 3.08 (m, 4H), 3.07 - 2.90 (m, 2H), 2.48 - 2.32 (m, 4H), 2.31 - 2.00 (m, 7H), 1.90 - 1.72 (m, 1H), 1.72 - 1.58 (m, 1H), 1.58 - 1.35 (m, 1H), 1.34 - 1.21 (m, 1H), 0.99 - 0.78 (m, 9H). **^{13}C NMR** (101 MHz, CD_3SOCD_3, 303 K) δ (ppm) = 173.2, 172.3, 171.7, 171.2, 159.3, 156.9, 155.8, 152.4, 145.6, 139.8, 129.9, 129.8, 125.9, 123.1, 119.5, 99.5, 70.7, 70.0, 69.9, 69.6, 69.5, 61.8, 58.8, 53.8, 40.6, 39.1, 39.1, 31.14, 31.07, 30.4, 29.3, 29.1, 27.4, 21.3, 20.0, 19.6, 18.4, 18.1. **LCMS**: formic, rt = 1.08 mins, MH+ = 951.5, 98% pure by UV. **IR** (ATIR, cm^{-1}): 3273, 2920, 1757, 1626, 1524, 1347, 1257, 1211, 1110, 864, 715, 665, 554, 497. **HRMS**: formic, molecular formula ($C_{46}H_{62}N_8O_{14}$) *m/z* found $[M+H]^+$ 951.4460, $C_{46}H_{63}N_8O_{14}^+$ requires 951.4458.

***N*1-(14-Amino-3,6,9,12-tetraoxatetradecyl)-*N*4-((*S*)-1-(((*S*)-1-((4-(hydroxymethyl)phenyl)amino)-1-oxo-5-ureidopentan-2-yl)amino)-3-methyl-1-oxobutan-2-yl)succinamide**

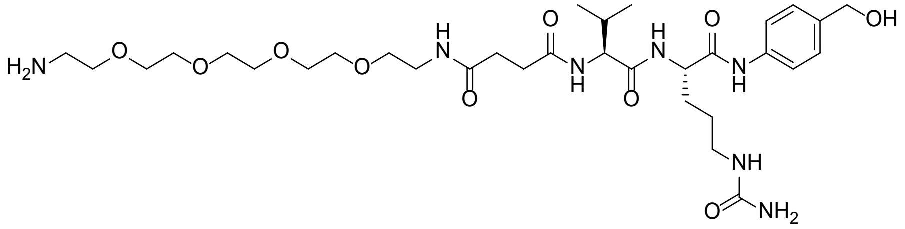

DIPEA (43 mg, 58 µL, 0.33 mmol) was added to a solution of 4-(((*S*)-1-(((*S*)-1-((4-(hydroxymethyl)phenyl)amino)-1-oxo-5-ureidopentan-2-yl)amino)-3-methyl-1-oxobutan-2-yl)amino)-4-oxobutanoic acid (80 mg, 0.17 mmol) and HATU (95 mg, 0.25 mmol) in DMF (1 mL) and the reaction was stirred at r.t. for 30 mins. This was then added dropwise over 30 mins to 3,6,9,12-tetraoxatetradecane-1,14-diamine (390 mg, 1.65 mmol) and the reaction was stirred for a further h at r.t.. The reaction mixture was directly purified by Prep-HPLC (EZPrep, 0-40% acetonitrile/10 mM aq. ammonium carbonate solution) and the appropriate fractions were combined and concentrated under reduced pressure. This was then dried in vacuum to afford *N*1-(14-amino-3,6,9,12-tetraoxatetradecyl)-*N*4-((*S*)-1-(((*S*)-1-((4-(hydroxymethyl)phenyl)amino)-1-oxo-5-ureidopentan-2-yl)amino)-3-methyl-1-oxobutan-2-yl)succinamide (20 mg, 29 µmol, 17 %) as a white solid.

^{1}H NMR (400 MHz, CD_3SOCD_3, 303 K) δ (ppm) = 9.71 - 9.62 (m, 1H), 8.58 - 8.29 (m, 1H), 8.16 (br d, *J* = 7.3 Hz, 1H), 8.06 (br d, *J* = 7.6 Hz, 1H), 7.99 (br s, 1H), 7.66 - 7.52 (m, 2H), 7.29 - 7.16 (m, 2H), 6.23 - 6.03 (m, 1H), 5.44 (br s, 2H), 4.43 (s, 2H), 4.33 (br dd, *J* = 13.0, 9.0 Hz, 2H), 4.12 (br dd, *J* = 6.2, 7.5 Hz, 2H), 3.52 (br d, *J* = 10.5 Hz, 9H), 3.39 - 3.29 (m, 2H), 3.22 - 3.09 (m, 2H), 2.97 (tt, *J* = 6.5, 13.6 Hz, 2H), 2.85 (br s, 2H), 2.48 - 2.33 (m, 4H), 2.06 (br dd, *J* = 13.0, 6.6 Hz, 1H), 1.83 - 1.72 (m, 1H), 1.72 - 1.59 (m, 1H), 1.53 - 1.29 (m, 2H), 0.89 (br d, *J* = 6.8 Hz, 3H), 0.87 (br d, *J* = 6.8 Hz, 3H). **^{13}C NMR** (101 MHz, CD_3SOCD_3, 303 K) δ (ppm) = 173.2, 172.3, 171.7, 170.9, 159.4, 138.0, 137.9, 127.3, 119.3, 70.2, 70.1, 70.1, 70.0, 69.5, 63.1, 58.9, 53.8, 39.1, 31.2, 31.1, 30.4, 29.4, 27.4, 19.7, 18.4. 5 signals not observed, suspected to be from PEG signals overlapping. **LCMS**: HpH, RT = 0.60 mins, $[M+H]^+$ = 698.3, 100 % purity by UV-Vis **IR** (ATIR, cm^{-1}): 3454, 3274, 2922, 2868, 1625, 1538, 1345, 1095. **HRMS**: formic, molecular formula ($C_{32}H_{55}N_7O_{10}$) *m/z* found $[M+H]^+$ 698.4082 , $C_{32}H_{56}N_7O_{10}^+$requires 698.4083.

(*rac*-(1*R*,8*S*,9*s*)-Bicyclo[6.1.0]non-4-yn-9-yl)methyl ((6*S*,9*S*)-1-amino-9-isopropyl-6-((4-((((4-nitrophenoxy)carbonyl)oxy)methyl)phenyl)carbamoyl)-1,8,11,14-tetraoxo-18,21,24,27-tetraoxa-2,7,10,15-tetraazanonacosan-29-yl)carbamate 15

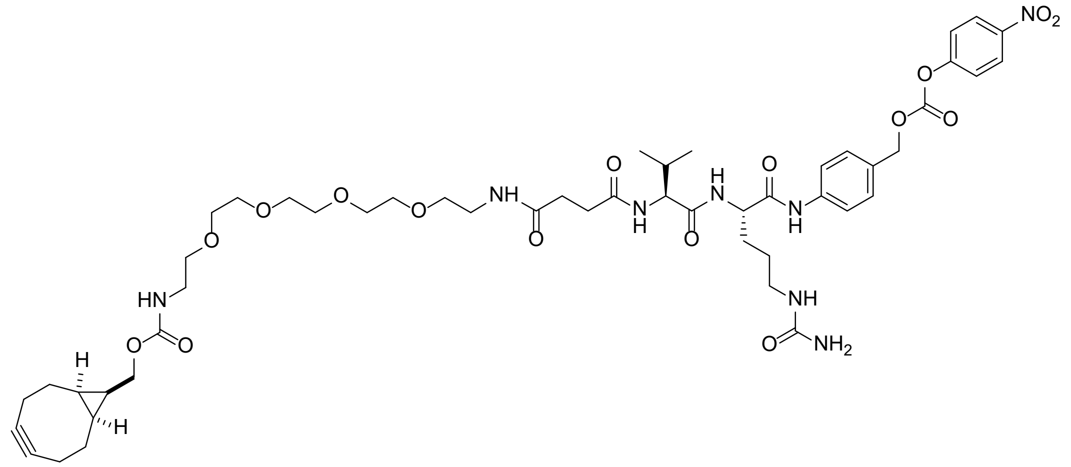

(*rac*-(1*R*,8*S*,9s)-Bicyclo[6.1.0]non-4-yn-9-yl)methyl (2,5-dioxopyrrolidin-1-yl) carbonate (14 mg, 48 µmol) was added to a solution of *N*1-(14-amino-3,6,9,12-tetraoxatetradecyl)-*N*4-((*S*)-1-(((*S*)-1-((4-(hydroxymethyl)phenyl)amino)-1-oxo-5-ureidopentan-2-yl)amino)-3-methyl-1-oxobutan-2-yl)succinamide (31 mg, 44 µmol) in DMF (0.5 mL) and the reaction was stirred at r.t. for 1.5 h. To this, bis(4-nitrophenyl) carbonate (41 mg, 0.13 mmol) and DIPEA (23 mg, 31 µL, 0.18 mmol) was added and the reaction was stirred at r.t. for 4 h. The reaction mixture was directly purified by prep-HPLC (formic modified, 5-70% acetonitrile/water) and the appropriate fractions were combined and concentrated under reduced pressure. This was then dried in vacuum to afford (rac-(1*R*,8*S*,9s)-bicyclo[6.1.0]non-4-yn-9-yl)methyl ((6*S*,9*S*)-1-amino-9-isopropyl-6-((4-((((4-nitrophenoxy)carbonyl)oxy)methyl)phenyl)-carbamoyl)-1,8,11,14-tetraoxo-18,21,24,27-tetraoxa-2,7,10,15-tetraazanona-cosan-29-yl)carbamate, **15** (18 mg, 17 µmol, 39 %) as a colourless solid.

^{1}H NMR (600 MHz, CD_3SOCD_3, 300 K) δ (ppm) = 9.72 (s, 1H), 8.33 - 8.30 (m, 1H), 8.12 (br d, *J* = 7.7 Hz, 1H), 8.03 (br d, *J* = 7.7 Hz, 1H), 7.97 (br t, *J* = 5.5 Hz, 1H), 7.71 (d, *J* = 8.6 Hz, 2H), 7.59 - 7.55 (m, 1H), 7.42 (d, *J* = 8.4 Hz, 2H), 7.07 (br t, *J* = 5.1 Hz, 1H), 6.00 - 5.96 (m, 1H), 5.40 (br s, 2H), 5.25 (s, 2H), 4.37 - 4.32 (m, 1H), 4.15 - 4.12 (m, 1H), 4.03 (br d, *J* = 8.1 Hz, 2H), 3.49 - 3.30 (m, 22H), 3.17 - 3.10 (m, 4H), 3.02 (td, *J* = 12.9, 6.4 Hz, 1H), 2.96 (td, *J* = 12.9, 6.4 Hz, 1H), 2.49 - 2.34 (m, 4H), 2.26 - 2.19 (m, 2H), 2.17 - 2.11 (m, 4H), 2.09 - 2.03 (m, 1H), 1.82 - 1.76 (m, 1H), 1.66 (dtd, *J* = 13.9, 9.4, 4,8 Hz, 1H), 1.57 - 1.44 (m, 3H), 1.42 - 1.34 (m, 1H), 1.30 - 1.23 (m, 1H), 0.92 - 0.83 (m, 8H). **^{13}C NMR** (151 MHz, CD_3SOCD_3, 300 K) δ (ppm) = 173.3, 172.2, 171.7, 171.2, 159.3, 156.9, 155.8, 152.4, 145.6, 139.8, 129.9, 129.8, 125.9, 123.1, 119.5, 99.5, 70.7, 70.23, 70.22, 70.18, 70.1, 70.0, 69.6, 69.5, 61.8, 58.8, 53.8, 40.5, 39.1, 31.1, 31.0, 30.4, 29.3, 29.1, 27.5, 21.3, 20.0, 19.6, 18.4, 18.1. 2 PEG signals missing, suspected to be overlapping. **LCMS**: HpH, RT = 1.11 min, $[M+H]^+$ = 1039.5, 95% purity by UV-Vis. **IR**(ATIR, cm-1): 3273, 2921, 2870, 1757, 1705, 1630, 1524, 1347, 1524, 1347, 1210, 1096. **HRMS**: molecular formula ($C_{50}H_{70}N_8O_{16}$) *m/z* found $[M+H]^+$ 1039.4998, $C_{50}H_{71}N_8O_{16}^+$ requires 1039.4983

***N*1-(20-Amino-3,6,9,12,15,18-hexaoxaicosyl)-*N*4-((S)-1-(((*S*)-1-((4-(hydroxymethyl)phenyl)amino)-1-oxo-5-ureidopentan-2-yl)amino)-3-methyl-1-oxobutan-2-yl)succinamide**

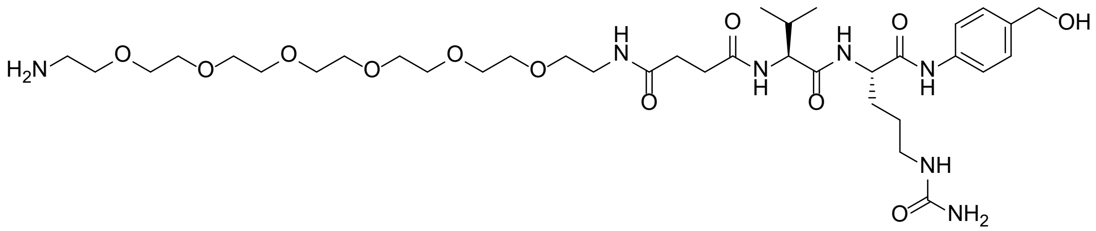

DIPEA (80.9 mg, 109 µL, 626 µmol) was added to a solution of 4-(((*S*)-1-(((*S*)-1-((4-(hydroxymethyl)phenyl)amino)-1-oxo-5-ureidopentan-2-yl)amino)-3-methyl-1-oxobutan-2-yl)amino)-4-oxobutanoic acid (150 mg, 313 µmol) and HATU (143 mg, 375 µmol) in DMF (1 mL) and the reaction was stirred for 20 mins at r.t.. After this time the reaction mixture was added dropwise over 30 mins to 3,6,9,12,15,18-hexaoxaicosane-1,20-diamine (812 mg, 2.50 mmol) and the reaction was stirred for a further h at r.t.. The reaction mixture was directly purified by Prep-HPLC (EZPrep, 0-40% acetonitrile/10 mM aq. ammonium carbonate solution) and the appropriate fractions were combined and concentrated under reduced pressure. This was then dried under vacuum to afford *N*1-(20-amino-3,6,9,12,15,18-hexaoxaicosyl)-*N*4-((*S*)-1-(((*S*)-1-((4-(hydroxymethyl)phenyl)amino)-1-oxo-5-ureidopentan-2-yl)amino)-3-methyl-1-oxobutan-2-yl)succinimide (57 mg, 73 µmol, 23 %) as a pale yellow solid.

^{1}H NMR (400 MHz, CD_3SOCD_3, 303 K) δ (ppm) = 9.55 (s, 1H), 8.09 (d, *J* = 7.8 Hz, 1H), 8.02 (d, *J* = 7.6 Hz, 1H), 7.96 (t, *J* = 5.6 Hz, 1H), 7.59 (d, *J* = 8.6 Hz, 2H), 7.23 (d, *J* = 8.6 Hz, 2H), 5.97 (br t, *J* = 5.7 Hz, 1H), 5.38 (s, 2H), 4.43 (s, 2H), 4.38 - 4.29 (m, 1H), 4.13 (dd, *J* = 7.6, 5.9 Hz, 1H), 3.57 - 3.42 (m, 30H), 3.41 - 3.21 (m, 23H), 3.21 - 3.10 (m, 8H), 3.10 - 2.90 (m, 4H), 2.65 (t, *J* = 5.9 Hz, 2H), 2.49 - 2.31 (m, 4H), 2.07 (br dd, *J* =13.1, 6.7 Hz, 1H), 1.84 - 1.73 (m, 1H), 1.65 (br dd, *J* = 9,4, 4.5 Hz, 1H), 1.54 - 1.42 (m, 1H), 1.42 - 1.30 (m, 1H), 0.89 (dd, *J* = 10.0, 6.7 Hz, 6H) Excessive number of protons between 3.57 and 2.90, suspected to be due to a large and broad water peak. **^{13}C NMR** (101 MHz, CD_3SOCD_3, 303 K) δ (ppm) = 173.2, 172.3, 171.6, 170.8, 159.3, 137.9, 137.9, 127.3, 119.3, 73.4, 70.3, 70.14, 70.06, 70.0, 69.5, 63.1, 58.9, 53.8, 41.8, 39.1, 39.1, 31.15, 31.07, 30.4, 29.4, 27.4, 19.6, 18.3. **LCMS**: HpH, RT = 0.59 min, $[M+H]^+$ = 786.3, 94% UV purity. **IR** (ATIR, cm-1): 3455, 3273, 2866, 1654, 1625, 1538, 1453, 1415, 1385, 1347, 1096. **HRMS**: formic, molecular formula ($C_{36}H_{63}N_7O_{12}$) *m/z* found $[M+H]^+$ 786.4605, $C_{36}H_{64}N_7O_{12}^+$ requires 786.4607.

(*rac*-(1*R*,8*S*,9*s*)-Bicyclo[6.1.0]non-4-yn-9-yl)methyl ((6*S*,9*S*)-1-amino-9-isopropyl-6-((4-((((4-nitrophenoxy)carbonyl)oxy)methyl)phenyl)carbamoyl)-1,8,11,14-tetraoxo-18,21,24,27,30,33-hexaoxa-2,7,10,15-tetraazapentatriacontan-35-yl)carbamate 16

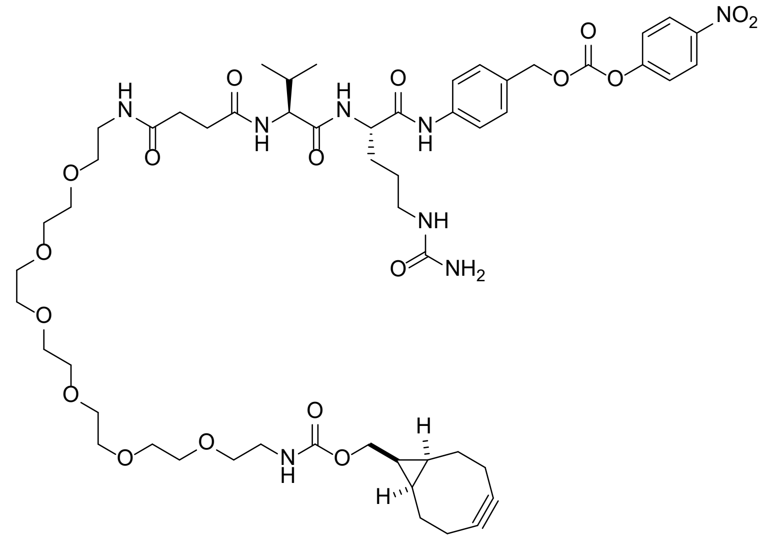

(*rac*-(1*R*,8*S*,9s)-Bicyclo[6.1.0]non-4-yn-9-yl)methyl (2,5-dioxopyrrolidin-1-yl) carbonate (17 mg, 59 µmol) was added to a solution of *N*1-(20-amino-3,6,9,12,15,18-hexaoxaicosyl)-*N*4-((*S*)-1-(((*S*)-1-((4-(hydroxymethyl)phenyl)amino)-1-oxo-5-ureidopentan-2-yl)amino)-3-methyl-1-oxobutan-2-yl)succinamide (42 mg, 53 µmol) in DMF (0.5 mL) and the reaction was stirred at r.t. for 1 h. To this, bis(4-nitrophenyl) carbonate (49 mg, 0.16 mmol) and DIPEA (37 µL, 0.21 mmol) were added and the reaction was stirred at r.t. for 20 h. The reaction mixture was directly purified by prep-HPLC (formic modified, 5-70% acetonitrile/water) and the appropriate fractions were combined and concentrated under reduced pressure. This was then dried under vacuum to afford (*rac*-(1*R*,8*S*,9s)-bicyclo[6.1.0]non-4-yn-9-yl)methyl ((6*S*,9*S*)-1-amino-9-isopropyl-6-((4-((((4-nitrophenoxy)carbonyl)oxy)methyl)phenyl)-carbamoyl)-1,8,11,14-tetraoxo-18,21,24,27,30,33-hexaoxa-2,7,10,15-tetraazapentatriacontan-35-yl)carbamate **16** (12 mg, 11 µmol, 20 %) as a pale yellow solid.

^{1}H NMR (600 MHz, CD_3SOCD_3, 300 K) δ (ppm) = 9.77 - 9.52 (m, 1H), 8.33 - 8.30 (m, 1H), 8.14 - 8.07 (m, 1H), 8.02 (dd, *J* = 7.4, 5.2 Hz, 1H), 8.00 - 7.94 (m, 1H), 7.88 (br d, *J* = 8.8 Hz, 1H), 7.71 (d, *J* = 8.4 Hz, 1H), 7.58 (t, *J* = 10.2 Hz, 2H), 7.42 (d, *J* = 8.6 Hz, 1H), 7.23 (d, *J* = 8.4 Hz, 1H), 7.07 (br s, 1H), 6.33 (br s, 1H), 5.97 (br d, *J* = 5.3 Hz, 1H), 5.39 (br d, *J* = 5.3 Hz, 2H), 5.25 (s, 1H), 4.43 (s, 1H), 4.40 - 4.31 (m, 1H), 4.19 - 4.08 (m, 1H), 4.06 - 3.99 (m, 2H), 3.54 - 3.47 (m, 20H), 3.17 - 3.10 (m, 4H), 3.02 (dt, *J* = 12.6, 6.3 Hz, 1H), 2.99 - 2.92 (m, 1H), 2.49 - 2.35 (m, 4H), 2.29 - 2.19 (m, 2H), 2.18 - 2.11 (m, 4H), 2.11 - 2.02 (m, 1H), 1.78 (dt, *J* = 9.0, 4.5 Hz, 1H), 1.66 (dt, *J* = 9.0, 4.5 Hz, 1H), 1.57 - 1.43 (m, 3H), 1.43 - 1.33 (m, 1H), 1.31 - 1.20 (m, 1H), 0.94 - 0.81 (m, 8H). Some signals missing, suspected to be under water signal, confirmed by 2D NMR. Some signal splitting observed indicating 2 species present in a 50:50 mixture. It is unclear whether this may be hydrolysis or a conformational isomer, minimal presence of *p*-nitrophenol present so carbonate may be intact. **^{13}C NMR** (151 MHz, CD_3SOCD_3, 300 K) δ (ppm) = 172.8, 171.8, 171.2, 170.5, 158.8, 156.4, 155.3, 151.9, 145.2, 138.4, 129.4, 129.3, 126.8, 118.9, 122.1, 99.0, 69.75, 69.70, 69.6, 69.5, 69.1, 69.0, 66.4, 61.3, 58.3, 53.3, 40.0, 38.7, 38.6, 30.6, 30.6, 29.9, 28.9, 28.6, 27.0, 20.8, 19.5, 19.2, 17.8, 17.5. 6 missing signals, suspected that PEG region is overlapping, confirmed by 2D NMR. Some peaks have split and 2D NMR suggests that two species are present. It is unclear if hydrolysis of the carbonate has partially occurred, or if there is some conformational isomerism.

Hydrolysis not observed by other analytical techniques and compound reacted as intended without issue. **LCMS**: HpH, RT = 1.11 min, $[M+H]^+$ = 1127.5. **IR** (ATIR, cm^{-1}): 3269, 2916, 2870, 1752, 1710, 1630, 1524, 1347, 1276, 1276, 1211, 1097. **HRMS**: molecular formula ($C_{54}H_{78}N_8O_{18}$) *m/z* found $[M+H]^+$ 1127.5503, $C_{54}H_{79}N_8O_{18}^+$ requires 1127.5507.

(9*H*-Fluoren-9-yl)methyl (*S*)-(1-((4-(hydroxymethyl)phenyl)amino)-1-oxopropan-2-yl)carbamate

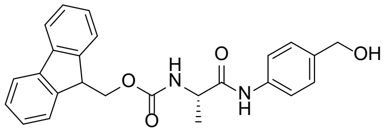

Ethyl 2-ethoxyquinoline-1(2*H*)-carboxylate (5.16 g, 21 mmol) was added to a stirred solution of (((9*H*-fluoren-9-yl)methoxy)carbonyl)-*L*-alanine (5.00 g, 16 mmol) in DCM (50 mL) and methanol (25 mL). This was stirred for 5 minutes, at r.t., before (4-aminophenyl)methanol (2.37 g, 19 mmol) was added in one portion. The reaction was stirred at r.t. for 4.5 h. The reaction mixture was diluted with diethyl ether (150 mL), nothing precipitated so the solution was concentrated to half volume under reduced pressure. A white precipitate had formed which was then filtered under vacuum. The filtrand was washed with ether (5 x 50 mL), dried in air and then collected, and dried under vacuum to afford (9*H*-fluoren-9-yl)methyl (*S*)-(1-((4-(hydroxymethyl)phenyl)amino)-1-oxopropan-2-yl)carbamate (4.5 g, 11 mmol, 67 % yield) a white solid.

^{1}H NMR (400 MHz, CD_3SOCD_3, 303 K) δ (ppm) = 9.92 (s, 1H), 7.90 (d, *J* = 7.3 Hz, 2H), 7.79 - 7.70 (m, 2H), 7.64 (br d, *J* = 7.3 Hz, 1H), 7.60 - 7.52 (m, 2H), 7.42 (br t, *J* = 7.5 Hz, 1H), 7.38 - 7.30 (m, 2H), 7.30 - 7.20 (m, 2H), 5.08 (t, *J* = 5.7 Hz, 1H), 4.44 (d, *J* = 5.7 Hz, 3H), 4.33 - 4.27 (m, 2H), 4.27 - 4.17 (m, 1H), 1.32 (d, *J* = 7.1 Hz, 4H). **^{13}C NMR** (101 MHz, CD_3SOCD_3, 303 K) δ (ppm) = 171.9, 156.3, 144.4, 144.3, 141.2, 138.1, 137.9, 128.1, 127.5, 127.3, 125.8, 120.6, 119.4, 66.1, 63.1, 51.2, 47.1, 18.6. Additional C from slight inequivalence of dibenzofulvene **LCMS** HpH, rt = 1.09 min, $[M+H]^+$ = 417.1, **IR**(ATIR, cm^{-1}): 3291 (br.) 1657, 1530, 1450, 1415, 1320, 1248, 1106, 1086, 1043, 755, 739, 646.

Consistent with previously reported analytical data.[6]

(*S*)-2-Amino-*N*-(4-(hydroxymethyl)phenyl)propanamide

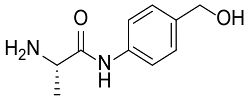

Piperidine (0.24 mL, 2.430 mmol) was added to a solution of (9*H*-fluoren-9-yl)methyl (*S*)-(1-((4-(hydroxymethyl)phenyl)amino)-1-oxopropan-2-yl)carbamate (503 mg, 1.208 mmol) in DMF (2.5 mL) and the reaction was stirred, at r.t. for 1 h. The reaction was diluted with water (100 mL) and cooled in an ice bath before being filtered under vacuum. The residue was then washed with water (3 x 50 mL) and the filtrate was collected and concentrated under reduced pressure. This was then purified by reverse-phase flash column chromatography (60 g, 0-20% 10 mM aq. ammonium carbonate solution/Acetonitrile). The appropriate fractions were combined and concentrated *in vacuo* to afford (*S*)-2-amino-*N*-(4-(hydroxymethyl)phenyl)propenamide (182 mg, 0.94 mmol, 78 % yield) as a beige, waxy solid.

^{1}H NMR (400 MHz, CD_3SOCD_3, 303 K) δ (ppm) = 11.17 - 8.88 (br. m, 1H), 7.63 - 7.54 (m, 2H), 7.28 - 7.20 (m, 2H), 5.08 (br s, 1H), 4.44 (s, 2H), 3.42 (q, *J* = 6.8 Hz, 1H), 1.22 (d, *J* = 6.8 Hz, 3H) **^{13}C NMR** (101 MHz, CD_3SOCD_3, 303 K) δ (ppm) = 175.2, 138.0, 137.7, 127.4, 119.3, 63.1, 51.5, 22.0 **LCMS** HpH, rt = 0.48 mins, $[M+H]^+$ = 195.0.

Consistent with previously reported analytical data.[6]

(9*H*-Fluoren-9-yl)methyl ((*S*)-1-(((*S*)-1-((4-(hydroxymethyl)phenyl)amino)-1-oxopropan-2-yl)amino)-3-methyl-1-oxobutan-2-yl)carbamate

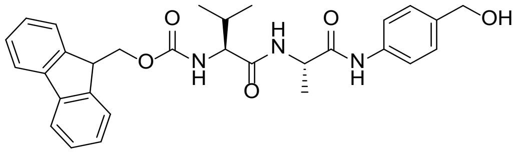

HATU (1.7 g, 4.5 mmol) was added to a stirred solution of (((9*H*-fluoren-9-yl)methoxy)carbonyl)-*L*-valine (1.3 g, 3.8 mmol), and DIPEA (1.6 mL, 9.2 mmol) in DMF (5 mL), this reaction was stirred for 10 minutes before being added to a stirred suspension of (*S*)-2-amino-*N*-(4-(hydroxymethyl)phenyl)-propanamide (600 mg, 3.1 mmol) in DMF (5 mL). The reaction was stirred for 3.5 h under air. The reaction mixture was diluted with ether (100 mL) and then filtered under vacuum to afford an orange solid. This was then triturated with water (100 mL) and filtered by vacuum. The residue was collected and dried under vacuum to afford (9*H*-fluoren-9-yl)methyl ((*S*)-1-(((*S*)-1-((4-(hydroxymethyl)phenyl)-amino)-1-oxopropan-2-yl)amino)-3-methyl-1-oxobutan-2-yl)carbamate (929 mg, 1.8 mmol, 58 % yield, 94% purity) as a white solid.

^{1}H NMR (400 MHz, CD_3SOCD_3, 303 K) δ (ppm) = 9.90 (s, 1H), 8.15 (d, *J* = 7.1 Hz, 1H), 7.89 (d, *J* = 7.6 Hz, 2H), 7.75 (t, *J* = 7.5 Hz, 2H), 7.54 (d, *J* = 8.6 Hz, 2H), 7.42 (br t, *J* = 6.8 Hz, 3H), 7.37 - 7.29 (m, 2H), 7.24 (d, *J* = 8.6 Hz, 2H), 5.75 (s, 1H), 5.09 (t, *J* = 5.7 Hz, 1H), 4.51 - 4.38 (m, 3H), 4.35 - 4.19 (m, 3H), 3.93 (dd, *J* = 7.0, 8.9 Hz, 1H), 2.01 (qd, *J* = 13.5, 6.8 Hz, 1H), 1.32 (d, *J* = 7.1 Hz, 3H), 0.91-0.86 (m, 6H) **^{13}C NMR** (101 MHz, CD_3SOCD_3, 303 K) δ (ppm) = 171.4, 171.3, 156.6, 144.3, 144.3, 141.2, 137.9, 128.1, 127.5, 127.4, 125.8, 120.6, 119.4, 66.2, 63.1, 60.5, 55.4, 49.5, 47.2, 30.9, 19.7, 18.7, 18.6, **LCMS** HpH, rt = 1.14 mins, $[M+H]^+$ = 516.1, **IR** (ATIR, cm^{-1}): 3282, 1690, 1643, 1534, 1449, 1290, 1248, 1033, 757, 737, 542.

Consistent with previously reported analytical data.[6]

(*S*)-2-Amino-*N*-((*S*)-1-((4-(hydroxymethyl)phenyl)amino)-1-oxopropan-2-yl)-3-methylbutanamide

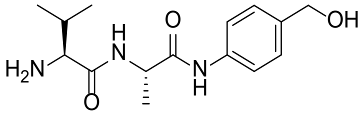

Piperidine (0.87 ml, 8.8 mmol) was added to a solution of (9*H*-fluoren-9-yl)methyl ((*S*)-1-(((*S*)-1-((4-(hydroxymethyl)phenyl)amino)-1-oxopropan-2-yl)amino)-3-methyl-1-oxobutan-2-yl)carbamate (2.26 g, 4.38 mmol) in DMF (11 ml) and the reaction was stirred at r.t. for 3 h. After this time a white precipitate had formed making stirring difficult. The reaction mixture was diluted with water (150 mL) and washed with DCM (3 x 150 mL). The aqueous phase was collected and concentrate under reduced pressure to afford a yellow oil. The residue was purified by reverse phase flash column chromatography (C18 120 g, 0-30% 10 mM aqueous ammonium carbonate/Acetonitrile). The appropriate fractions

were combined and concentrated under reduced pressure and dried under vacuum to afford (*S*)-2-amino-*N*-((*S*)-1-((4-(hydroxymethyl)phenyl)amino)-1-oxopropan-2-yl)-3-methylbutanamide (937 mg, 2.71 mmol, 61.9 % yield, 85% purity) as a colourless gum.

^{1}H NMR (400 MHz, CD_3SOCD_3, 303 K) δ (ppm) = 9.97 (s, 1H), 8.28 - 8.04 (m, 1H), 7.54 (d, *J* = 8.4 Hz, 2H), 7.25 (d, *J* = 8.4 Hz, 2H), 5.46 - 4.72 (m, 1H), 4.54 - 4.40 (m, 3H), 3.02 (br d, *J* = 4.2 Hz, 1H), 1.93 (dq, *J* = 12.0, 6.8 Hz, 1H), 1.31 (d, *J* = 6.8 Hz, 3H), 0.90 (d, *J* = 6.8 Hz, 3H), 0.80 (d, *J* = 6.8 Hz, 3H) **^{13}C NMR** (101 MHz, CD_3SOCD_3, 303 K) δ (ppm) = 174.7, 171.5, 138.0, 137.9, 127.4, 119.5, 63.1, 60.1, 49.0, 31.9, 20.0, 19.2, 17.4, **LCMS** HpH, rt = 0.58 mins, $[M+H]^+$ = 294.0.

Consistent with previously reported analytical data. [6]

4-(((*S*)-1-(((*S*)-1-((4-(hydroxymethyl)phenyl)amino)-1-oxopropan-2-yl)amino)-3-methyl-1-oxobutan-2-yl)amino)-4-oxobutanoic acid

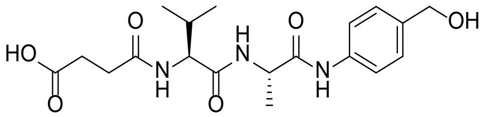

Succinic anhydride **23** (132 mg, 1.3 mmol) was added to a solution of (*S*)-2-amino-*N*-((*S*)-1-((4-(hydroxymethyl)phenyl)amino)-1-oxopropan-2-yl)-3-methylbutanamide (480 mg, 1.260 mmol) and triethylamine (0.26 mL, 1.9 mmol) in DMF (10 mL) and the reaction was stirred for 2 h at r.t.. The reaction mixture was concentrated under reduced pressure and then was purified by flash column chromatography (0-60% 0.1% formic acid in water/0.1% formic acid in acetonitrile). The appropriate fractions were combined and concentrated *in vacuo* to afford 4-(((*S*)-1-(((*S*)-1-((4-(hydroxymethyl)phenyl)amino)-1-oxopropan-2-yl)amino)-3-methyl-1-oxobutan-2-yl)amino)-4-oxobutanoic acid (409 mg, 1.04 mmol, 83 % yield) as a white solid.

^{1}H NMR (400 MHz, CD_3SOCD_3, 303 K) δ (ppm) = ^{1}H NMR (400 MHz, CD_3SOCD_3, 303 K) δ (ppm) = 12.06 (br s, 1H), 9.74 (s, 1H), 8.09 (d, *J* = 7.1 Hz, 1H), 7.92 (d, *J* = 8.3 Hz, 1H), 7.56 (d, *J* = 8.6 Hz, 2H), 7.26 (d, *J* = 8.6 Hz, 2H), 5.08 (br s, 1H), 4.47 - 4.36 (m, 3H), 4.17 (dd, *J* = 8.3, 6.6 Hz, 1H), 2.49 - 2.39 (m, 4H), 2.00 (qd, *J* = 13.5, 6.6 Hz, 1H), 1.32 (d, *J* = 7.1 Hz, 3H), 0.89 (br d, *J* = 6.6 Hz, 3H), 0.86 (br d, *J* = 6.6 Hz, 3H). **^{13}C NMR** (101 MHz, CD_3SOCD_3, 303 K) δ (ppm) = 174.4, 172.0, 171.4, 171.3, 138.0, 137.9, 127.4, 119.3, 63.1, 58.3, 49.5, 30.9, 30.4, 29.7, 19.6, 18.6, 18.4. **LCMS** formic, rt = 0.59 mins, MH+ = 394.2, **IR** (ATIR, cm^{-1}): 3312, 3268, 2965, 1712, 1637, 1534, 1414, 1395, 1245, 1181, 1037, 1021, 812, 717, 552, 508. **HRMS (ESI)** molecular formula ($C_{19}H_{27}N_3O_6$) *m/z* found $[M+H]^+$ 394.1977, $C_{19}H_{28}N_3O_6^+$ requires 394.1973.

(*rac*-(1*R*,8*S*,9s)-Bicyclo[6.1.0]non-4-yn-9-yl)methyl ((*S*)-3-methyl-1-(((*S*)-1-((4-((((4-nitrophenoxy)carbonyl)oxy)methyl)phenyl)amino)-1-oxopropan-2-yl)amino)-1-oxobutan-2-yl)carbamate 6

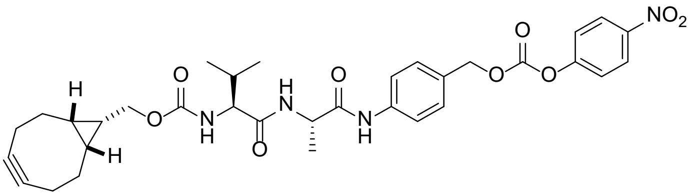

N-[(*rac*-(1*R*,8*S*,9s)-Bicyclo[6.1.0]non-4-yn-9-yl)methyloxycarbonyloxy]succinimide (60 mg, 0.21 mmol) was added to a solution of (*S*)-2-amino-*N*-((*S*)-1-((4-(hydroxymethyl)phenyl)amino)-1-oxopropan-2-yl)-3-methylbutanamide (40 mg, 0.14 mmol) in DMF (1 mL) and the reaction was stirred at r.t. for 1 h. After this time bis(*p*-nitrophenyl)carbonate (83 mg, 0.27 mmol) and DIPEA (53 mg, 71 µL, 0.41 mmol) were added and the reaction was stirred for a further 3 h at r.t. The reaction mixture was directly purified by Prep-HPLC (EZPrep, formic, 30-85% modified acetonitrile/modified water) and the appropriate fractions were combined and concentrated under reduced pressure. This was then dried under vacuum to afford (*rac*-(1*R*,8*S*,9s)-bicyclo[6.1.0]non-4-yn-9-yl)methyl ((*S*)-3-methyl-1-(((*S*)-1-((4-((((4-nitrophenoxy)carbonyl)oxy)methyl)phenyl)amino)-1-oxopropan-2-yl)amino)-1-oxobutan-2-yl)carbamate **6** (26 mg, 41 µmol, 30 %) as a white solid.

^{1}H NMR (400 MHz, CD_3SOCD_3, 303 K) δ (ppm) = 10.04 (s, 1H), 8.36 - 8.28 (m, 1H), 7.64 (d, *J* = 8.6 Hz, 1H), 7.60 - 7.55 (m, 1H), 7.46 - 7.38 (m, 2H), 7.24 (d, *J* = 8.6 Hz, 1H), 7.11 (br d, *J* = 8.8 Hz, 1H), 5.25 (s, 1H), 4.50 - 4.40 (m, 1H), 4.07 (d, *J* = 8.1 Hz, 2H), 3.89 (br t, *J* = 7.6 Hz, 1H), 2.26 - 2.08 (m, 6H), 1.99 (qd, *J* = 13.4, 6.7 Hz, 1H), 1.59 - 1.46 (m, 2H), 1.34 - 1.24 (m, 4H), 0.93-0.81 (m, 8H). Partial hydrolysis of carbonate observed by NMR, not observed by LCMS so decomposition has occurred after preparation of the NMR sample. **^{13}C NMR** (101 MHz, CD_3SOCD_3, 303 K) δ (ppm) = 171.7, 171.5, 156.9, 155.8, 152.4, 145.6, 139.9, 129.9, 129.8, 125.8, 123.0, 119.5, 99.4, 70.7, 62.1, 60.5, 49.5, 30.8, 29.0, 21.3, 20.1, 19.6, 18.6, 18.5, 18.1. **LCMS**: Formic, rt = 1.26 min, $[M+H]^+$ = 635.2, 99% purity by UV-Vis. **IR** (ATIR, cm^{-1}): 3299, 2966, 2929, 1764, 1648, 1608, 1520, 1346, 1207, 1023. **HRMS (ESI)** molecular formula ($C_{33}H_{38}N_4O_9$) *m/z* found $[M+H]^+$ 635.2713, $C_{33}H_{39}N_4O_9^+$ requires 635.2712.

***N*1-(2-(2-(2-(2-Azidoethoxy)ethoxy)ethoxy)ethyl)-*N*4-((*S*)-1-(((*S*)-1-((4-(hydroxymethyl)phenyl)amino)-1-oxopropan-2-yl)amino)-3-methyl-1-oxobutan-2-yl)succinamide**

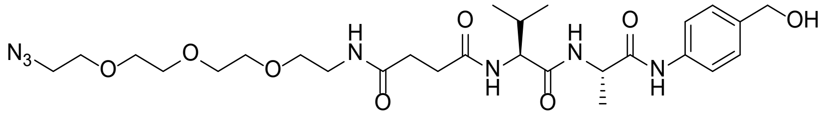

2-(3*H*-[1,2,3]Triazolo[4,5-b]pyridin-3-yl)-1,1,3,3-tetramethylisouronium hexafluorophosphate (V) (29 mg, 1.2 Eq, 76 µmol) was added to a solution of 4-(((*S*)-1-(((*S*)-1-((4-(hydroxymethyl)phenyl)amino)-1-oxopropan-2-yl)amino)-3-methyl-1-oxobutan-2-yl)amino)-4-oxobutanoic acid **25** (25 mg, 1 Eq, 64 µmol) and DIPEA (16 mg, 22 µL, 0.13 mmol) in DMF (0.5 mL) and the reaction was stirred at r.t. for 5 mins. After this time a solution of 2-(2-(2-(2-azidoethoxy)ethoxy)ethoxy)ethan-1-amine (21 mg, 95 µmol) in DMF (0.1 mL) was added and the reaction was stirred for a further 2 h. The reaction mixture

was directly purified by Prep-HPLC (EZPrep, HpH, 10-55% acetonitrile/modified water) and the appropriate fractions were combined and concentrated under a stream of nitrogen. This was then dried under vacuum to afford *N*1-(2-(2-(2-(2-azidoethoxy)ethoxy)ethoxy)ethyl)-*N*4-((*S*)-1-(((*S*)-1-((4-(hydroxymethyl)phenyl)amino)-1-oxopropan-2-yl)amino)-3-methyl-1-oxobutan-2-yl)succinimide (25 mg, 42 µmol, 66 %) as a white solid.

^{1}H NMR (400 MHz, CD_3SOCD_3, 303 K) δ (ppm) = 9.49 (s, 1H), 8.14 (d, *J* = 7.3 Hz, 1H), 8.02 (d, *J* = 7.6 Hz, 1H), 7.93 (t, *J* = 5.6 Hz, 1H), 7.59 (d, *J* = 7.8 Hz, 2H), 7.24 (d, *J* = 8.6 Hz, 2H), 5.08 (br s, 1H), 4.44 (s, 2H), 4.38 (quin, *J* = 7.3 Hz, 1H), 4.11 (dd, *J* = 7.6, 5.9 Hz, 1H), 3.61 - 3.58 (m, 2H), 3.58 - 3.48 (m, 6H), 3.48 - 3.43 (m, 2H), 3.40 - 3.37 (m, 2H), 3.37 - 3.29 (m, 6H), 3.15 (q, *J* = 5.8 Hz, 2H), 2.49 - 2.33 (m, 4H), 2.06 (dq, *J* = 13.1, 6.8 Hz, 1H), 1.35 (d, *J* = 7.1 Hz, 3H), 0.91 (br d, *J* = 6.8 Hz, 3H), 0.88 (br d, *J* = 6.8 Hz, 3H). Excess protons are suspected to come from water. **^{13}C NMR** (101 MHz, CD_3SOCD_3, 303 K) δ (ppm) = 173.3, 172.2, 171.34, 171.30, 138.0, 137.9, 127.3, 119.3, 70.3, 70.18, 70.16, 70.0, 69.7, 69.4, 63.1, 58.8, 50.5, 49.5, 39.0, 31.1, 31.0, 30.3, 19.6, 18.3, 18.1. **LCMS**: Formic, RT = 0.73 min, [M+H]+ = 594.2, 100% purity by UV-Vis. **IR** (ATIR, cm^{-1}): 3273, 3070, 2934, 2870, 2102, 1629, 1535, 1445, 1413, 1385, 1303, 1104. **HRMS (ESI)** molecular formula ($C_{27}H_{43}N_7O_8$) *m/z* found $[M+H]^+$ 594.3250, $C_{27}H_{44}N_7O_8^+$ requires 594.3246.

4-((18*S*,21*S*)-1-Azido-18-isopropyl-21-methyl-13,16,19-trioxo-3,6,9-trioxa-12,17,20-triazadocosan-22-amido)benzyl (4-nitrophenyl) carbonate 9

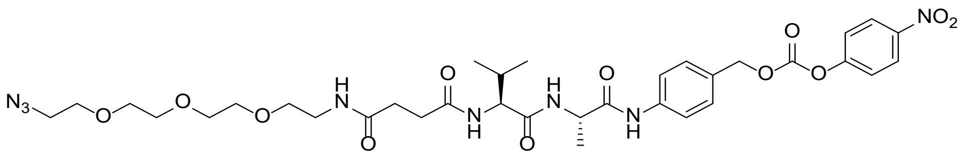

DIPEA (28 µL, 0.16 mmol) was added to a solution of *N*1-(2-(2-(2-(2-azidoethoxy)ethoxy)ethoxy)ethyl)-*N*4-((*S*)-1-(((*S*)-1-((4-(hydroxymethyl)phenyl)amino)-1-oxopropan-2-yl)amino)-3-methyl-1-oxobutan-2-yl)succinimide (27 mg, 0.045 mmol) and bis(4-nitrophenyl) carbonate (36 mg, 0.118 mmol) and the reaction was stirred at r.t. for 1 h. The reaction mixture was directly purified by prep-HPLC (formic, 10-70%, 20 mins) The appropriate fractions were combined and concentrated under reduced pressure to afford a white solid. This residue was dried under vacuum to afford4-((18*S*,21*S*)-1-azido-18-isopropyl-21-methyl-13,16,19-trioxo-3,6,9-trioxa-12,17,20-triazadocosan-22-amido)benzyl (4-nitrophenyl) carbonate **9** (26 mg, 0.035 mmol, 77 %yield) as a white solid.

^{1}H NMR (400 MHz, CD_3SOCD_3, 303 K) δ (ppm) = 9.65 (s, 1H), 8.37 - 8.27 (m, 2H), 8.16 (d, *J* = 7.3 Hz, 1H), 8.03 (br d, *J* = 7.6 Hz, 1H), 7.94 (br t, *J* = 5.6 Hz, 1H), 7.70 (d, *J* = 8.6 Hz, 2H), 7.63 - 7.52 (m, 2H), 7.42 (d, *J* = 8.8 Hz, 2H), 5.25 (s, 2H), 4.45 - 4.34 (m, 1H), 4.11 (dd, *J* = 7.5, 6.0 Hz, 1H), 3.61 - 3.34 (m, 14H), 3.15 (q, *J* = 5.8 Hz, 2H), 2.49 - 2.33 (m, 4H), 2.06 (qd, *J* = 13.1, 6.7 Hz, 1H), 1.36 (d, *J* = 7.1 Hz, 3H), 0.92 (d, *J* = 6.8 Hz, 3H), 0.89 (d, *J* = 6.8 Hz, 3H) **^{13}C NMR** (101 MHz, CD_3SOCD_3, 303 K) δ (ppm) = 173.3, 172.2, 171.7, 171.4, 155.8, 152.4, 145.7, 139.8, 129.9, 129.8, 125.9, 123.1, 119.5, 70.7, 70.3 70.17, 70.16, 70.0, 69.7, 69.4, 58.8, 50.5, 49.6, 39.0, 31.1, 31.0, 30.4, 19.6, 18.4, 18.0 **LCMS** HpH, rt = 1.10 mins, MH+ = 759.3, **IR**(ATIR, cm $^{-1}$): 3276, 2871, 2102, 1763, 1630, 1523, 1347, 1251, 1208, 1110, 864, 839, 667. **HRMS (ESI)** molecular formula ($C_{34}H_{46}N_8O_{12}$) *m/z* found $[M+H]^+$ 759.3312, $C_{34}H_{47}N_8O_{12}^+$ requires 759.3308.

b. Non-cleavable linkers 7 and 10

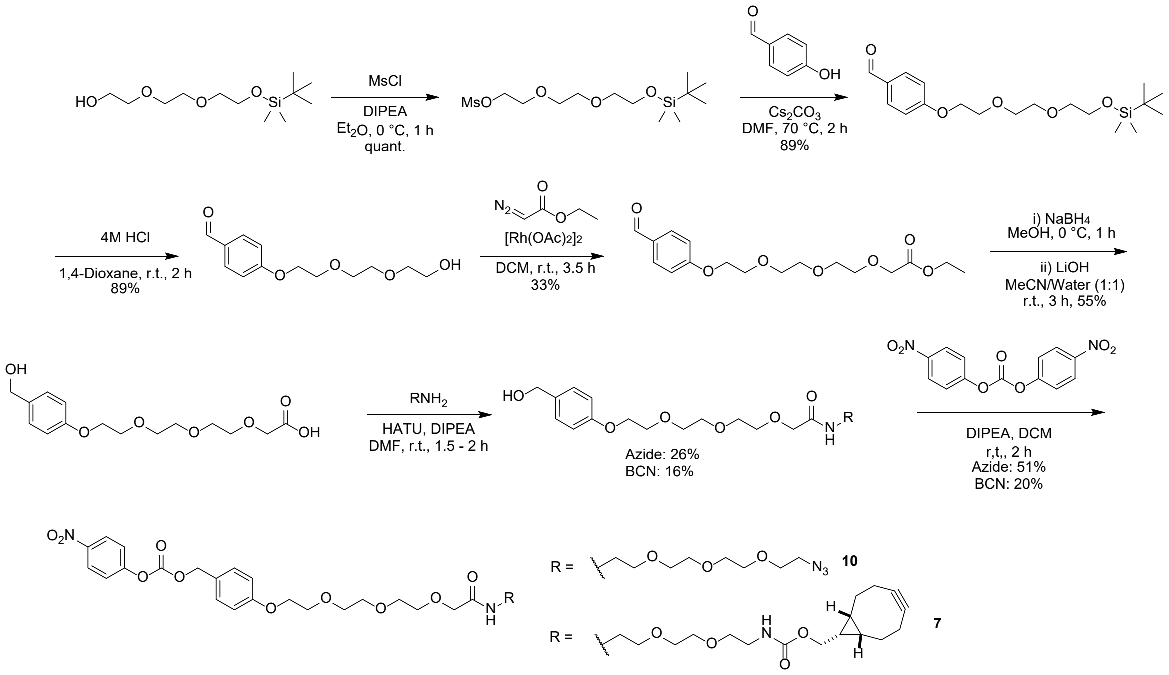

Scheme SI-3. Synthesis route for the preparation of non-cleavable linkers **7** and **10**

2,2,3,3-Tetramethyl-4,7,10-trioxa-3-siladodecan-12-ol

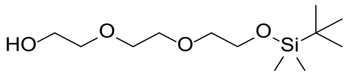

A solution of 2,2'-(ethane-1,2-diylbis(oxy))bis(ethan-1-ol) (5.0 g, 33 mmol DIPEA (1.4 mL, 8.0 mmol) and DMAP (0.16 g, 1.3 mmol) in DCM (15 mL) was cooled to 0 °C in an ice bath. To this, a solution of *tert*-butyldimethlsilyl chloride (1 g, 6.6 mmol) in DCM (10 mL) was added dropwise over 20 mins and the reaction was then stirred for 20 h at r.t.. After this time the reaction mixture was diluted with DCM (30 mL) and washed with sat. aq. ammonium chloride solution (60 mL), brine (60 mL) and the organic phase was then filtered through a hydrophobic frit and concentrated under reduced pressure to afford a colourless oil. The residue was purified by flash column chromatography (RediSep silica 24 g, 0-100% ethyl acetate/cyclohexane). The appropriate fractions were combined and concentrated *in vacuo* to afford 2,2,3,3-tetramethyl-4,7,10-trioxa-3-siladodecan-12-ol (1.2 g, 4.7 mmol, 70 % yield) as a colourless oil.

^{1}H NMR (400 MHz, $CDCl_3$, 303 K) δ (ppm) = 3.82 - 3.77 (m, 2H), 3.75 (br d, *J* = 4.2 Hz, 2H), 3.69 (s, 4H), 3.66 - 3.61 (m, 2H), 3.61 - 3.58 (m, 2H), 2.44 (br s, 1H), 0.92 (s, 9H), 0.09 (s, 6H). **^{13}C NMR** (101 MHz, $CDCl_3$, 303 K) δ (ppm) = 72.7, 72.5, 70.8, 70.5, 62.7, 61.8, 25.9, 18.4, -5.3. **IR** (ATIR, cm^{-1}): 3446 (br.), 2928, 2857, 1472, 1463, 1361, 1252, 1100, 939, 831, 775, 719.

Consistent with previously reported analytical data.[7]

2,2,3,3-Tetramethyl-4,7,10-trioxa-3-siladodecan-12-yl methanesulfonate

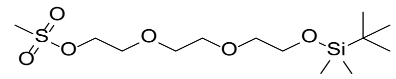

Methanesulfonylchloride (0.44 mL, 5.7 mmol) was added dropwise over 10 min to a cooled solution (0 °C) of 2,2,3,3-tetramethyl-4,7,10-trioxa-3-siladodecan-12-ol (1 g, 3.78 mmol) and DIPEA (1.3 mL, 7.6 mmol) in diethyl ether (15 mL) and the reaction was stirred at 0 °C for 1 h under nitrogen. The reaction mixture was diluted with DCM (75 mL) and was washed with sat. aq. ammonium chloride solution (75 mL) and brine solution (75 mL) and the organic phase was filtered through a hydrophobic frit and then concentrated under reduced pressure. The residue was purified by flash column chromatography (RediSep silica 24 g, 0-100% ethyl acetate/cyclohexane). The appropriate fractions were combined and concentrated *in vacuo* to afford 2,2,3,3-tetramethyl-4,7,10-trioxa-3-siladodecan-12-yl methanesulfonate, (1.3 g, 3.8 mmol, quant.) as a pale-yellow oil.

^{1}H NMR (400 MHz, $CDCl_3$, 303 K) δ (ppm) = 4.40 - 4.36 (m, 2H), 3.80 - 3.73 (m, 4H), 3.66 (s, 4H), 3.59 - 3.52 (m, 2H), 3.07 (s, 3H), 0.91 (s, 9H), 0.08 (s, 6H). **^{13}C NMR** (101 MHz, $CDCl_3$, 303 K) δ (ppm) = 72.7, 70.8, 70.7, 69.2, 69.1, 62.7, 37.7, 25.9, 18.4, -5.3. **IR** (ATIR, cm^{-1}): 2929, 2857, 1483, 1352, 1252, 1174, 1099, 1018, 968, 919, 832, 810, 776, 731, 663, 527, 456

Consistent with previously reported analytical data.[7]

4-((2,2,3,3-Tetramethyl-4,7,10-trioxa-3-siladodecan-12-yl)oxy)benzaldehyde

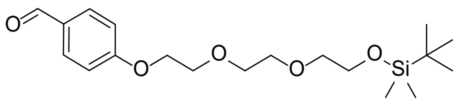

4-hydroxybenzaldehyde (1.50 g, 12.3 mmol), 2,2,3,3-tetramethyl-4,7,10-trioxa-3-siladodecan-12-yl methanesulfonate (3.50 g, 10.2 mmol), and Cs_2CO_3 (6.66 g, 20.4 mmol) were suspended in DMF (25 mL), and the reaction was stirred at 70 °C for 2 h. The reaction mixture was diluted with ethyl acetate (200 mL) and then washed with half sat. aq. ammonium chloride (200 mL), sat. aq. ammonium chloride (100 mL), sat. aq. sodium hydrogen carbonate (100 mL), and brine (100 mL). The organic phase was then dried over magnesium sulfate, and then filtered by vacuum to afford a pale yellow oil. The residue was purified by Flash column chromatography (RediSep silica, 120 g, 0-40% ethyl acetate/cyclohexane) and the appropriate fractions were combined and concentrated under reduced pressure. The residue was re-purified by Flash-column chromatography (RediSep silica, 120 g, 0-50% ethyl acetate/cyclohexane, spiked with 1% Et_3N) and the appropriate fractions were combined and concentrated under reduced pressure. This was then dried in vacuum to afford 4-((2,2,3,3-tetramethyl-4,7,10-trioxa-3-siladodecan-12-yl)oxy)benzaldehyde, , (3.4 g, 9.1 mmol, 89 %) as a colourless oil.

^{1}H NMR (400 MHz, $CDCl_3$, 303 K) δ (ppm) = 9.91 (s, 1H), 7.87 - 7.82 (m, 2H), 7.06 - 7.02 (m, 2H), 4.26 - 4.20 (m, 2H), 3.95 - 3.88 (m, 2H), 3.80 - 3.69 (m, 6H), 3.61 - 3.56 (m, 2H), 0.91 (s, 9H), 0.08 (s, 6H). **^{13}C NMR** (101 MHz, $CDCl_3$, 303 K) δ (ppm) = 190.7, 163.9, 131.9, 130.1, 114.9, 72.8, 71.0, 70.8, 69.5, 67.8, 62.7, 25.9, 18.4, -5.3. **LCMS**: HpH, rt =1.42 mins, $[M+H]^+$ = 369.1, 100% pure by UV. **IR** (ATIR, cm^{-1}): 2928, 2856, 1694, 1600, 1509, 1253, 1102, 939, 829, 776, 653, 619, 514. **HRMS (ESI)** molecular formula ($C_{19}H_{32}O_5Si$) *m/z* found $[M+H]^+$ 369.2107, $C_{19}H_{33}O_5Si^+$ requires 369.2092.

4-(2-(2-(2-Hydroxyethoxy)ethoxy)ethoxy)benzaldehyde

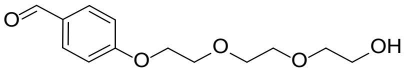

4 M HCl in 1,4-dioxane (1.8 g, 12 mL, 4 molar, 48 mmol) was added to a solution of 4-((2,2,3,3-tetramethyl-4,7,10-trioxa-3-siladodecan-12-yl)oxy)benzaldehyde (3.3 g, 9.0 mmol) in 1,4-Dioxane (12 mL) and the reaction was stirred at r.t. for 2 h. The reaction mixture was concentrated under reduced pressure and the residue was purified by flash column chromatography (RediSep silica, 80 g, 0-100% ethyl acetate/cyclohexane). The appropriate fractions were combined and concentrated under reduced pressure and then dried in vacuum to afford 4-(2-(2-(2-hydroxyethoxy)ethoxy)ethoxy)-benzaldehyde (2.1 g, 8.1 mmol, 89 %) as a colourless oil.

^{1}H NMR (400 MHz, $CDCl_3$, 303 K) δ (ppm) = 9.90 (s, 1H), 7.87 - 7.82 (m, 2H), 7.07 - 7.02 (m, 2H), 4.27 - 4.21 (m, 2H), 3.94 - 3.88 (m, 2H), 3.77 - 3.70 (m, 6H), 3.66 - 3.61 (m, 2H), 2.35 (br s, 1H). **^{13}C NMR** (101 MHz, $CDCl_3$, 303 K) δ (ppm) = 190.7, 163.8, 131.9, 130.2, 114.9, 72.5, 70.9, 70.4, 69.5, 67.7, 61.8. **IR** (ATIR, cm^{-1}): 3456 (br.), 2871, 1682, 1598, 1577, 1509, 1453, 1311, 1254, 1215, 1160, 1111, 1054, 924, 831, 652, 618, 514. **LCMS**: Formic, MH^+: 255.0, RT: 0.72, 94% purity by UV. **HRMS (ESI)** molecular formula ($C_{13}H_{18}O_5$) *m/z* found $[M+H]^+$ 255.1244, $C_{13}H_{19}O_5^+$ requires 255.1227.

Ethyl 2-(2-(2-(2-(4-formylphenoxy)ethoxy)ethoxy)ethoxy)acetate

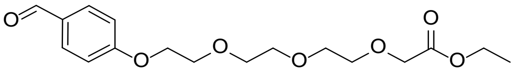

A solution of ethyl 2-diazoacetate solution in DCM (497 µL, 87% Wt, 3.93 mmol) in DCM (10 mL)was added dropwise over 2 h to a stirred solution of 4-(2-(2-(2-hydroxyethoxy)ethoxy)ethoxy)-benzaldehyde (500 mg, 1.97 mmol) and rhodium(II) acetate dimer (87 mg, 197 µmol) in DCM (10 mL) and the reaction was stirred at r.t. for 3 h. The reaction mixture was filtered through a 10 g Celite cartridge, which was then washed with DCM (3 x 20 mL). The filtrates were collected and concentrated under reduced pressure, this residue was filtered by flash column chromatography (RediSep silica, 12 g, 0-100% ethyl acetate/cyclohexane) and the appropriate fractions were combined and concentrated under reduced pressure. This was then dried in vacuum to afford ethyl 2-(2-(2-(2-(4-formylphenoxy)ethoxy)ethoxy)ethoxy)acetate (248 mg, 729 µmol, 37.1 %) as a yellow oil.

^{1}H NMR (400 MHz, $CDCl_3$, 303 K) δ (ppm) = 9.90 (s, 1H), 7.84 (d, *J* = 8.1 Hz, 2H), 7.04 (d, *J* = 7.9 Hz, 2H), 4.27 - 4.17 (m, 4H), 4.15 (s, 2H), 3.95 - 3.87 (m, 2H), 3.77 - 3.69 (m, 8H), 1.29 (t, *J* = 7.2 Hz, 3H). **^{13}C NMR** (101 MHz, $CDCl_3$, 303 K) δ (ppm) = 190.7, 170.4, 163.9, 131.9, 130.1, 114.9, 70.9, 70.68, 70.66, 69.5, 68.7, 67.8, 60.8, 14.2. 1 signal not observed, suspected overlap of PEG signals. **LCMS**: For, rt = 0.99 min, [MH]+ = 340.9, 96% purity by UV. **IR**(ATIR, cm-1): 2873 (br.), 1749, 1684, 1599, 1578, 1509, 1453, 1310, 1255, 1211, 1108, 1053, 1030, 924, 833, 652, 619, 516. **HRMS (ESI)** molecular formula ($C_{17}H_{24}O_7$) *m/z* found $[M+H]^+$ 341.1609, $C_{17}H_{25}O_7^+$ requires 341.1595.

2-(2-(2-(2-(4-(Hydroxymethyl)phenoxy)ethoxy)ethoxy)ethoxy)acetic acid

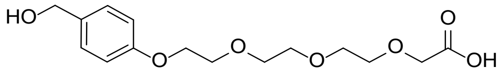

A solution of ethyl 2-(2-(2-(2-(4-formylphenoxy)ethoxy)ethoxy)ethoxy)acetate (120 mg, 353 µmol) in Methanol (2 mL) was cooled to 0 °C in an ice bath. To this, sodium borohydride (20 mg, 0.53 mmol) was added and the reaction was stirred at 0 °C for 1 h. The reaction mixture was concentrated under reduced pressure and then redissolved in acetonitrile (1 mL) and water (1 mL). To this, lithium hydroxide monohydrate (59 mg, 1.4 mmol) was added, and the reaction was stirred at r.t. for 3 h. The reaction mixture was directly purified by Prep-HPLC (EZPrep, formic modified, 5-40% 0.1% formic acid in acetonitrile/0.1% formic acid in water) and the appropriate fractions were combined and concentrated under reduced pressure. This was then dried under vacuum to afford 2-(2-(2-(2-(4-(hydroxymethyl)phenoxy)ethoxy)ethoxy)ethoxy)acetic acid (61 mg, 0.19 mmol, 55 %) as a colourless oil.

^{1}H NMR (400 MHz, $CDCl_3$, 303 K) δ (ppm) = 7.29 (d, *J* = 8.6 Hz, 2H), 6.92 (d, *J* = 8.6 Hz, 2H), 4.62 (s, 2H), 4.16 (dd, *J* = 5.3, 4.0 Hz, 2H), 4.11 (s, 2H), 3.87 (dd, *J* = 5.5, 4.0 Hz, 2H), 3.78 - 3.67 (m, 8H). **^{13}C NMR** (101 MHz, $CDCl_3$, 303 K) δ (ppm) = 172.3, 158.3, 133.4, 128.7, 114.7, 71.2, 70.63, 70.58, 70.2, 69.8, 69.0, 67.5, 64.9. **LCMS**: Formic, RT = 0.61 min, $[M-H]^-$ = 313.0, 93% pure by UV. **IR** (cm-1, ATIR) = 3220 (br.), 2874, 1733, 1611, 1512, 1355, 1300, 1243, 1101. **HRMS (ESI)** molecular formula ($C_{15}H_{22}O_7$) *m/z* found $[M+Na]^+$ 337.1271, $C_{15}H_{22}O_7Na^+$ requires 337.1258.

N-(2-(2-(2-(2-Azidoethoxy)ethoxy)ethoxy)ethyl)-2-(2-(2-(2-(4-(hydroxymethyl)phenoxy)ethoxy)ethoxy)ethoxy)acetamide

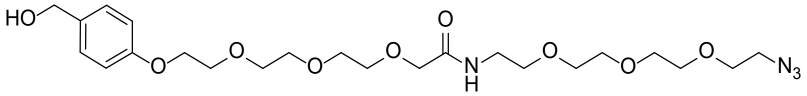

HATU (86 mg, 0.23 mmol) was added to a solution of 2-(2-(2-(2-(4-(hydroxymethyl)phenoxy)ethoxy)ethoxy)ethoxy)acetic acid (55 mg, 0.17 mmol) and DIPEA (45 mg, 61 µL, 0.35 mmol) in DMF (0.5 mL) and the reaction was stirred for 15 mins at r.t. before a solution of 11-Azido-3,6,9-trioxaundecylamine (57 mg, 52 µL, 0.26 mmol) in DMF (0.5 mL) was added, and the reaction was stirred for a further 1.5 h. The reaction mixture was directly purified by Prep-HPLC (EZPrep, 5-40% 0.1% formic acid in acetonitrile/ 0.1% formic acid in water) and the appropriate fractions were combined and concentrated under reduced pressure. This was then dried under vacuum to afford *N*-(2-(2-(2-(2-azidoethoxy)ethoxy)ethoxy)ethyl)-2-(2-(2-(2-(4-(hydroxymethyl)-phenoxy)ethoxy)ethoxy)ethoxy)acetamide (23 mg, 45 µmol, 26 %) as a colourless oil.

^{1}H NMR (400 MHz, $CDCl_3$, 303 K) δ (ppm) = 7.38 - 7.25 (m, 2H), 7.20 (br s, 1H), 6.91 (d, *J* = 7.7 Hz, 2H), 4.61 (s, 2H), 4.18 - 4.12 (m, 2H), 3.95 (s, 2H), 3.88 - 3.84 (m, 2H), 3.77 - 3.73 (m, 2H), 3.71 - 3.65 (m, 17H), 3.65 - 3.60 (m, 4H), 3.59 - 3.54 (m, 2H), 3.52 - 3.45 (m, 2H), 3.39 (q, *J* = 5.1 Hz, 4H), 2.18 - 2.01 (m, 1H). ^{13}C NMR (101 MHz, $CDCl_3$, 303 K) δ (ppm) = 170.0, 158.3, 133.6, 128.6, 114.7, 70.9, 70.8, 70.71, 70.69, 70.66, 70.6, 70.5, 70.30, 70.25, 70.0, 69.8, 67.5, 64.9, 50.71, 50.69, 38.6. **LCMS**: Formic, RT = 0.78 min, [M+H]+ = 515.1, 91% purity by UV-Vis. **IR** (ATIR, cm^{-1}): 3413 (br.), 2869, 2099, 1660,

1538, 1512, 1455, 1346, 1285, 1244, 1102. HRMS (ESI) molecular formula ($C_{23}H_{38}N_4O_9$) *m/z* found $[M+Na]^+$ 537.2537, $C_{23}H_{38}N_4O_9Na^+$ requires 537.2531.

4-((23-Azido-11-oxo-3,6,9,15,18,21-hexaoxa-12-azatricosyl)oxy)benzyl (4-nitrophenyl)-carbonate 10

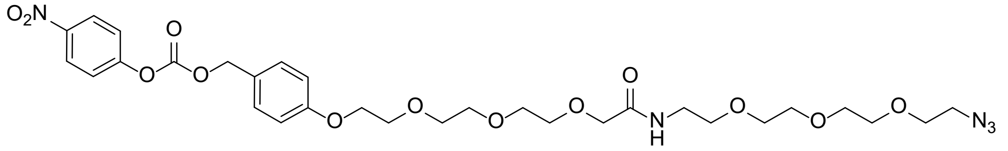

Bis(4-nitrophenyl) carbonate (89 mg, 0.29 mmol) was added to a solution of *N*-(2-(2-(2-(2-azidoethoxy)ethoxy)ethoxy)ethyl)-2-(2-(2-(2-(4-(hydroxymethyl)phenoxy)ethoxy)ethoxy)ethoxy)-acetamide (50 mg, 97 µmol) and DIPEA (68 µL, 0.39 mmol) in DCM (0.5 mL) and the reaction was stirred at r.t. for 2 h. The reaction mixture was directly purified by flash-column chromatography (RediSep silica, 12 g, 0-25% ethanol/ethyl acetate) and the appropriate fractions were combined and concentrated under reduced pressure. This was then dried under vacuum to afford to afford 4-((23-azido-11-oxo-3,6,9,15,18,21-hexaoxa-12-azatricosyl)oxy)benzyl (4-nitrophenyl) carbonate **10** (39 mg, 49 µmol, 51 %) as a yellow oil.

^{1}H NMR (400 MHz, $CDCl_3$, 303 K) δ (ppm) = 8.33 - 8.23 (m, 2H), 7.43 - 7.34 (m, 4H), 7.20 (br s, 1H), 6.98 - 6.93 (m, 2H), 5.25 (s, 2H), 4.19 - 4.15 (m, 2H), 4.02 (s, 2H), 3.91 - 3.87 (m, 2H), 3.78 - 3.74 (m, 2H), 3.72 - 3.61 (m, 16H), 3.61 - 3.57 (m, 2H), 3.53 - 3.48 (m, 2H), 3.39 (t, *J* = 5.0 Hz, 2H). 95% purity with 3% *p*-nitrophenol impurity. **^{13}C NMR** (101 MHz, $CDCl_3$, 303 K) δ (ppm) = 170.1, 159.4, 159.4, 155.6, 145.4, 130.6, 126.6, 125.3, 121.8, 114.8, 71.0, 70.9, 70.8, 70.7, 70.63, 70.56, 70.4, 70.3, 70.0, 69.8, 69.7, 67.5, 50.7, 38.7. 2 signals missing, suspected to be due to PEG signals overlapping. **LCMS**: formic, RT = 1.14 min, $[M+H]^+$ = 680.1, 94% pure by UV-Vis. **IR** (ATIR, cm-1): 3347, 2870, 2101, 1763, 1672, 1614, 1516, 1346, 1207, 1106. **HRMS** (ESI) molecular formula ($C_{30}H_{41}N_5O_{13}$) *m/z* found $[M+H]^+$ 680.2775, $C_{30}H_{42}N_5O_{13}^+$ requires 680.2774.

(*rac*-(1*R*,8*S*,9*s*)-bicyclo[6.1.0]non-4-yn-9-yl)methyl (20-(4-(hydroxymethyl)phenoxy)-10-oxo-3,6,12,15,18-pentaoxa-9-azaicosyl)carbamate

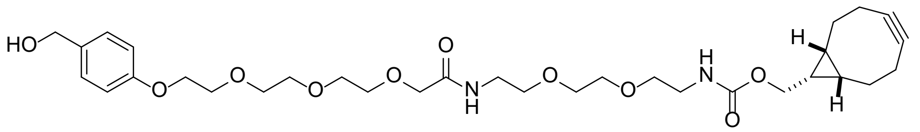

DIPEA (54 mg, 72 µL, 415 µmol) was added to a solution of 2-(2-(2-(2-(4-(hydroxymethyl)phenoxy)ethoxy)ethoxy)ethoxy)acetic acid (75 mg, 87%w, 208 µmol) and 2-(3*H*-[1,2,3]triazolo[4,5-b]pyridin-3-yl)-1,1,3,3-tetramethylisouronium hexafluorophosphate(V) (95 mg, 249 µmol) in DMF (1 mL) and the reaction was stirred for 5 mins at r.t.. To this, a solution of (*rac*-(1*R*,8*S*,9*s*)-bicyclo[6.1.0]non-4-yn-9-yl)methyl (2-(2-(2-aminoethoxy)ethoxy)ethyl)carbamate (94 mg, 291 µmol) in DMF (0.5 mL) was added and the reaction was stirred at r.t. for a further 2 h. The reaction mixture was directly purified by Prep-HPLC (EZPrep, formic modified, 20-70% acetonitrile/water) and the appropriate fractions were combined and concentrated under reduced pressure. LCMS analysis indicated that some of the product had esterified with the formic acid upon concentration. The residue was re-purified by flash-column chromatography (Redi-sep silica, 12 g, 0-25% ethanol/ethyl acetate) and the appropriate fractions were combined and concentrated under reduced pressure collecting both the desired product and the ester by-product. These samples were then dried under vacuum to

afford, **37**, (*rac*-(1*R*,8*S*,9*s*)-bicyclo[6.1.0]non-4-yn-9-yl)methyl (20-(4-(hydroxymethyl)phenoxy)-10-oxo-3,6,12,15,18-pentaoxa-9-azaicosyl)carbamate (40 mg, 32 µmol, 16 %, 50% Purity with unknown, but related, impurities) as a colourless oil

^{1}H NMR (400 MHz, $CDCl_3$, 303 K) δ (ppm) = 7.33 - 7.25 (m, 2H), 7.25 - 7.14 (m, 1H), 6.94 - 6.87 (m, 2H), 4.61 (s, 2H), 4.14 (dd, *J* = 4.4, 4.2 Hz, 4H), 4.02 - 3.99 (m, 1H), 3.96 (s, 2H), 3.89 - 3.83 (m, 2H), 3.76 - 3.66 (m, 9H), 3.61 - 3.44 (m, 11H), 3.40 - 3.30 (m, 2H), 2.44 - 2.08 (m, 5H), 1.71 - 1.47 (m, 2H), 1.36 (dt, *J* = 17.2, 8.5 Hz, 1H), 0.98 - 0.82 (m, 2H) consistent with minimum 50% purity containing unknown impurities. **^{13}C NMR** (101 MHz, $CDCl_3$, 303 K) δ (ppm) = 170.1, 158.3, 156.8, 133.7, 128.6, 114.6, 98.8, 70.9, 70.8, 70.6, 70.5, 70.3, 70.2, 69.8, 67.5, 64.9, 40.8, 38.6, 29.0, 21.4, 20.1, 17.8. 4 missing signals, suspected to be due to PEG signals overlapping. **LCMS**: Formic, RT = 0.95 min, [M+H]+ = 621.2, 49% purity by UV-Vis, multiple minor but unidentifiable impurities present. **IR** (ATIR, cm-1): 3336, 2914, 2869, 1711, 1662, 1611, 1533, 1511, 1456, 1350, 1244, 1102. **HRMS (ESI)** molecular formula ($C_{32}H_{48}N_2O_{10}$) *m/z* found $[M+H]^+$ 621.3381, $C_{32}H_{49}N_2O_{10}^+$ requires 621.3387.

(*rac*-(1*R*,8*S*,9*s*)-Bicyclo[6.1.0]non-4-yn-9-yl)methyl (20-(4-((((4-nitrophenoxy)carbonyl)-oxy)methyl)phenoxy)-10-oxo-3,6,12,15,18-pentaoxa-9-azaicosyl)carbamate 7

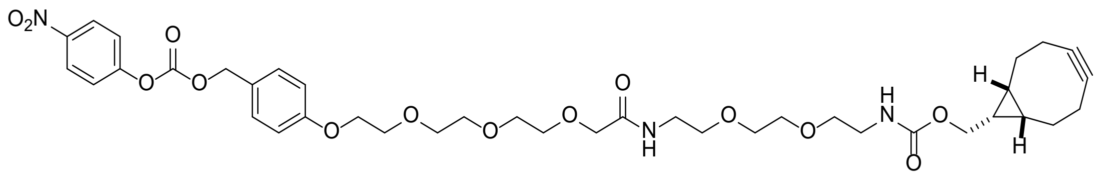

Bis(*p*-nitrophenyl) carbonate (29 mg, 97 µmol) was added to a solution of ((1*R*,8*S*,9*s*)-bicyclo[6.1.0]non-4-yn-9-yl)methyl (20-(4-(hydroxymethyl)phenoxy)-10-oxo-3,6,12,15,18-pentaoxa-9-azaicosyl)carbamate (40 mg, 50% Wt, 32 µmol) and DIPEA (17 mg, 22 µL, 0.13 mmol) in DCM (1 mL) and the reaction was stirred at r.t. for 2 h. The reaction mixture was directly purified by flash-column chromatography (Redi-Sep silica, 12 g, 0-10% ethanol/ethyl acetate) and the appropriate fractions were combined and concentrated under reduced pressure. This was then dried under vacuum to afford (*rac*-(1*R*,8*S*,9*s*)-bicyclo[6.1.0]non-4-yn-9-yl)methyl (20-(4-((((4-nitrophenoxy)carbonyl)oxy)methyl)-phenoxy)-10-oxo-3,6,12,15,18-pentaoxa-9-azaicosyl)carbamate **7** (7 mg, 6 µmol, 20 %, 70% Purity) as a pale yellow gum.

^{1}H NMR (400 MHz, $CDCl_3$, 303 K) δ (ppm) = 8.31 - 8.26 (m, 2H), 7.44 - 7.35 (m, 4H), 7.21 (br s, 1H), 6.98 - 6.93 (m, 2H), 5.35 - 5.23 (m, 3H), 4.20 - 4.14 (m, 4H), 4.03 (s, 2H), 3.90 - 3.86 (m, 2H), 3.78 - 3.74 (m, 2H), 3.73 - 3.69 (m, 6H), 3.64 - 3.49 (m, 12H), 3.42 - 3.35 (m, 2H), 2.35 - 2.19 (m, 6H), 1.66 - 1.53 (m, 2H), 1.42 (br s, 1H), 1.41 - 1.34 (m, 1H), 1.00 - 0.92 (m, 2H). consistent with ~70% purity, unknown aliphatic signals present. **^{13}C NMR** (101 MHz, $CDCl_3$, 303 K) δ (ppm) = 170.1, 159.4, 156.8, 155.6, 152.4, 145.4, 130.6, 126.6, 125.3, 121.8, 114.8, 98.8, 71.0, 70.9, 70.8, 70.63, 70.58, 70.4, 70.3, 70.19, 70.17, 69.8, 69.7, 67.5, 62.8, 40.9, 38.6, 29.1, 21.4, 20.1, 17.8. **LCMS**: formic, RT = 1.23 min, $[M+H]^+$ = 786.2, 70% purity by UV-Vis. **IR** (ATIR, cm-1): 3346, 2918, 2869, 1764, 1712, 1671, 1615, 1592, 1522, 1455, 1347, 1245, 1208, 1106. **HRMS (ESI)** molecular formula($C_{39}H_{51}N_3O_{14}$) *m/z* found $[M+H]^+$ 786.3440, $C_{39}H_{52}N_3O_{14}^+$ requires 786.3444.

4. Methionine-targeting bioconjugation

a. Preparation of methionine conjugation compound 1

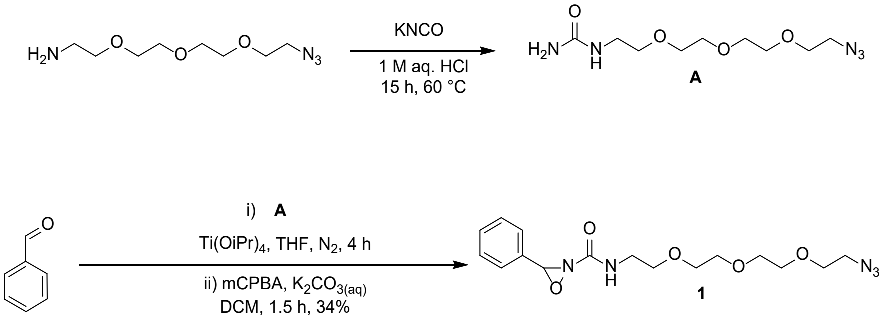

Scheme SI-4. Synthesis of oxaziridine analogue **1,** prepared adapting the protocol previously reported (**Scheme SI-4**).[8]

1-(2-(2-(2-(2-Azidoethoxy)ethoxy)ethoxy)ethyl)urea

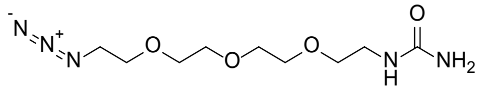

Potassium isocyanate (1.9 g, 23 mmol) was added to a solution of 2-(2-(2-(2-azidoethoxy)ethoxy)ethoxy)ethan-1-amine (1.0 g, 4.6 mmol) in 1M aqueous hydrogen chloride solution (5 ml, 5.0 mmol). The reaction mixture was stirred for 15 h at 60 °C. The reaction was cooled to r.t. before being diluted with and water (5 mL). This was then extracted with ethyl acetate (5 x 100 mL) and the organic phases were collected and dried over magnesium sulfate. This was then filtered and concentrated under reduced pressure and the residue was purified by flash column chromatography (0-25% ethanol/ethyl acetate). The appropriate fractions were combined and concentrated in vacuo to afford 1-(2-(2-(2-(2-azidoethoxy)ethoxy)ethoxy)ethyl)urea (720 mg, 2.8 mmol, 60 % yield) as a colourless oil.

1H NMR (400 MHz, CD_3SOCD_3, 303 K) δ (ppm) = 5.93 (br s, 1H), 5.55 - 5.28 (m, 2H), 3.63 - 3.59 (m, 2H), 3.59 - 3.49 (m, 10H), 3.42 - 3.36 (m, 2H), 3.12 (t, J = 5.7 Hz, 2H). 13C NMR (101 MHz, CD_3SOCD_3, 303 K) δ (ppm) = 159.1, 70.6, 70.29, 70.28, 70.17, 70.0, 69.7, 50.5, 1 signal missing, likely underneath solvent signal, IR (ATIR, cm-1): 3354 (br.), 2870, 2099, 1652, 1608, 1548, 1444, 1344, 1286, 1092. HRMS (ESI) molecular formula ($C_9H_{19}N_5O_4$) m/z found [M+H]+ 262.1519, $C_9H_{20}N_5O_4^+$ requires 262.1515

N-(2-(2-(2-(2-azidoethoxy)ethoxy)ethoxy)ethyl)-3-phenyl-1,2-oxaziridine-2-carboxamide 1

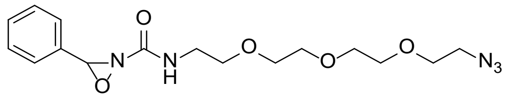

A flask was sealed and evacuated before being heated with a heat gun in an attempt to dry the flask. It was left to cool under vacuum before being backfilled with nitrogen. The reaction vessel was then evacuated and back filled with nitrogen 3 times. The dried flask was charged with a solution of 1-(2-(2-(2-(2-azidoethoxy)ethoxy)ethoxy)ethyl)urea (500 mg, 1.9 mmol) in tetrahydrofuran (6 mL), followed by benzaldehyde (0.23 mL, 2.3 mmol) and the reaction was stirred for 5 minutes under nitrogen.

Titanium(IV) isopropoxide (0.79 mL, 2.70 mmol) was then add dropwise to the reaction mixture over 5 minutes and the reaction was stirred at r.t., under nitrogen, for 4 h. The reaction mixture was concentrated under reduced pressure and the residue was redissolved in DCM (4 mL). This was then added dropwise over 5 minutes to a suspension of mCPBA (1.3 g, 5.7 mmol) in DCM (6 mL) and sat. aqueous potassium carbonate solution (8 mL) that had been pre-stirring for 10 minutes. The reaction was stirred at r.t., under nitrogen for 1.5 h. The reaction mixture was then diluted with water (100 mL) and was extracted with ethyl acetate (4 x 100 mL). The organic phases were combined and then dried over magnesium sulfate. This was then filtered and then concentrated under reduced pressure to afford a yellow oil. The residue was purified by flash column chromatography (0-100% ethyl acetate/cyclohexane). The appropriate fractions were combined and concentrated in vacuo to afford N-(2-(2-(2-(2-azidoethoxy)ethoxy)ethoxy)ethyl)-3-phenyl-1,2-oxaziridine-2-carboxamide **1** (237 mg, 0.65 mmol, 34 % yield) as a colourless oil.

1H NMR (400 MHz, CDCl3, 303 K) δ (ppm) = 7.52 - 7.38 (m, 5H), 6.60 (br s, 1H), 5.04 (s, 1H), 3.72 - 3.61 (m, 12H), 3.56 - 3.45 (m, 2H), 3.38 (t, J = 5.1 Hz, 2H). 13C NMR (101 MHz, CDCl3, 303 K) δ (ppm) = 162.4, 132.5, 130.9, 128.6, 127.9, 79.2, 70.7, 70.66, 70.64, 70.4, 70.0, 69.3, 50.7, 40.3 LCMS high pH, rt = 0.99 mins, $[M+H]^+$ = 366.0, IR (ATIR, cm-1): 3322, 2868, 2097, 1708, 1515, 1460, 1402, 1348, 1248, 1103. HRMS (ESI) molecular formula ($C_{16}H_{23}N_5O_5$) m/z found $[M+H]^+$ 366.1785, $C_{16}H_{24}N_5O_5^+$ requires 366.1772

b. Methionine bioconjugation to several antibodies

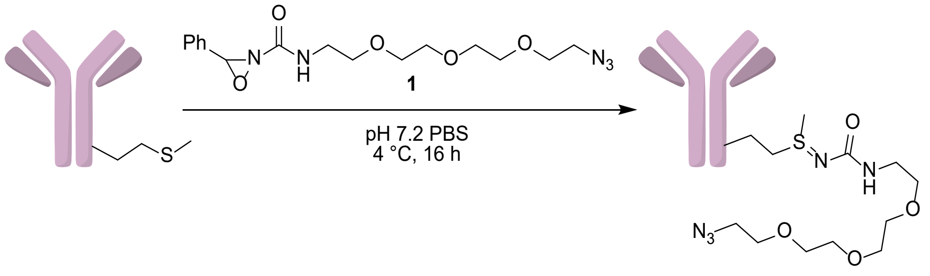

Scheme SI-5. Methionine-targeted bioconjugation using reagent **1** to install an azide

Work by Toste *et al.* reported optimal conditions for the functionalisation of native Fab and sequence modified antibodies based on αHER2.[9] Using reagent **1**, we explored bioconjugation of 3 mAbs (αIL4, αHER2, Het), each containing 12 methionine residues in their primary sequence. The influence of reagent stoichiometry was also investigated against all three proteins.

Procedure:

Oxaziridine **1** (7 or 10 eq. as 10 mM in acetonitrile) was added to **αIL4**, **αHER2**, or **Het** solution (25 µL, 3 mg/mL, 20 µM in pH 7.2 PBS) and the reaction was incubated at 4 °C for 18 or 20 h.

After this time the reaction was diluted with PBS (minimum 2-fold dilution) and purified by ultra-filtration (30 kDa, 4 x 10000 rcf, r.t., 4 min). The samples were analysed by UV-Vis to determine the protein concentration, and by mass spectrometry.

Antibody	Stoichiometry 1 (eq.)	aDAR	DAR range (%)
αIL4	7	2.47	1 (8.1), 2 (47.4), 3 (33.5), 4 (11.0)
αHER2	10	2.73	1 (3.1), 2 (38.3), 3 (41.0), 4 (17.7)
Het	7	2.45	1 (11.9), 2 (42.7), 3 (33.5), 4 (11.9)

Table SI-2. aDAR values and DAR ranges for the three investigated antibodies (repeated)

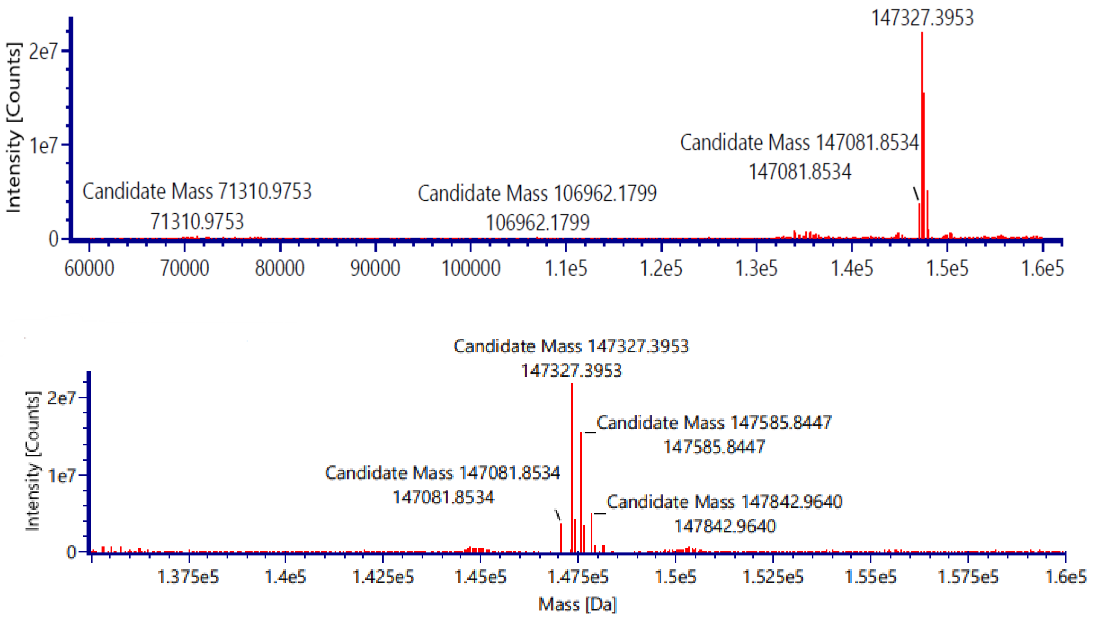

Figure SI-1. Methionine functionalised **αIL4** (7 eq of reagent **1**)

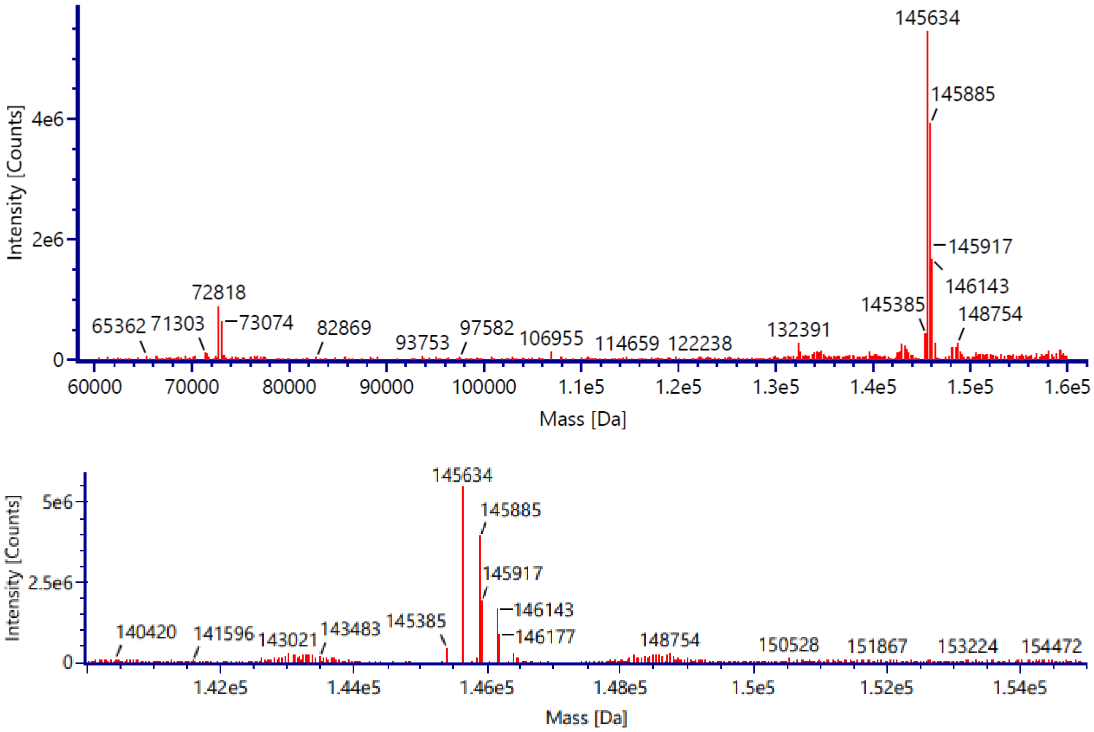

Figure SI-2. Methionine functionalised **αHER2** (10 eq of reagent **1**)

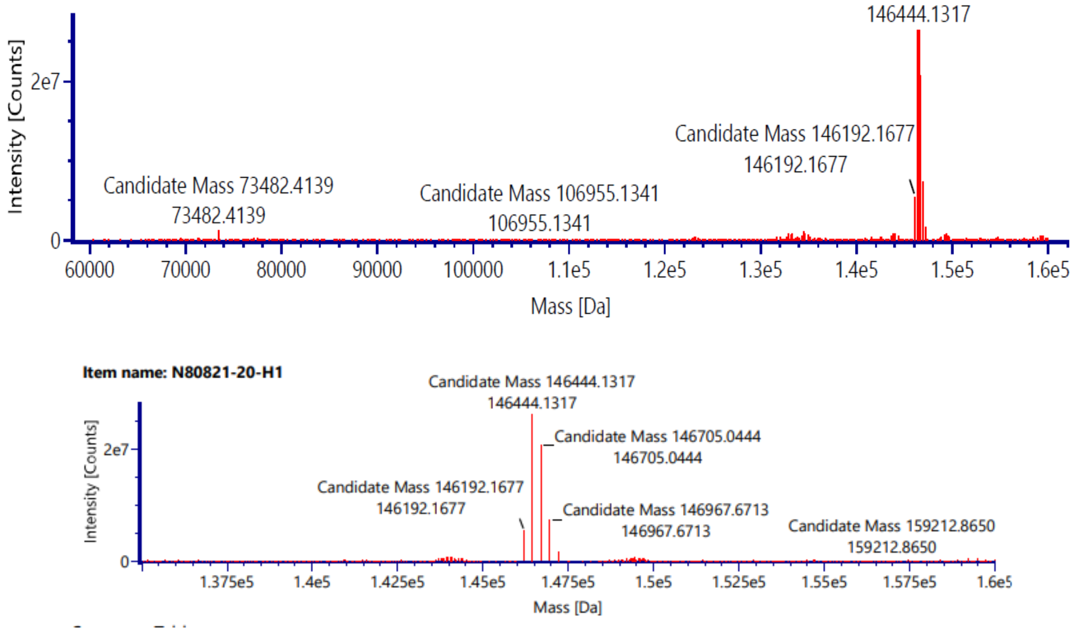

Figure SI-3. Methionine functionalised **Het** (7 eq of reagent **1**)

5. Tyrosine-targeting bioconjugation

a. Synthesis of tyrosine conjugation reagent 2

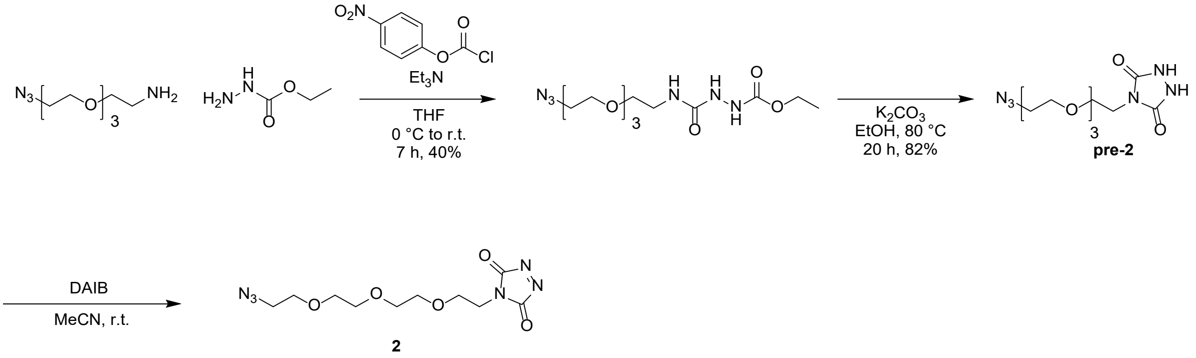

Scheme SI-6. Synthesis of triazoledione **2** prepared using a modified literature protocol.[10] DAIB= (Diacetoxyiodo)benzene

Ethyl 16-azido-4-oxo-8,11,14-trioxa-2,3,5-triazahexadecanoate

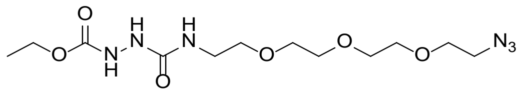

A solution of 4-nitrophenyl chloroformate (1.0 g, 5.0 mmol) in THF (16 mL) was cooled to 0 °C in an ice bath. To this, ethyl hydrazinecarboxylate (0.5 g, 5.0 mmol) and triethylamine (2.1 mL, 15 mmol) were added, and the reaction was stirred at r.t. for 1 h. After this time, a solution of 2-(2-(2-(2-azidoethoxy)ethoxy)ethoxy)ethan-1-amine (1.2 g, 5.5 mmol) in THF (4 mL) was added and the reaction was stirred at r.t. for 6 h. The reaction mixture was diluted with water (75 mL) and then extracted with ethyl acetate (4 x 100 mL). The organic phase was then dried over magnesium sulfate, filtered, and concentrated under reduced pressure. The residue was purified by flash column chromatography (0-

15% ethanol/ethyl acetate). The appropriate fractions were combined and concentrated under reduced pressure to afford ethyl 16-azido-4-oxo-8,11,14-trioxa-2,3,5-triazahexadecanoate, (690 mg, 2.0 mmol, 40 % yield) as a pale yellow oil.

1H NMR (400 MHz, CD_3SOCD_3, 303 K) δ (ppm) = 8.73 (br s, 1H), 7.74 (s, 1H), 6.25 (br s, 1H), 4.03 (q, *J* = 7.1 Hz, 2H), 3.63 - 3.48 (m, 10H), 3.44 - 3.35 (m, 4H), 3.16 (q, *J* = 6.0 Hz, 2H), 1.18 (t, *J* = 7.1 Hz, 3H). **^{13}C NMR** (101 MHz, CD_3SOCD_3, 303 K) δ (ppm) = 158.6, 157.3, 70.28, 70.27, 70.17, 70.0, 69.7, 60.8, 50.5, 39.5, 15.0, 1 signal missing, likely that 1 peak accounts for two ^{13}C. **IR**(ATIR, cm^{-1}): 3300, 2870, 2100, 1730, 1666, 1543, 1346, 1215, 1094, 1048, 935, 828, 766, 556.

Consistent with previously reported analytical data.[10]

4-(2-(2-(2-(2-Azidoethoxy)ethoxy)ethoxy)ethyl)-1,2,4-triazolidine-3,5-dione pre-2

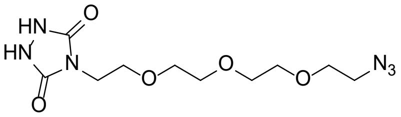

Potassium carbonate (595 mg, 4.3 mmol) was added to a solution of ethyl 16-azido-4-oxo-8,11,14-trioxa-2,3,5-triazahexadecanoate (500 mg, 1.4 mmol) in ethanol (8 mL), the reaction vessel was sealed, and the reaction was stirred at 80 °C for 20 h. The reaction mixture was then acidified to ~pH 1 using 2 M HCl solution. This was then concentrated under reduced pressure and triturated with DCM (200 mL). The organic phase was then filtered and concentrated under reduced pressure. The residue was purified by flash column chromatography (0-25% ethanol/ethyl acetate). The appropriate fractions were combined and concentrated *in vacuo* to afford 4-(2-(2-(2-(2-Azidoethoxy)ethoxy)ethoxy)ethyl) 1,2,4-triazolidine-3,5-dione **pre-2** (354 mg, 1.2 mmol, 82 % yield) as a colourless oil.

^{1}H NMR (400 MHz, CD_3SOCD_3, 303 K) δ (ppm) = 10.02 (br s, 2H), 3.65 - 3.47 (m, 14H), 3.42 - 3.36 (m, 2H) **^{13}C NMR** (101 MHz, CD_3SOCD_3, 303 K) δ (ppm) = 155.4, 70.3, 70.2, 70.1, 69.8, 69.7, 66.9, 50.5, 38.1, **IR** (ATIR, cm^{-1}): 3185, 2871, 2099, 1768, 1684, 1465, 1348, 1285, 1092, 1012, 833, 772, 593, 556.

Consistent with previously reported analytical data.[10]

4-(2-(2-(2-(2-azidoethoxy)ethoxy)ethoxy)ethyl)-3*H*-1,2,4-triazole-3,5(4*H*)-dione 2

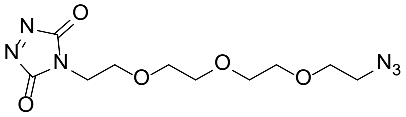

A solution of 4-(2-(2-(2-(2-azidoethoxy)ethoxy)ethoxy)ethyl)-1,2,4-triazolidine-3,5-dione (5.5 mg, 0.018 mmol) in *d*$_3$-Acetonitrile (155 µL) was added to phenyl-l3-iodanediyl diacetate (5 mg, 0.016 mmol) and the reaction was stirred at r.t. for 3 h. Full consumption of oxidant observed by ^{1}H NMR and material used without further purification to afford **2** as a 100 mM solution in *d*$_3$-acetonitrile.

^{1}H NMR (400 MHz, CD_3CN, 303 K) δ (ppm) = 3.76 - 3.49 (m, 14H), 3.41 - 3.32 (m, 2H)

b. Reagent selection and preparation

In order to better understand tyrosine conjugations, three reagents precursors, **pre-2** and **B** based on triazoledione,[11] and **C** based on *N*-methylphthalazinedione,[12] were synthesised with the aim to compare the outcome of the bioconjugation reactions for each of them.

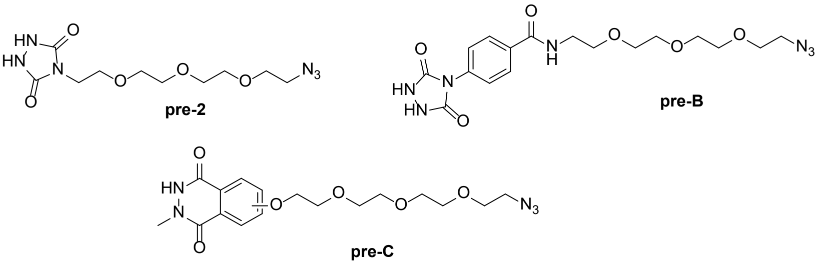

Figure SI-4. Summary of precursor compounds to be synthesised

The synthesis of **pre-2** is presented in **section 5a**. The synthesis of **pre-B** is presented in **Scheme SI-7**. Attempt at reacting oxidized **pre-B** (using *N*-bromosuccinimide/pyridine) with *p*-cresol as a model substrate the formation of the phenol carbamate produced by reaction of *p*-cresol with the isocyanate derived from decomposition of the conjugation reagent was observed. To avoid the broad reactivity of isocyanate in the bioconjugation step, this reagent was not further explored. In contrast, the oxidized **pre-2** gave, using the same protocol, the desired *p*-cresol adduct.

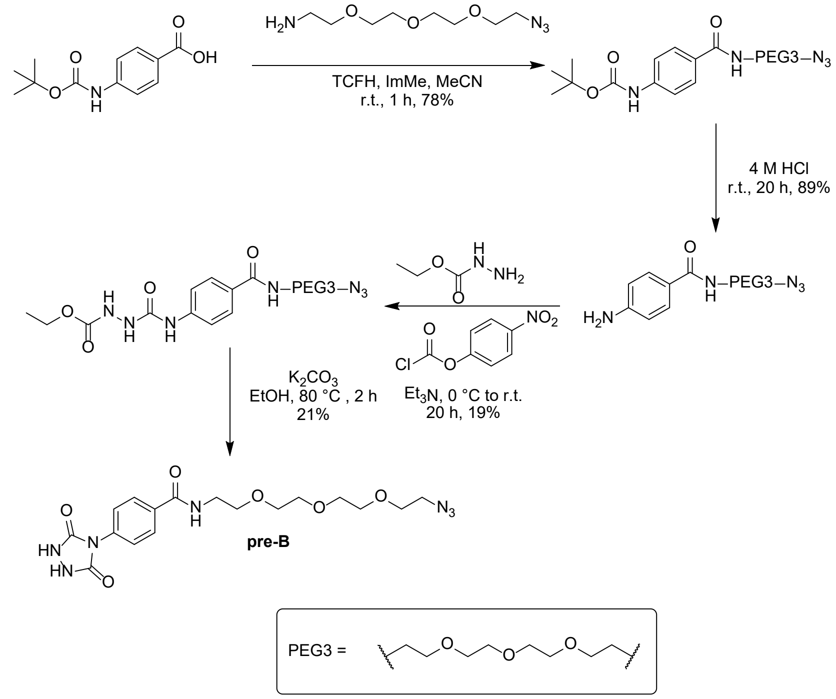

Scheme SI-7. Synthesis of N-aryl analogue **pre-B**

The luminol derivative **C** was prepared as outlined on **Scheme SI-8.** The final oxidation of **pre-C** to **C** was found to be successful using DAIB ((Diacetoxyiodo)benzene).

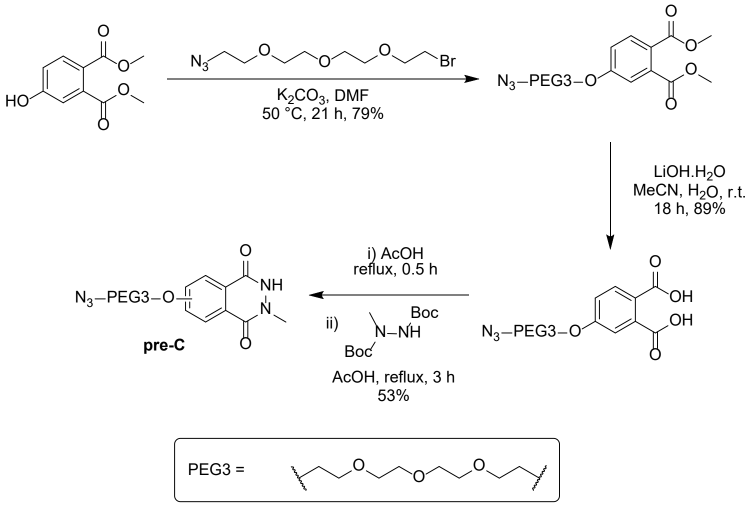

Scheme SI-8. Synthesis of PEG-linked analogue **pre-C**

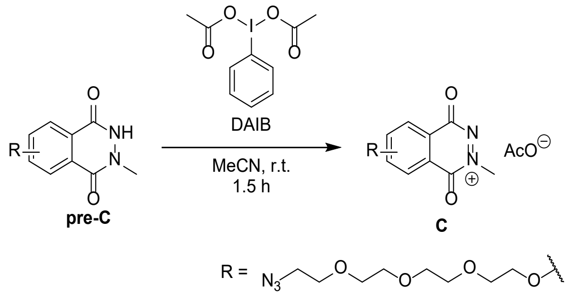

Scheme SI-9. oxidation of **pre-C** to **C**

c. Bioconjugation trial with tyrosine conjugation reagents 2 and C

With tyrosine-reactive reagents **2** and **C** in hand, bioconjugation reactions were explored with **αHER2** using 10 equivalents of reagents **2** and **C**. The outcome of this experiment favoured reagent **2** which led to an aDAR of 2.31 and 19% of unmodified antibody. In contrast, **C** led to a mixture containing a majority of unmodified antibody with satellite masses in the [M+35 - M+45] range suggesting possible oxidation and/or methylation of the biomolecules in the mixture.

Procedure: A solution of triazoledione **2,** or N-methyl phthalazinedione **C** (2 µL, 10 mM in MeCN, 10 molar eq.) was added to a solution of αHER2 (100 µL, 3 mg/mL, 20 µM in pH 7.2 PBS) and the reactions were incubated at 4 °C for 22 h. Samples were purified by ultra-filtration (30 kDa MW cut-off PES membrane, 500 µL, 4 x 5 min @ 12000 rcf, 20 °C) The samples were analysed by UV-Vis to determine the protein concentration, as well as mass spectrometry and SDS-PAGE.

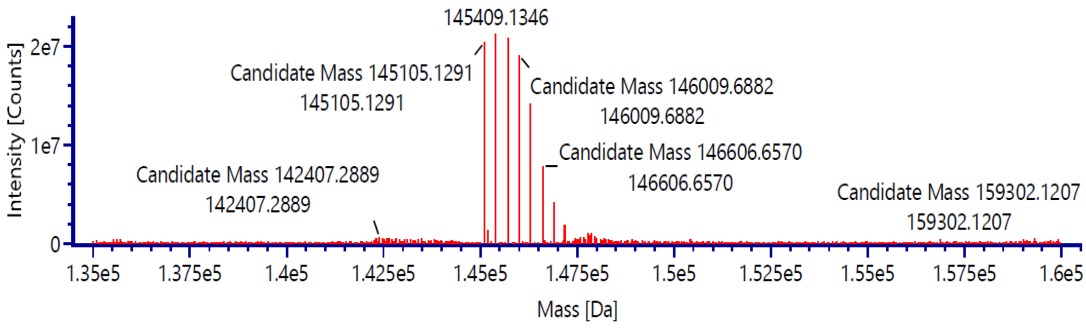

Figure SI-5. MS of **αHER2** functionalised with **2**

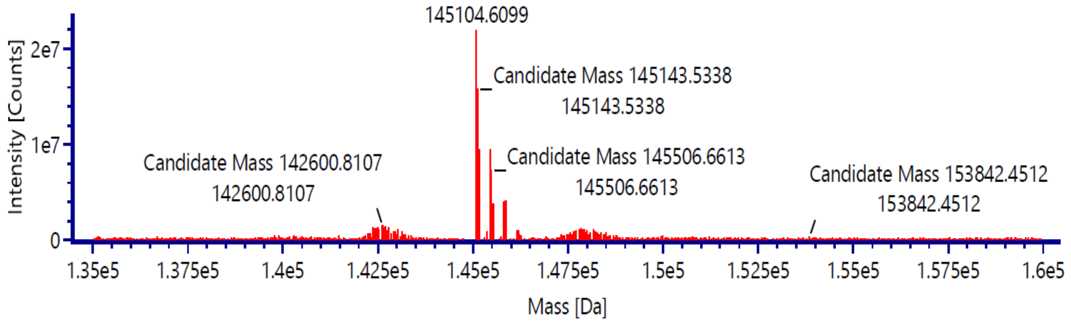

Figure SI-6. MS of **αHER2** functionalised with **C**

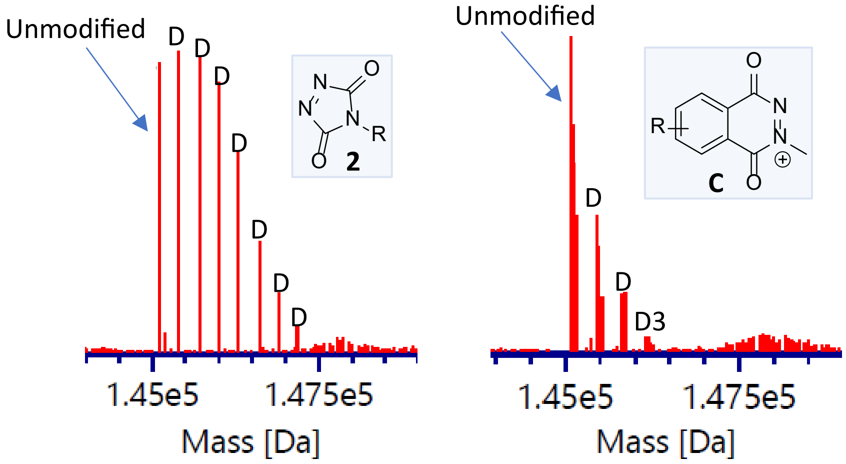

Figure SI-7. Detail of MS of functionalised **αHER2**, comparison between reagents **2** (left) and **C** (right)

d. Exploration of tyrosine-targeted bioconjugation using reagent 2

The tyrosine targeted bioconjugation using reagent **2** was applied to antibodies **αHER2**, **αIL4** and **Het** using variable stoichiometry of reagent **2** to study the impact on aDAR (**Figure SI-8**).

Procedure: using either **αIL4**, **αHER2**, or **Het** (100 µL, 3 mg/mL in pH 7.2 PBS).

- Triazoledione **2** (1 µL, 10 mM in MeCN, 5 molar eq. 1% total organic solvent in the reaction mixture) was added to antibody solution (100 µL, 3 mg/mL in pH 7.2 PBS).
- Triazoledione **2** (2 µL, 10 mM in MeCN, 10 molar eq. 2% total organic solvent in the reaction mixture) was added to antibody solution (100 µL, 3 mg/mL in pH 7.2 PBS).

- Triazoledione **2** (4 µL, 10 mM in MeCN, 20 molar eq. 3.6% total organic solvent in the reaction mixture) was added to antibody solution (100 µL, 3 mg/mL in pH 7.2 PBS).
- Triazoledione **2** (10 µL, 10 mM in MeCN, 50 molar eq. 9.1% total organic solvent in the reaction mixture) was added to antibody solution (100 µL, 3 mg/mL in pH 7.2 PBS).

Each reaction was incubated at 4 °C for 72 h. Samples were purified by Ultra-filtration (30 kDa MW cut-off PES membrane, 500 µL, 4 x 5 min @ 12000 rcf, 20 °C) and the samples were analysed by UV-Vis to determine the protein concentration, as well as mass spectrometry.

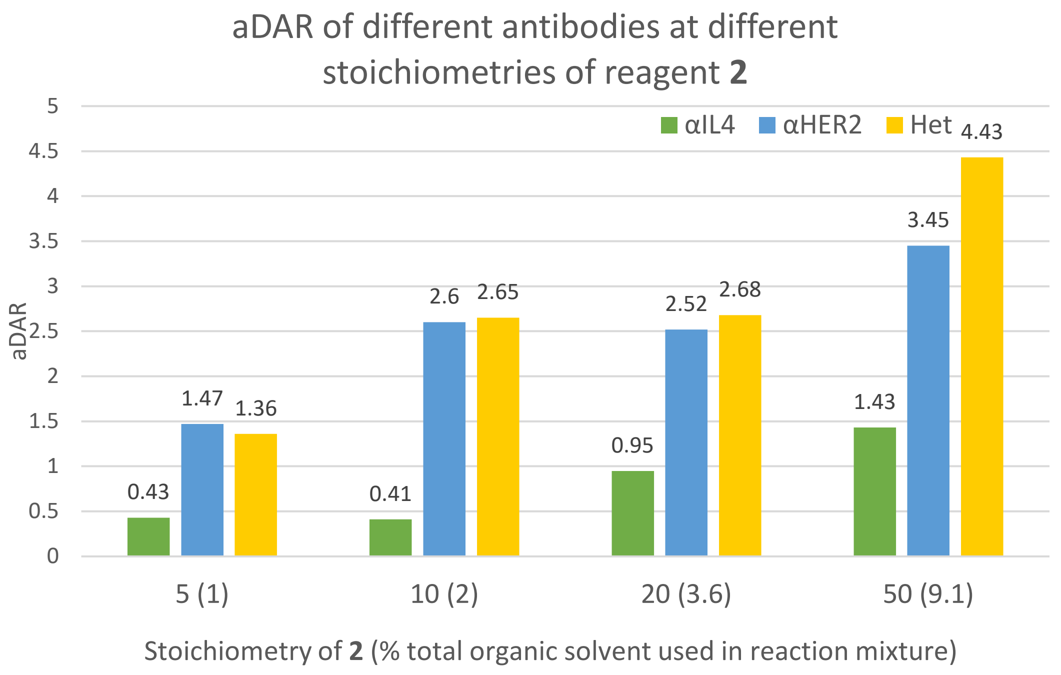

Figure SI-8. aDAR across three antibodies at set conditions (72 h, 4 °C in pH 7.2 PBS)

e. Understanding the aDAR difference between antibodies αHER2 and αIL4

A MS analysis was carried out on the post-conjugation samples after being reduced by TCEP **(Figure SI-9)**, using the same material described in the mAb cross-comparison described in the previous section) in order to understand how these conjugations differ, and to investigate if the heavy and light chains display different conjugation profiles.

αIL4 displays 20% modification to the light chain and 55% modification to the heavy chain, with only one modification observed on each chain. In comparison, **αHER2** displays almost no modification to the light chain.

To further define the general site of functionalisation for these two mAbs, a conjugation experiment was carried out on isolated Fab for both **αIL4** and **αHER2** (**Figure SI-10**).

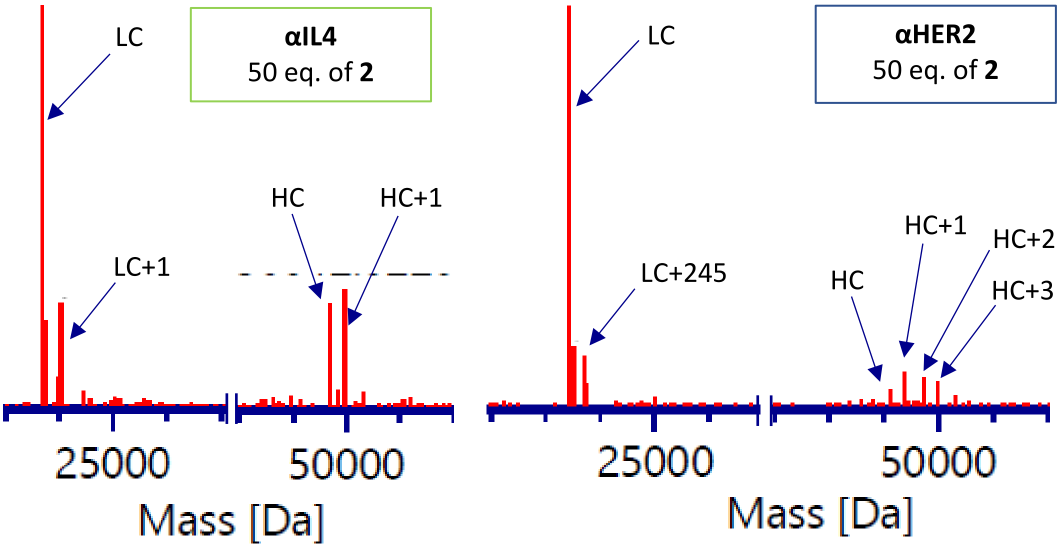

Figure SI-9. Reduced MS of **αIL4** (L) and **αHER2** (R) LC = light chain mass, HC = heavy chain mass, +x where x = 1, 2, 3 indicates the level of conjugation from reagent **2**, LC + 245 denotes an unknown species with a mass of LC + 245 Da

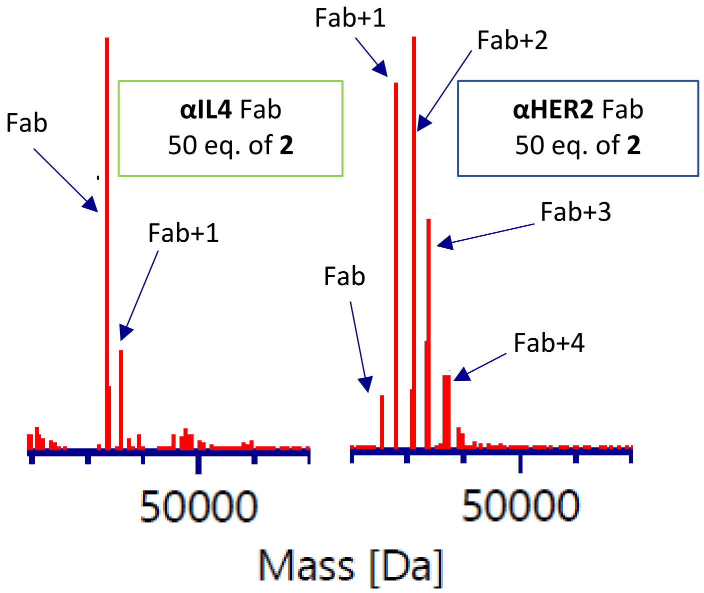

Figure SI-10. MS of **αIL4** (L) and **αHER2** (R) Fab, Fab + n indicates the level of conjugation observed for the Fab fragment using reagent **2.**

Similar to the previous results, the **αIL4** Fab showed ~20% modification with only this single modification present. This aligned closely with the degree of modification observed for the **αIL4** light chain, suggesting modification occurred on the light chain portion of the Fab. **αHER2** shows a near identical conjugation profile to what was observed in the reduced MS. It is therefore likely that these modifications occurred within the heavy chain in the Fab region of **αHER2**.

6. Re-bridging Cysteine conjugation

a. Preparation of re-bridging reagent **3**

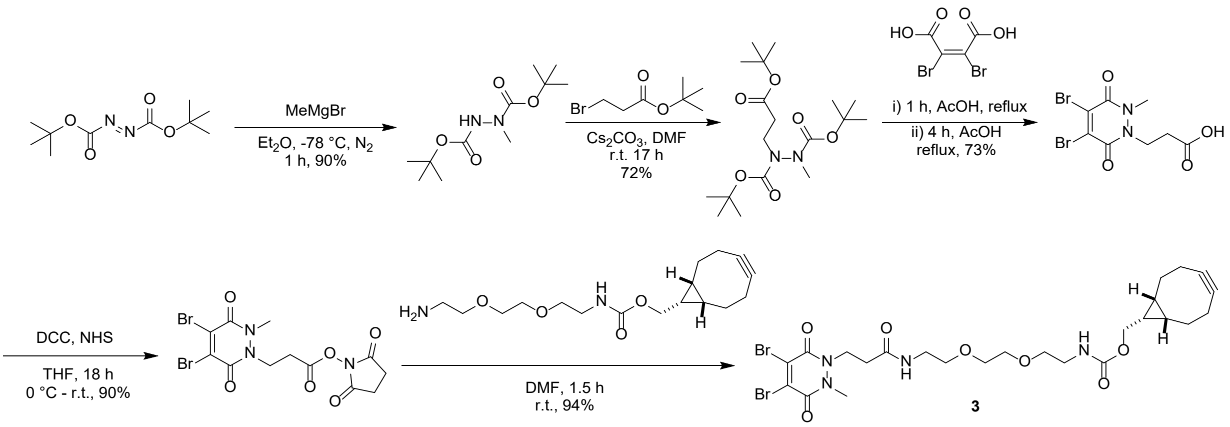

Scheme SI-10. Synthesis of Pyridazinedione-BCN analogue **3** through a modification of published literature protocols.[13]

di-*tert*-Butyl 1-methylhydrazine-1,2-dicarboxylate

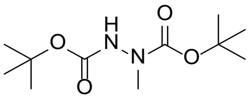

A solution of di-*tert*-butyl (*E*)-diazene-1,2-dicarboxylate (15 g, 65 mmol) in diethyl ether (200 mL) was cooled to -78 °C before 3 M methylmagnesium bromide solution in diethyl ether (30 mL, 90 mmol) was added dropwise over 20 minutes. The reaction was then stirred at -78 °C , under nitrogen for 2 h. After this time additional 3 M methylmagnesium bromide solution in diethyl ether (3 mL, 9 mmol) was added and the reaction was stirred for a further 20 minutes at -78 °C . The reaction mixture was allowed to warm to r.t. before being slowly diluted with sat. aq. ammonium chloride solution (300 mL) and diethyl ether (100 mL). The phases were separated and the aqueous was extracted with diethyl ether (2 x 300 mL) and the organic phases were combined and dried over magnesium sulfate. This was then filtered and concentrated under reduced pressure to afford a yellow oil. The residue was purified by flash column chromatography (RediSep silica, 220 g, 0-20% ethyl acetate/cyclohexane). The appropriate fractions were combined and concentrated under reduced pressure to afford a colourless oil. This was dried under vacuum to afford di-*tert*-butyl 1-methylhydrazine-1,2-dicarboxylate (14 g, 59 mmol, 90 % yield), as a white solid.

^{1}H NMR (400 MHz, $CDCl_3$, 303 K) δ (ppm) = 6.56 - 6.11 (m, 1H), 3.12 (br s, 3H), 1.74 - 1.73 (m, 1H), 1.51 - 1.39 (m, 18H). Peaks partially split due to rotamers . **^{13}C NMR** (101 MHz, $CDCl_3$, 303 K) δ (ppm) = 155.7, 81.1, 81.0, 37.5, 28.2, signal overlapping observed for both the carbonyl environments. **IR** (ATIR, cm^{-1}): 3308, 2981, 1706, 1503, 1412, 1364, 1282, 1148, 1080, 872, 852, 757, 603, 559

Consistent with previously reported data.[14]

di-*tert*-Butyl 1-(3-(*tert*-butoxy)-3-oxopropyl)-2-methylhydrazine-1,2-dicarboxylate

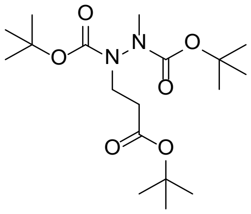

tert-Butyl 3-bromopropanoate (0.7 mL, 4.2 mmol) was added to a suspension of di-*tert*-butyl 1-methylhydrazine-1,2-dicarboxylate (680 mg, 2.8 mmol) and cesium carbonate (1.4 g, 4.1 mmol) in DMF (14 mL) and the reaction was stirred at r.t., under nitrogen for 17 h. The reaction was diluted with water (75 mL) and then extracted with ether (3 x 75 mL). All organic phases were combined, washed with 5% lithium chloride solution (2 x 25 mL) and brine (50 mL), before being dried over sodium sulfate. This was then filtered off and the filtrate was concentrated under reduced pressure. The residue was purified by flash column chromatography (0-15% ethyl acetate/cyclohexane). The appropriate fractions were combined and concentrated *in vacuo* to afford di-*tert*-butyl 1-(3-(*tert*-butoxy)-3-oxopropyl)-2-methylhydrazine-1,2-dicarboxylate (739 mg, 2.0 mmol, 72 % yield) as a colourless oil.

^{1}H NMR (400 MHz, $CDCl_3$, 303 K) δ (ppm) = 3.90 - 3.70 (m, 1H), 3.61 (s, 1H), 3.16 - 2.96 (m, 3H), 2.57 - 2.49 (m, 2H), 1.53 - 1.42 (m, 27H) **^{13}C NMR** (101 MHz, $CDCl_3$, 303 K, Multiple rotamers present) δ (ppm) = 171.2, 170.9, 155.3, 155.3, 154.3, 81.3, 81.01, 80.96, 80.9, 80.8, 80.7, 80.5, 77.2, 46.0, 44.7, 44.6, 38.2, 36.6, 34.4, 34.3, 34.1, 28.3, 28.2, 28.1 **IR** (ATIR, cm^{-1}): 2977, 2934, 1709, 1366, 1251, 1145, 1093, 848, 758.

Consistent with previously reported data.[13]

3-(4,5-Dibromo-2-methyl-3,6-dioxo-3,6-dihydropyridazin-1(2*H*)-yl)propanoic acid

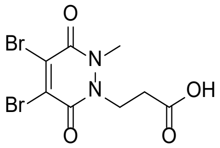

A solution of 2,3-dibromomaleic acid (1.9 g, 7.1 mmol) in acetic acid (45 mL) was heated to reflux for 1 h before di-*tert*-butyl 1-(3-(*tert*-butoxy)-3-oxopropyl)-2-methylhydrazine-1,2-dicarboxylate (2.2 g, 5.9 mmol) was added and the reaction was stirred at reflux (130 °C) for 4 h. The reaction mixture was then concentrated under reduced pressure and was immediately purified by flash column chromatography (RediSep silica 40 g, 0-25% ethanol in 1% acetic acid doped ethyl acetate). The appropriate fractions were combined and concentrated under reduced pressure and then dried under vacuum to afford 3-(4,5-dibromo-2-methyl-3,6-dioxo-3,6-dihydropyridazin-1(2*H*)-yl)propanoic acid (1.5 g, 4.3 mmol, 73 % yield) as a yellow solid.

^{1}H NMR (400 MHz, CD_3SOCD_3, 303 K) δ (ppm) = 12.46 (br s, 1H), 4.36 – 4.20 (m, 2H), 3.57 (s, 3H), 2.63 (m, 2H). **^{13}C NMR** (101 MHz, CD_3SOCD_3, 303 K) δ (ppm) = 171.8, 152.6, 152.3, 135.2, 134.9, 43.1, 34.6, 31.6. **LCMS:** formic, rt = 0.62 mins, $[M+H]^+$ = 356.6. **IR** (ATIR, cm^{-1}): 3258 (br.), 2919, 1627, 1533, 1250, 1100, 842, 557.

Consistent with previously reported analytical data.[13]

2,5-dioxopyrrolidin-1-yl 3-(4,5-dibromo-2-methyl-3,6-dioxo-3,6-dihydropyridazin-1(2*H*)-yl)propanoate

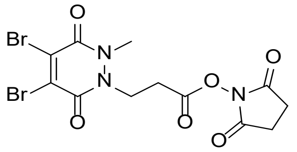

A solution of 3-(4,5-dibromo-2-methyl-3,6-dioxo-3,6-dihydropyridazin-1(2*H*)-yl)propanoic acid (500 mg, 1.4 mmol) in THF (14 mL) was cooled to 0 °C in an ice bath before DCC (319 mg, 1.5 mmol) was added and the reaction was stirred at 0 °C for 30 minutes. After this time *N*-hydroxysuccinimide (186 mg, 1.6 mmol) was added and the reaction was stirred for 18 h. The reaction mixture was filtered by vacuum, the filtrand was washed with THF (3 x 25 mL) and the filtrates were combined and concentrated under reduced pressure. The residue was purified by flash column chromatography (RediSep silica 40 g, 50-100% ethyl acetate/cyclohexane). The appropriate fractions were combined and concentrated *in vacuo* to afford 2,5-dioxopyrrolidin-1-yl 3-(4,5-dibromo-2-methyl-3,6-dioxo-3,6-dihydropyridazin-1(2*H*)-yl)propanoate (572 mg, 1.3 mmol, 90 % yield) as a white solid.

^{1}H NMR (400 MHz, CD_3SOCD_3, 303 K) δ (ppm) = 4.40 (t, *J* = 7.2 Hz, 2H), 3.58 (s, 3H), 3.18 (t, *J* = 7.2 Hz, 2H), 2.81 (s, 4H) **^{13}C NMR** (101 MHz, CD_3SOCD_3, 303 K) δ (ppm) = 170.4, 167.2, 153.2, 152.9, 136.0, 135.3, 42.5, 35.3, 29.0, 25.9 **LCMS** formic, rt = 0.70 min, $[M+H]^+$ = 453.7 **IR** (ATIR, cm^{-1}): 2934, 1802, 1774, 1732, 1616, 1567, 1290, 1200, 1190, 1091.

Consistent with previously reported analytical data.[13]

***rac*-((1R,8S,9s)-Bicyclo[6.1.0]non-4-yn-9-yl)methyl (2-(2-(2-(3-(4,5-dibromo-2-methyl-3,6-dioxo-3,6-dihydropyridazin-1(2*H*)-yl)propanamido)ethoxy)ethoxy)ethyl)carbamate 3**

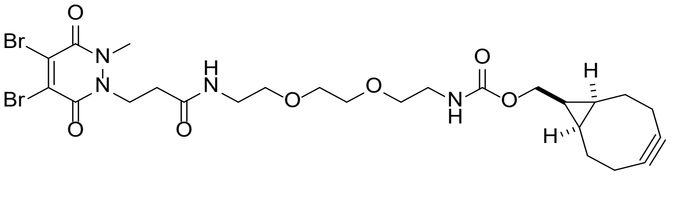

2,5-Dioxopyrrolidin-1-yl 3-(4,5-dibromo-2-methyl-3,6-dioxo-3,6-dihydropyridazin-1(2*H*)-yl)propanoate (50 mg, 0.11 mmol) was added to a solution of (1*a*,8*a*,9*b*)-Bicyclo[6.1.0]non-4-yn-9-ylmethyl *N*-{2-[2-(2-aminoethoxy)ethoxy]ethyl}carbamate (39 mg, 0.12 mmol) in DMF (0.5 mL) and the reaction was stirred at r.t. for 1.5 h. The reaction mixture was directly purified by Prep-HPLC (EZPrep, 30-35% 0.1% formic acid in acetonitrile/ 0.1% formic acid in water), **NOTE**: UV detection turned to 300 nm **ONLY** to prevent BCN breakdown and all fractions were collected) and the appropriate fractions were combined and concentrated under reduced pressure. This was then dried under vacuum to afford ((1R,8S,9s)-bicyclo[6.1.0]non-4-yn-9-yl)methyl (2-(2-(2-(3-(4,5-dibromo-2-methyl-3,6-dioxo-3,6-dihydropyridazin-1(2*H*)-yl)propanamido)ethoxy)ethoxy)ethyl)carbamate **3** (69 mg, 0.10 mmol, 94 %) as a pale yellow solid.

^{1}H NMR (400 MHz, CD_3SOCD_3, 303 K) δ (ppm) = 8.08 (br t, *J* = 5.5 Hz, 1H), 7.06 (br t, *J* = 5.1 Hz, 1H), 4.27 (t, *J* = 7.1 Hz, 2H), 4.04 (d, *J* = 8.1 Hz, 2H), 3.55 (s, 3H), 3.53 - 3.45 (m, 4H), 3.43 - 3.38 (m, 2H), 3.20 - 3.09 (m, 4H), 2.46 (t, *J* = 7.1 Hz, 2H), 2.30 - 2.10 (m, 6H), 1.62 - 1.45 (m, 2H), 1.27 (quin, *J* = 8.5 Hz, 1H), 0.93 - 0.81 (m, 2H). **^{13}C NMR** (101 MHz, CD_3SOCD_3, 303 K) δ (ppm) = 169.7, 156.9, 153.1, 152.8,

135.7, 135.4, 99.5, 70.0, 69.9, 69.6, 69.5, 61.8, 44.4, 40.6, 39.0, 35.1, 33.6, 29.1, 21.3, 20.0, 18.1. **LCMS**: Formic, RT = 0.95 min, $[M+H]^+$ = 662.9, 97% Purity by UV-Vis. **IR** (ATIR, cm^{-1}): 3323, 2914, 2865, 1706, 1627, 1538, 1440, 1396, 1248, 1100, 1013. **HRMS (ESI)** molecular formula ($C_{25}H_{34}Br_2N_4O_7$) *m/z* found $[M+H]^+$ 661.0859 , $C_{25}H_{35}N_5O_5^+$ requires 661.0867.

Consistent with previously reported analytical data.[13]

b. Evaluation of cysteine re-bridging conjugation

The cysteine re-bridging conjugation using reagent **3** was evaluated using the three antibodies used in this study (**αIL4**, **αHER2**, and **Het**) using 3 mg/mL mAb concentration. The general procedure described in the methods section of the manuscript was used.

mAb	aDAR	half-body (%)	DAR Range (%)
αIL4	3.68	3.1	3 (47.2), 4 (38.1), 5 (14.8)
αHER2	3.23	2.3	2 (4.7), 3 (67.3), 4 (28.0)
Het	3.44	2.0	3 (62.0), 4 (31.6), 5 (6.4)

Table SI-3. aDAR and DAR range across three antibodies functionalised using reagent **3.** The percentages of half-body were determined by SDS-PAGE

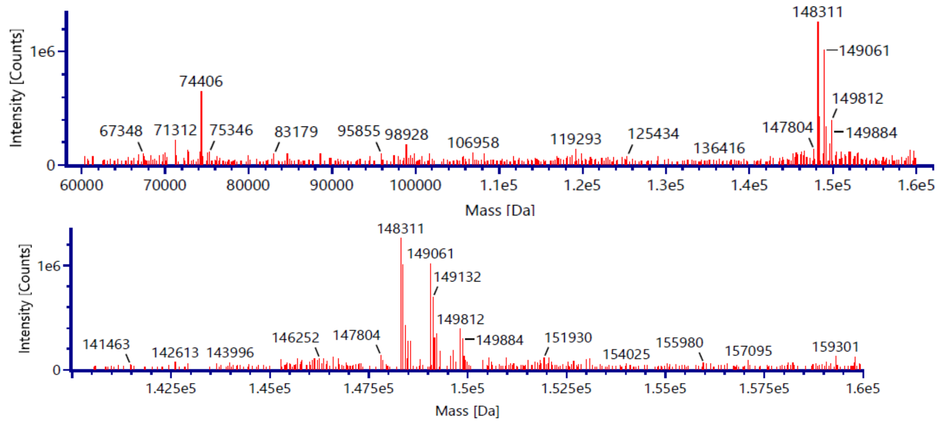

Figure SI-11. Functionalised **αIL4** using re-bridging general procedure

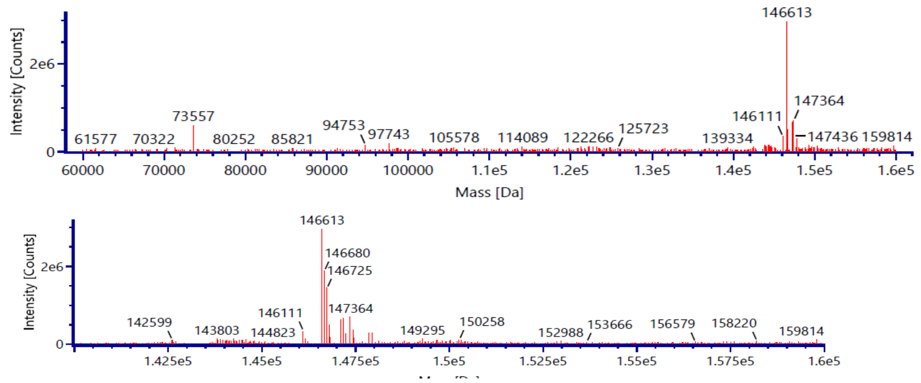

Figure SI-12. Functionalised **αHER2** using re-bridging general procedure

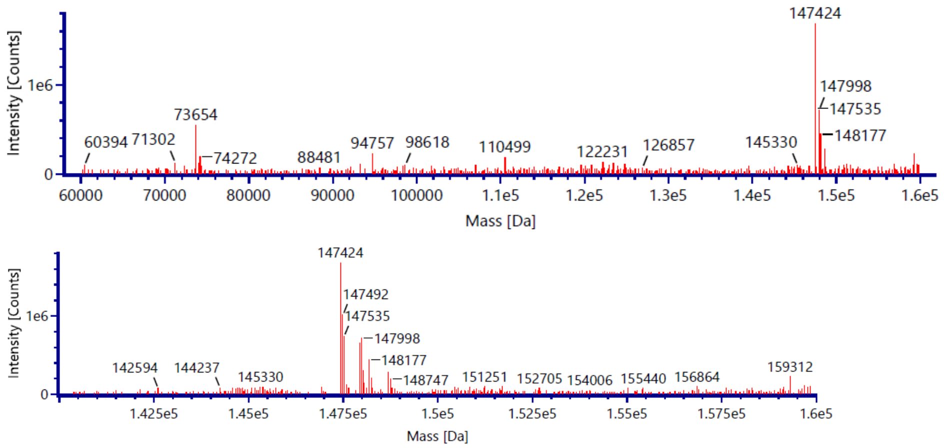

Figure SI-13. Functionalised **Het** using re-bridging general procedure

A

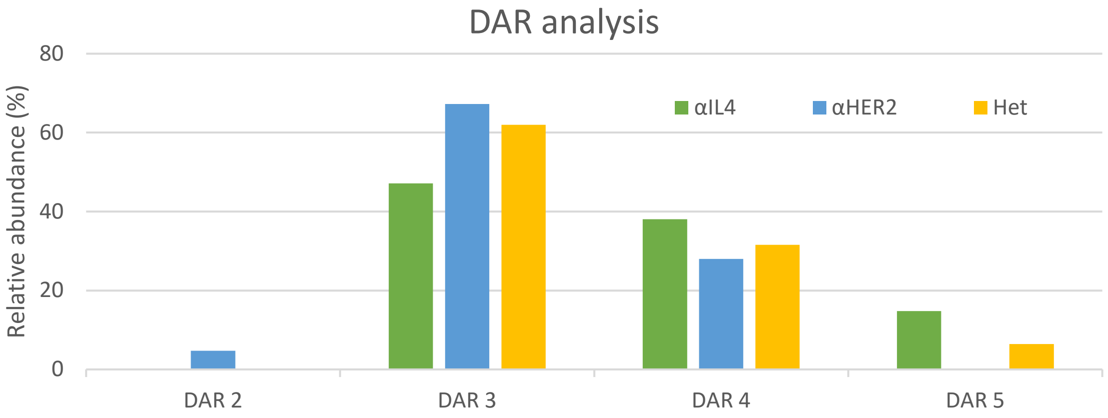

B

mAb	aDAR	half-body (%)	Yield (%)
αIL4	3.68	2.0	88.3
αHER2	3.23	2.3	76.5
Het	3.44	3.1	84.8

C

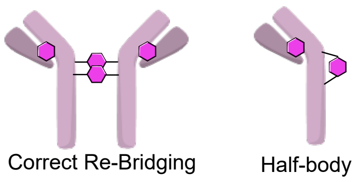

D

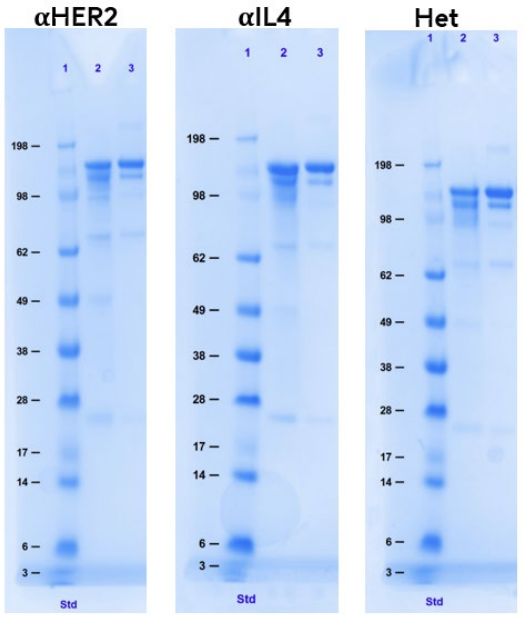

Figure SI-14. **A** Summary of conjugation results across three antibodies using re-bridging regent **3**. **B**: DAR analysis of these results, percentage of half-body was determined by SDS-PAGE. **C**: General structure of desired product and the undesired half-body by-product. **D**: SDS page of **αHER2**, **αIL4** and **Het** mAbs and conjugates using reagent **3**. 1: Ladder, 2: Unmodified Parent mAb, 3: functionalised mAb using reagent **3**

7. Lysine conjugation

a. Preparation of reagent **4**

***tert*-butyl 2-(2-(2-((methyl sulfonyl)oxy)ethoxy)ethoxy)acetate**

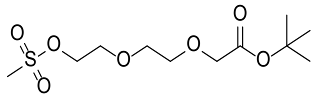

Methanesulfonyl chloride (0.13 mL, 1.7 mmol) was added dropwise over 10 mins to a cooled solution (0 °C) of *tert*-butyl 2-(2-(2-hydroxyethoxy)ethoxy)acetate (250 mg, 1.1 mmol) and DIPEA (0.20 mL, 1.1 mmol) in Diethyl ether (6 mL) and the reaction was stirred at 0 °C for 1 h under nitrogen. The reaction mixture was diluted with DCM (25 mL) and was washed with sat. aq. ammonium chloride solution (25 mL), water (25 mL), and sat. aq. sodium bicarb. solution (25 mL) and the organic phase was collected, filtered through a hydrophobic frit and then concentrated under reduced pressure. The residue was purified by flash column chromatography (RediSep silica 24 g, 0-100% ethyl acetate/cyclohexane) and the appropriate fractions were combined and concentrated *in vacuo* to afford *tert*-butyl 2-(2-(2-((methylsulfonyl)oxy)ethoxy)ethoxy)acetate (319 mg, 1.1 mmol, 94 % yield) as a pale-yellow oil.

^{1}H NMR (400 MHz, $CDCl_3$, 303 K) δ (ppm) = 4.42 - 4.36 (m, 2H), 4.00 (s, 2H), 3.83 - 3.74 (m, 2H), 3.71 (app. s, 4H), 3.08 (s, 3H), 1.48 (s, 9H) . **^{13}C NMR** (101 MHz, $CDCl_3$, 303 K) δ (ppm) = 169.4, 81.7, 70.66, 70.65, 69.2, 69.0, 69.0, 37.7, 28.1. **IR** (ATIR, cm^{-1}): 2978, 2937, 1743, 1349, 1229, 1171, 1119, 1017, 971, 917, 841, 798, 733, 527

Consistent with previously reported analytical data.[15]

***tert*-Butyl 2-(2-(2-azidoethoxy)ethoxy)acetate**

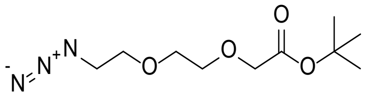

Sodium azide (65 mg, 1.0 mmol) was added to a solution *tert*-butyl 2-(2-(2-((methylsulfonyl)oxy)ethoxy)ethoxy)acetate 200 mg, 0.67 mmol) in acetonitrile (5 mL) and the reaction was stirred at r.t. for 6 h. The reaction was then heated to 60 °C for 18 h. The reaction was then concentrated under a stream of nitrogen and redissolved in ethanol (5 mL) and then stirred at 70 °C for 3 days. The reaction mixture was diluted with ethyl acetate and filtered through a 2.5 g Celite cartridge. This was then washed with ethyl acetate (3 x 10 mL) and the filtrates were concentrated under reduced pressure. The residue was purified by flash column chromatography (RediSep silica 12 g, 0-75% ethyl acetate/cyclohexane). The appropriate fractions were combined and concentrated *in vacuo* to afford *tert*-butyl 2-(2-(2-azidoethoxy)ethoxy)acetate (87 mg, 0.36 mmol, 53 % yield) as a pale yellow oil.

^{1}H NMR (400 MHz, $CDCl_3$, 303 K) δ (ppm) = 4.05 (s, 2H), 3.76 - 3.69 (m, 6H), 3.42 (t, *J* = 5.0 Hz, 2H), 1.49 (s, 9H). **^{13}C NMR** (101 MHz, $CDCl_3$, 303 K) δ (ppm) = 169.6, 81.6, 70.8, 70.7, 70.0, 69.1, 50.7, 28.1. **IR** (ATIR, cm^{-1}): 2979, 2930, 2099, 1745, 1457, 1368, 1300, 1143, 1119, 938, 843, 733.

Consistent with previously reported analytical data. [16]

2-(2-(2-Azidoethoxy)ethoxy)acetic acid

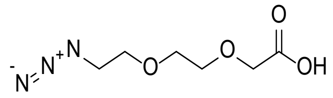

Trifluoroacetic acid (113 µL, 1.47 mmol) was added to a solution of *tert*-butyl 2-(2-(2-azidoethoxy)ethoxy)acetate (72 mg, 0.29 mmol) in DCM (1 mL) and the reaction was stirred at r.t. for 16 h. The reaction mixture was concentrated under reduced pressure and the residue was purified by flash column chromatography (RediSep silica 12 g, 0-100% ethyl acetate/cyclohexane). The appropriate fractions were combined and concentrated *in vacuo* to afford 2-(2-(2-azidoethoxy)ethoxy)acetic acid (30 mg, 0.16 mmol, 54 % yield) as a colourless oil.

^{1}H NMR (400 MHz, $CDCl_3$, 303 K) δ (ppm) = 7.77 (br. s, 1H), 4.22 (s, 2H), 3.85 - 3.77 (m, 2H), 3.77 - 3.66 (m, 4H), 3.44 (t, *J* = 5.0 Hz, 2H). **^{13}C NMR** (101 MHz, $CDCl_3$, 303 K) δ (ppm) = 173.6, 71.2, 70.4, 70.1, 68.5, 50.6. **IR** (ATIR, cm^{-1}): 3123 br., 2923, 2099, 1731, 1438, 1346, 1284, 1110, 928, 851, 670, 556.

Consistent with previously reported analytical data. [17]

2,5-Dioxopyrrolidin-1-yl 2-(2-(2-azidoethoxy)ethoxy)acetate 4

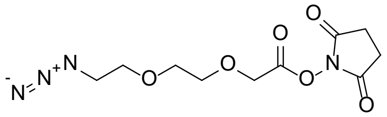

A solution of 2-(2-(2-azidoethoxy)ethoxy)acetic acid (140 mg, 740 µmol) and N,N-Dimethylformamide (12 µL, 0.15 mmol) in DCM (7 mL) was cooled to 0 °C in an ice bath under nitrogen. To this, oxalyl chloride (0.12 g, 80 µL, 0.91 mmol) was added and the reaction was stirred at 0 °C, under nitrogen, for 0.5 h before the addition of *N*-hydroxysuccinimide (128 mg, 1.1 mmol) and the reaction was stirred for a further 3 h. Disopropylethylamine (143 mg, 191 µL, 1.1 mmol) was added and the reaction was stirred, at r.t. for 15 h. The reaction mixture was concentrated under reduced pressure and purified by flash column chromatography (RediSep silica, 24 g, 50-100% ethyl acetate/cyclohexane). The appropriate fractions were combined and concentrated under reduced pressure and the residue was redissolved in DCM, transferred to a tared vial, concentrated under a stream of nitrogen and dried under vacuum to afford 2,5-dioxopyrrolidin-1-yl 2-(2-(2-azidoethoxy)ethoxy)acetate, **4**, (90 mg, 0.31 mmol, 42 %) as a colourless oil. Protocol adapted from literature,[18] no spectral data reported.

^{1}H NMR (400 MHz, $CDCl_3$, 303 K) δ (ppm) = 4.53 (s, 2H), 3.85 - 3.79 (m, 2H), 3.74 - 3.70 (m, 2H), 3.70 - 3.66 (m, 2H), 3.41 (t, *J* = 5.1 Hz, 2H), 2.85 (s, 4H). **^{13}C NMR** (101 MHz, $CDCl_3$, 303 K) δ (ppm) = 168.7, 166.0, 71.5, 70.7, 70.1, 66.7, 50.7, 25.6. **IR** (ATIR, cm^{-1}): 2925, 2101,1818, 1785, 1732, 1429, 1356, 1285, 1200, 1065, 993, 885, 852, 812, 644, 556

b. Bioconjugation procedure

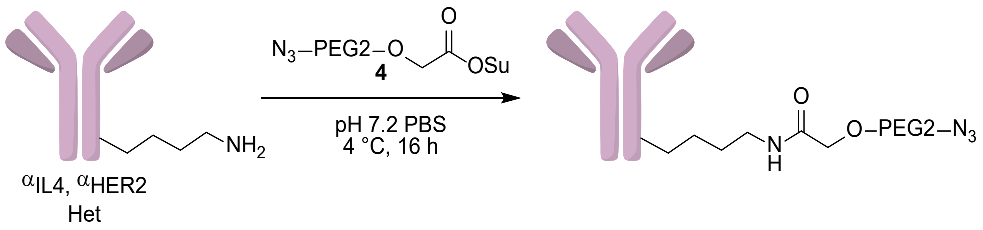

Scheme SI-11. Lysine functionalisation using NHS ester **4**

Using the general procedure described in the Methods section of the manuscript.

Antibody	Stoichiometry 4	aDAR	DAR range
**αIL4**	5	1.96	0 (10.8), 1 (27.4), 2 (28.7), 3 (21.8), 4 (11.4)
	10	4.01	1 (6.7), 2 (15.1), 3 (19.5), 4 (19.6), 5 (16.5), 6 (13.6), 7 (9.0)
**αHER2**	5	1.10	0 (34.1), 1 (32.8), 2 (22.3), 3 (10.9)
	10	2.05	0 (16.2), 1 (27.3), 2 (23.0), 3 (15.3), 4 (8.7), 5 (5.9), 6 (3.7)
**Het**	5	2.36	0 (7.4), 1 (23.0), 2 (25.4), 3 (22.0), 4 (14.4), 5 (7.7)
	10	3.84	1 (9.8), 2 (17.8), 3 (20.5), 4 (17.1), 5 (14.1), 6 (10.5), 7 (6.7), 8 (3.6)

Table SI-4. aDAR and DAR range values for lysine-functionalised antibodies using reagent **4**

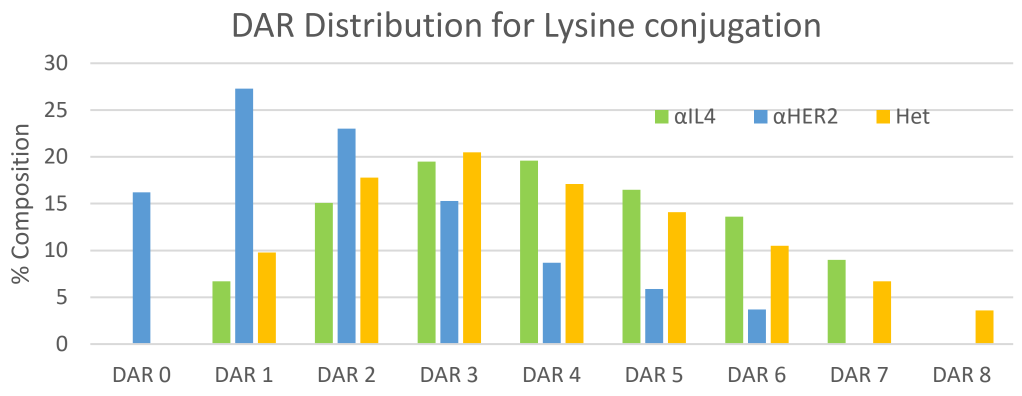

Figure SI-15. DAR distribution for three antibodies investigated at 10 molar eq. of reagent **4**

8. Library building experiments Figure 5

a. Summary table

mAb	Bioconjugation method	Linker	Payload	Yield (%)*	aDAR	ΔaDAR (fraction)	Click conversion	ΔaDAR (%)	mAb Mass	mAb Mass deglyc.	warhead W mass	Linker L Mass	Payload P Mass	WLP Mass
**αIL4**	Rebridging	none	none	88	3.7	na	na	na	149692	146806	502	na	na	Na
**αIL4**	Rebridging	VC	Biotin	88	3.4	-0.08	100.00	-8.00	149692	146806	502	706	285	1493
**αIL4**	Rebridging	VC	Fluorescein	100	ND	ND	ND	ND	149692	146806	502	706	448	1656
**αIL4**	Rebridging	VC	VHL	95	3.3	-0.09	100.00	-9.00	149692	146806	502	706	429	1637
**αIL4**	Rebridging	VA	Biotin	68	3.4	-0.08	100.00	-8.00	149692	146806	502	620	285	1407
**αIL4**	Rebridging	VA	Fluorescein	92	ND*	ND*	ND*	ND*	149692	146806	502	620	448	1570
**αIL4**	Rebridging	VA	VHL	100	3.2	-0.12	100.00	-12.00	149692	146806	502	620	429	1551
**αIL4**	Rebridging	PEG	Biotin	85	3.4	-0.08	100.00	-8.00	149692	146806	502	541	285	1328
**αIL4**	Rebridging	PEG	Fluorescein	96	3.3	-0.11	100.00	-10.81	149692	146806	502	541	448	1491
**αIL4**	Rebridging	PEG	VHL	100	3.2	-0.14	100.00	-14.00	149692	146806	502	541	429	1472
**Het**	Rebridging	none	none	85	3.4	na	na	na	148805	145919	502	na	na	Na
**Het**	Rebridging	VC	Biotin	92	3.2	-0.09	100.00	-9.00	148805	145919	502	706	285	1493
**Het**	Rebridging	VC	Fluorescein	92	ND*	ND*	ND*	ND*	148805	145919	502	706	448	1656
**Het**	Rebridging	VC	VHL	85	3.3	-0.06	100.00	-6.00	148805	145919	502	706	429	1637
**Het**	Rebridging	VA	Biotin	69	3.2	-0.08	100.00	-8.00	148805	145919	502	620	285	1407
**Het**	Rebridging	VA	Fluorescein	80	ND*	ND*	ND*	ND*	148805	145919	502	620	448	1570
**Het**	Rebridging	VA	VHL	77	3.2	-0.07	100.00	-7.00	148805	145919	502	620	429	1551
**Het**	Rebridging	PEG	Biotin	69	3.3	-0.06	100.00	-6.00	148805	145919	502	541	285	1328
**Het**	Rebridging	PEG	Fluorescein	93	3.2	-0.06	100.00	-5.88	148805	145919	502	541	448	1491
**Het**	Rebridging	PEG	VHL	89	3.1	-0.1	100.00	-10.00	148805	145919	502	541	429	1472
**αHER2**	Rebridging	none	none		3.7	na	na	na	147995	145109	502	na	na	Na
**αHER2**	Rebridging	VC	Biotin	88	3.5	-0.04	100.00	-4.00	147995	145109	502	706	285	1493
**αHER2**	Rebridging	VC	Fluorescein	100	3.4	-0.08	100.00	-8.00	147995	145109	502	706	448	1656
**αHER2**	Rebridging	VC	VHL	93	3.6	-0.04	100.00	-4.00	147995	145109	502	706	429	1637
**αHER2**	Rebridging	VA	Biotin	75	3.7	-0.01	100.00	-1.00	147995	145109	502	620	285	1407
**αHER2**	Rebridging	VA	Fluorescein	100	3.6	-0.04	100.00	-4.00	147995	145109	502	620	448	1570
**αHER2**	Rebridging	VA	VHL	85	3.7	<1%	100.00	0.00	147995	145109	502	620	429	1551
**αHER2**	Rebridging	PEG	Biotin	80	3.7	-0.01	100.00	-1.00	147995	145109	502	541	285	1328
**αHER2**	Rebridging	PEG	Fluorescein	97	3.7	-0.01	100.00	-1.00	147995	145109	502	541	448	1491
**αHER2**	Rebridging	PEG	VHL	93	3.8	0.03	100.00	3.00	147995	145109	502	541	429	1472
**αHER2**	Methionine	none	none		2.7	na	na	na	147995	145109	259	na	na	na
**αHER2**	Methionine	VC	Biotin	70	2.7	-0.03	100.00	-3.00	147995	145109	259	582	285	1126
**αHER2**	Methionine	VC	Fluorescein	54	2.4	-0.13	79.00	-13.00	147995	145109	259	582	448	1289
**αHER2**	Methionine	VC	VHL	95	2.5	-0.1	2.00	-10.00	147995	145109	259	582	429	1270
**αHER2**	Methionine	VA	Biotin	55	2.3	-0.17	24.00	-17.00	147995	145109	259	496	285	1040
**αHER2**	Methionine	VA	Fluorescein	40	2.4	-0.12	76.00	-12.00	147995	145109	259	496	448	1203
**αHER2**	Methionine	VA	VHL	92	2.4	-0.13	0.00	-13.00	147995	145109	259	496	429	1184
**αHER2**	Methionine	PEG	Biotin	83	2.6	-0.05	100.00	-5.00	147995	145109	259	647	285	1191
**αHER2**	Methionine	PEG	Fluorescein	71	2.4	-0.12	92.00	-12.00	147995	145109	259	647	448	1354
**αHER2**	Methionine	PEG	VHL	81	ND*	ND*	ND*	ND*	147995	145109	259	647	429	1335
**αHER2**	Tyrosine	none	none		4	na	na	na	147995	145109	300	na	na	na

mAb	Bioconjugation method	Linker	Payload	Yield (%)*	aDAR	ΔaDAR (fraction)	Click conversion	ΔaDAR (%)	mAb Mass	mAb Mass deglyc.	warhead W mass	Linker L Mass	Payload P Mass	WLP Mass
**αHER2**	Tyrosine	VC	Biotin	66	3.7	-0.09	100.00	-9.00	147995	145109	300	582	285	1167
**αHER2**	Tyrosine	VC	Fluorescein	67	2.9	-0.28	82.00	-28.00	147995	145109	300	582	448	1330
**αHER2**	Tyrosine	VC	VHL	99	3.1	-0.23	0.00	-23.00	147995	145109	300	582	429	1311
**αHER2**	Tyrosine	VA	Biotin	54	2.6	-0.35	10.00	-35.00	147995	145109	300	496	285	1081
**αHER2**	Tyrosine	VA	Fluorescein	72	3.1	-0.25	69.00	-25.00	147995	145109	300	496	448	1244
**αHER2**	Tyrosine	VA	VHL	98	2.49	-0.38	4.00	-37.75	147995	145109	300	496	429	1225
**αHER2**	Tyrosine	PEG	Biotin	78	3.2	-0.12	100.00	-12.00	147995	145109	300	647	285	1232
**αHER2**	Tyrosine	PEG	Fluorescein	92	4.2	0.05	31.00	5.00	147995	145109	300	647	448	1395
**αHER2**	Tyrosine	PEG	VHL	83	3.29	-0.18	9.00	-17.75	147995	145109	300	647	429	1376

Table SI-5. Summary Table of data obtained by UV-Vis and MS analysis for the library of 45 conjugates. Yields (%) were determined by UV-Vis spectroscopy. ΔDAR is the change in aDAR following the final click step click step. The click conversion was determined by integration of MS signals assigned to the conjugated species showing complete or incomplete attachment of the linker-payload fragment. na = not applicable. ND* poor signal-to-noise and/or complex mass profile prevented a satisfactory analysis of the MS data; ”WLP” = Warhead-Linker-Payload.

b. SDS-PAGE

Disulfide Re-bridging Approach

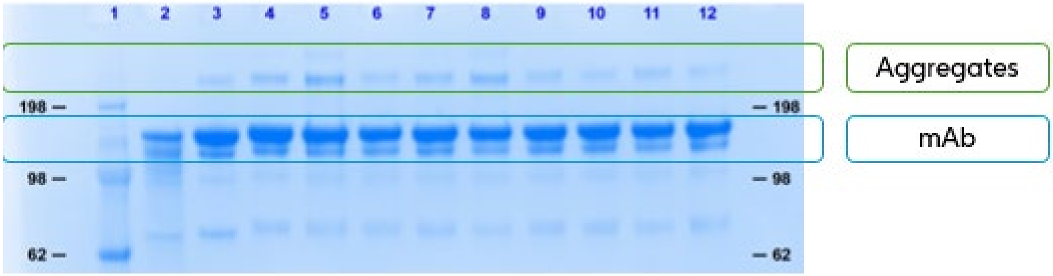

Methionine-targeted Approach

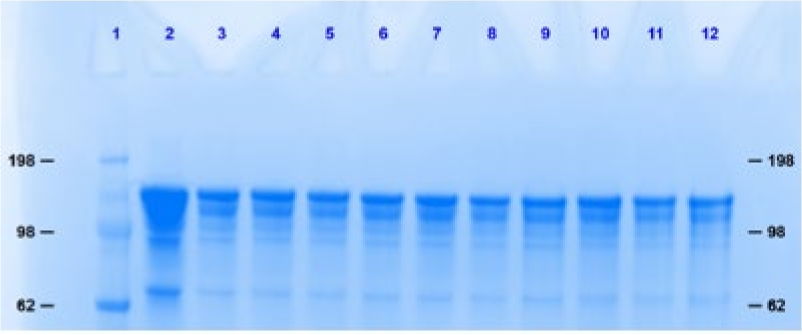

Tyrosine targeted Approach

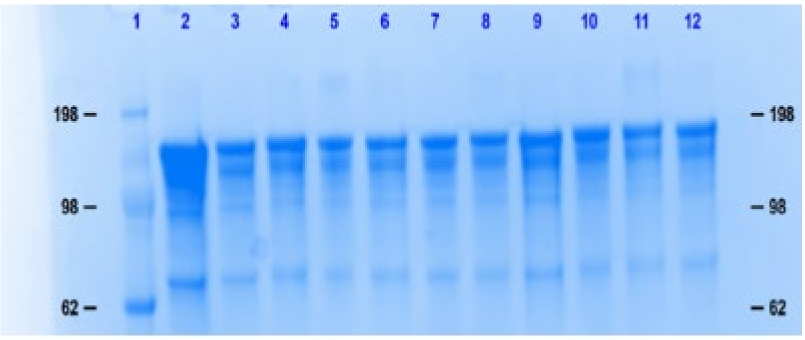

Figure SI-16. SDS-PAGE analysis of a subset of product ADCs made using **αHER2**. 1: MW Ladder, 2: unmodified **αHER2**, 3: **αHER2** functionalised with re-bridging reagent **3**, 4: + VC-Biotin, 5: +VC-Fluorescein, 6: + VC-VHL, 7: +VA-Biotin, 8: +VA-Fluorescein, 9: +VA-VHL, 10: +PEG-Biotin, 11: +PEG-Fluorescein, 12: +PEG-VHL

c. Relation between aDAR changes, click conversion and calculated LogP of the linker-payload fragment

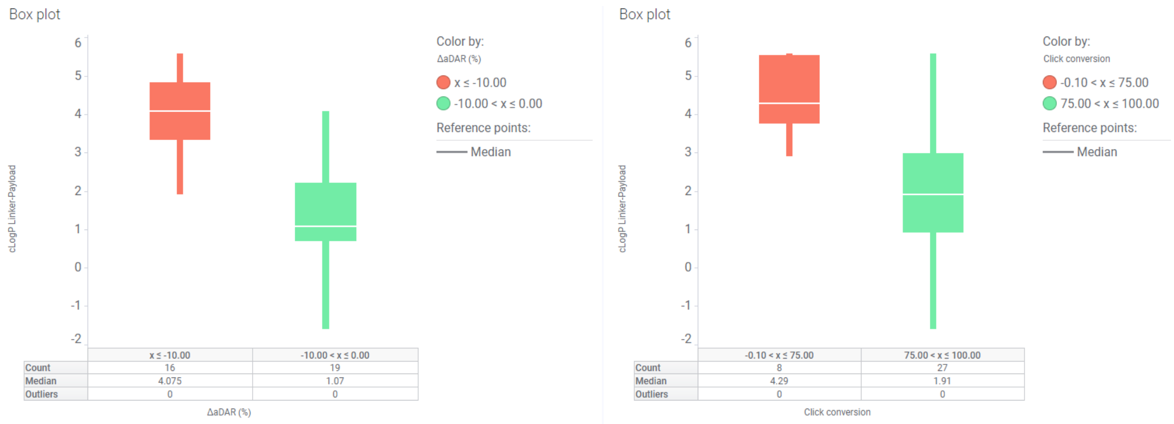

Figure SI-17. Box plots showing the cLogP of the Linker-Payload combination against the aDAR change (aΔDAR) post click step showing that larger drop in aDAR is observed for combination with higher cLogP (Left plot) and showing lower conversion in the click step is observed for combination with higher cLogP (right plot)

Payload	Linker	Click handle on Linker	cLogP
Biotin	none	none	-1.21
VHL	none	none	1.41
Fluorescein	none	none	1.45
Biotin	VC	Azide	-1.6
Biotin	VA	Azide	0.91
Biotin	PEG	Azide	0.69
VHL	VC	Azide	2.22
VHL	VA	Azide	2.5
VHL	PEG	Azide	3.47
Fluorescein	VC	Azide	1.07
Fluorescein	VA	Azide	4.08
Fluorescein	PEG	Azide	nc
Biotin	VC	BCN	1.44
Biotin	VA	BCN	2.91
Biotin	PEG	BCN	1.91
VHL	VC	BCN	4.04
VHL	VA	BCN	5.54
VHL	PEG	BCN	4.54
Fluorescein	VC	BCN	4.11
Fluorescein	VA	BCN	5.58
Fluorescein	PEG	BCN	4.58

Table SI-6. cLogP calculations for linker-payload fragments used in click step. The cLogP values of the linker-payload fragments were based on the spirocyclic isomer. VC = Valine-Citrulline, VA = Valine-Alanine, PEG = polyethylene glycol chain. The cLogP calculations were carried out using BioByte clogP model. nc = calculation failed.

9. Improving the Click conversion

Payload attachment chemistry followed the general procedure described in the Methods section of the manuscript, using linker **5,14,15,16** solution (15-50 µL, 20 mM, 10 mM in DMSO) and payload **12** solution (15-50 µL (equivalent volume), 30 mM in DMSO, 1.5 molar eq.). Reactions were stirred at r.t. for 20 h and analysed by LCMS.

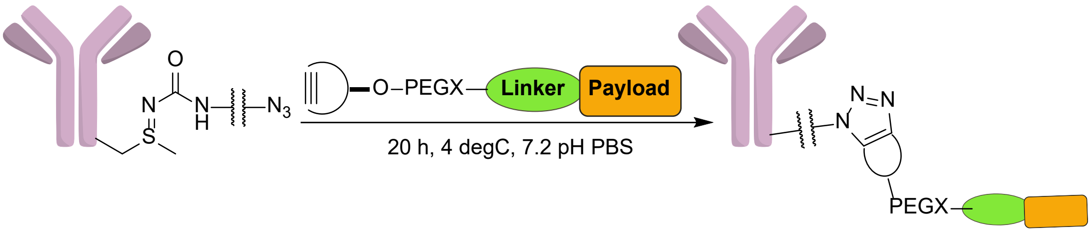

Scheme SI-12. Click chemistry to install PEG-containing linkers

Aliquots of methionine functionalised αHER2 were diluted to 2 mg/mL, 13 µL, in pH 7.2 PBS. A series of reactions were set up from this:

- SA-2PEG-VC-VHL, (1.4 µL, 10 mM 20 molar eq.) was added to a methionine-functionalised **αHER2** solution (50 µL) to provide 2.5% total organic solvent within the reaction.
- SA-4PEG-VC-VHL (2.7 µL, 10 mM, 20 molar eq.) was added to methionine-functionalised **αHER2** solution (100 µL) to provide 2.5% total organic solvent within the reaction.
- SA-6PEG-VC-VHL, (2.7 µL, 10 mM, 20 molar eq.) was added to methionine-functionalised **αHER2** solution (100 µL) to provide 2.5% total organic solvent within the reaction.
- SA-6PEG-VC-VHL,, (2.7 µL, 5 mM, 20 molar eq.) was added to methionine-functionalised **αHER2** solution (50 µL) to provide 5% total organic solvent within the reaction.
- SA-6PEG-VC-VHL, (5.4 µL, 2.5 mM, 20 molar eq.) was added to methionine-functionalised **αHER2** solution (50 µL) to provide 10% total organic solvent within the reaction.

All reactions were incubated at r.t. for 24 h. After this time each reaction was diluted to 500 µL with PBS and purified by UltraFiltration (6 x 5 min, 12000 rcf, 30 kDa MW cut-off PES membrane), after the final spin the vials were diluted to 100 µL and then transferred to a 500 µL Eppendorf vial. The samples were analysed by UV-Vis to determine the protein concentration, as well as mass spectrometry and SDS-PAGE.

EG Chain	Payload (Total org. %)	aDAR	DAR range (%)	Complete click (%)
No PEG	VHL (2.5)	2.45	1 (3.6), 2 (50.0), 3 (44.4), 4 (2.0)	2.0
PEG2	VHL (2.5)	2.66	2 (60.7), 3 (39.3), 5 (10.2)	10.4
PEG4	VHL (2.5)	2.51	2 (49.2), 3 (50.8)	20.2
PEG6	VHL (2.5)	2.69	2 (40.4), 3 (50.0), 4 (9.5)	59.0
	VHL (5)	2.66	2 (43.0), 3 (48.1), 4 (8.9)	58.1
	VHL (10)	2.76	2 (37.8), 3 (47.9), 4 (14.2)	77.0

Table SI-7. aDAR values, DAR ranges, and percentage click conversion for PEG-containing Val-Cit linkers

10. Comparison of linear and convergent approach to a Val-Cit-Biotin αIL4 ACD and a Val-Cit-Fluorescein αIL4 ACD

a. Linear approach

Reagent Synthesis:

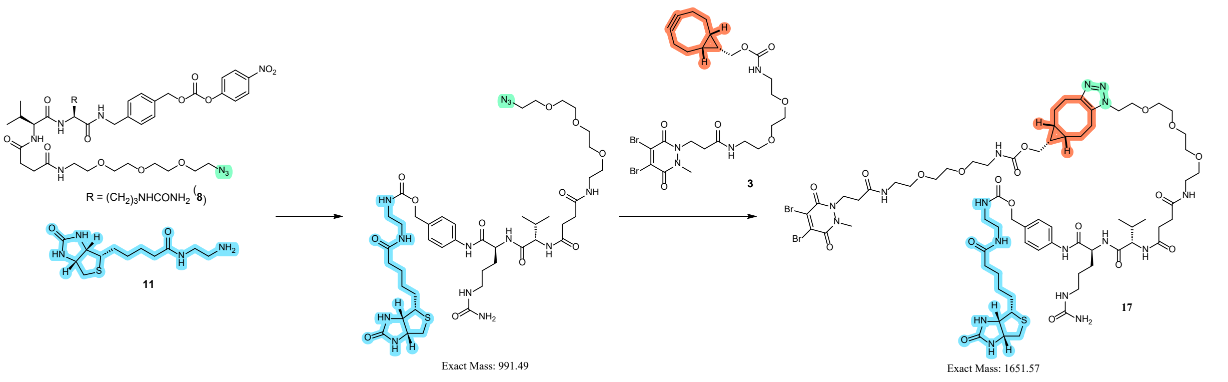

Scheme SI-13: Synthesis of to payload-bearing conjugation reagent **17**

Payload solution (**11**, 20 µL 45 mM in DMSO, 2.25 molar eq.) was added to linker **8** (20 µL, 30 mM in DMSO, 1.5 molar eq.) and the reaction was stirred at r.t. for 20 h. LCMS analysis indicated complete consumption of **8** (**Figure SI-18**, A). To this, a solution of pyridazinedione **3** (40 µL, 10 mM in DMSO) was added and the reaction was stirred for a further 3 h. LCMS analysis indicated complete consumption of **3** to produce a solution of bioreactive linker-payload fragment **17** (5 mM in DMSO). These reagents were carried forward without further purification.

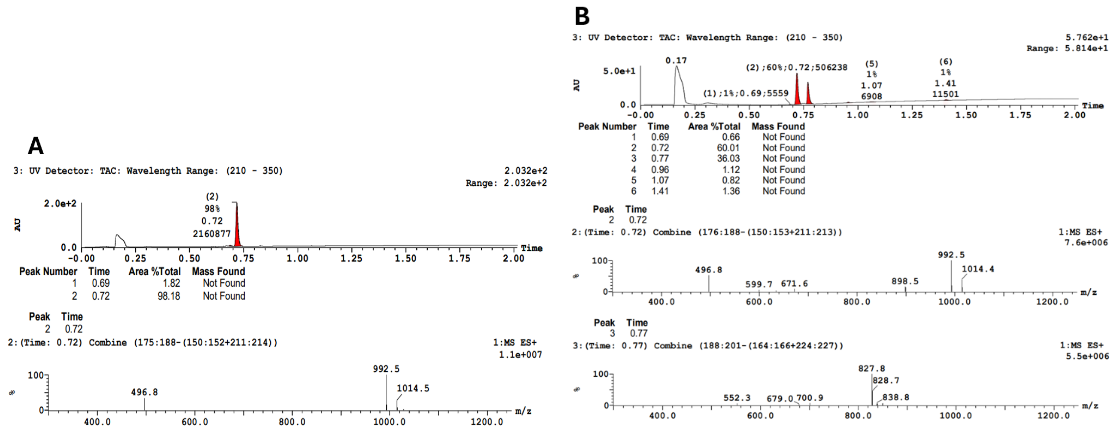

Figure SI-18: A = LCMS for the reaction of payload **11** and linker **8** LCMS for the reaction of payload **11** and linker **8** (product $C_{43}H_{69}N_{13}O_{12}S$ calc. exact mass 991.49, observed $[M+H]^+$ = 992.5); **B** = LCMS after click reaction with reagent **3** (product $C_{68}H_{103}Br_2N_{17}O_{19}S$ calc. exact mass 1653.57, observed $[M+2H]^{2+}/2$ = 827.8)

Bioconjugation Step:

A solution of pyridazinedione-linker-payload **17** (20 molar eq, 5 mM in DMSO) was added to **αIL4** (3 mg/mL, 20 µM, in pH 8.5 BBS) and the reaction was incubated at 4 °C for 1 h. After this time a solution of TCEP (10 molar eq, 10 mM in pH 8.5 BBS) and the reaction was incubated for a further 16 to 20 h.

After this time the reaction was diluted with PBS (minimum 2 fold dilution) and purified by ultra-filtration (30 kDa MW cut-off PES membrane, 500 µL, 5 x 5 min @ 12000 rcf, 20 °C). The filtrand was diluted to the nearest graduation, the value was recorded to give yield, and transferred to an

Eppendorf vial. The samples were analysed by UV-Vis to determine the protein concentration, as well as mass spectrometry and SDS-PAGE.

Payload	aDAR	DAR range (%)
Biotin (**11**) using **17**	4.06	3 (32.6), 4 (48.5), 5 (18.9), 6 (10.1)

Table SI-8. aDAR values and DAR ranges for the linear approach

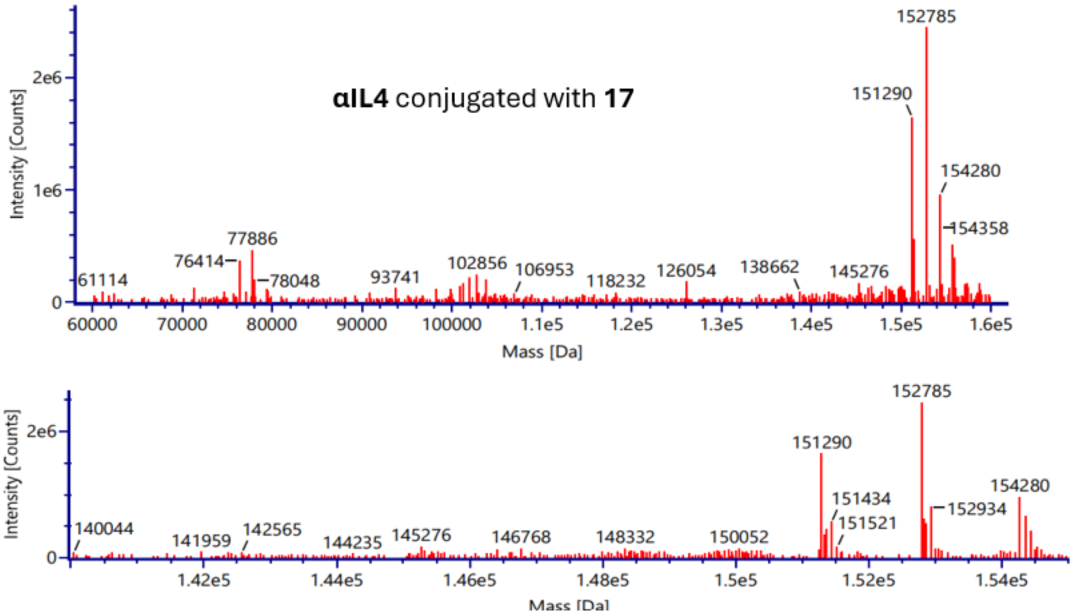

Figure SI-19. MS spectra - **αIL4** conjugated with **17**

b. Convergent Approach

Bioconjugation Step:

A solution of pyridazinedione **3** (20 molar eq, 5 mM in DMSO) was added to **αIL4** (3 mg/mL, 20 µM, in pH 8.5 BBS) and the reaction was incubated at 4 °C for 1 h. After this time a solution of TCEP (10 molar eq, 10 mM in pH 8.5 BBS) and the reaction was incubated for a further 20 h.

After this time the reaction was diluted with PBS (minimum 2 fold dilution) and purified by ultra-filtration (30 kDa MW cut-off PES membrane, 500 µL, 4 x 5 min @ 12000 rcf, 20 °C). The filtrand was diluted to the nearest graduation, the value was recorded to give yield, and transferred to an Eppendorf vial. The samples were analysed by UV-Vis to determine the protein concentration, as well as mass spectrometry and SDS-PAGE.

The payload attachment and Click chemistry steps were carried out using the general procedures described in the Methods section of the manuscript.

Payload	aDAR	DAR range
None (pre-click)	4.24	3 (25.4), 4 (45.1), 5 (29.4), 6 (11.5)
Biotin (**11**)	4.21	3 (32.3), 4 (42.8), 5 (24.9), 6 (15.9)

Table SI-9. aDAR values and DAR ranges for the ADCs produced via the modular and convergent approach

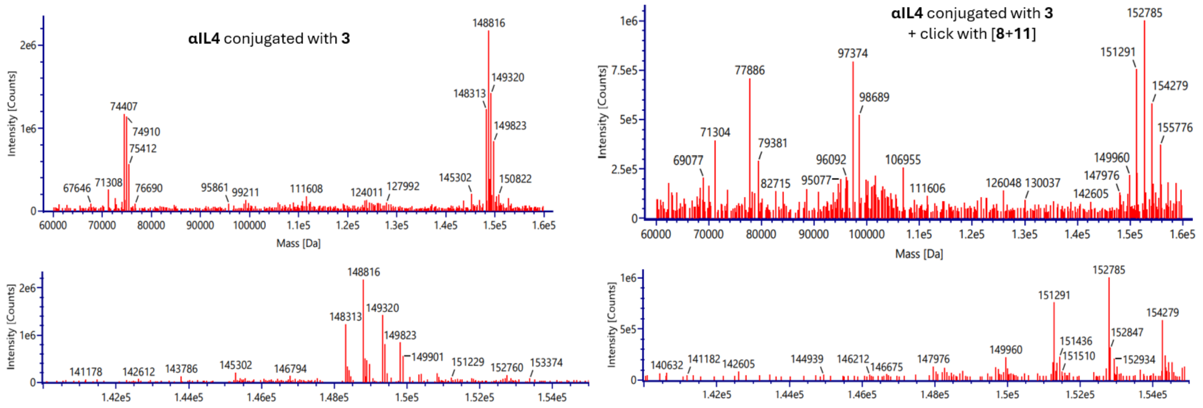

Figure SI-20. MS spectra - LEFT= **αIL4** conjugated with **3** (Left), RIGHT = **αIL4** conjugated with **3** + click reaction with [**8**+**11**]

c. SDS-PAGE – convergent vs linear approach

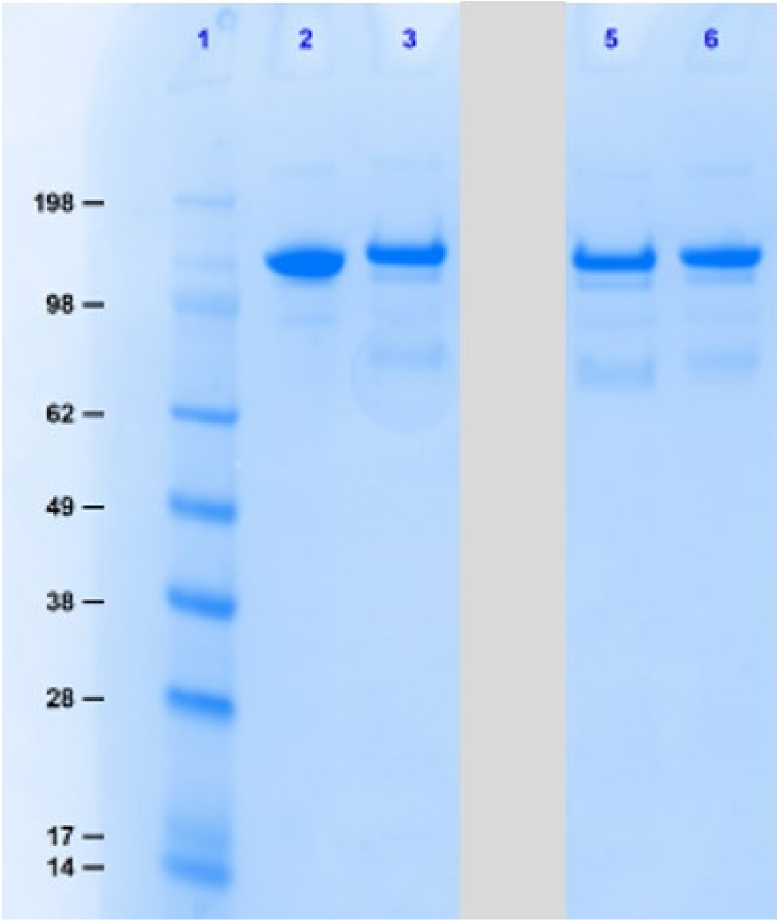

Figure SI-21: 1 = MW Ladder; 2 = unmodified **αIL4**; 3 = αIL4 conjugated with **17** , 5: **αIL4** conjugated with **3** only, 6: **αIL4** conjugated with **3** followed by click with [**8**+**11**]

11.HNMR and LCMS data for key compounds 1-10

N-(2-(2-(2-(2-azidoethoxy)ethoxy)ethoxy)ethyl)-3-phenyl-1,2-oxaziridine-2-carboxamide **1**

LCMS (HPH)

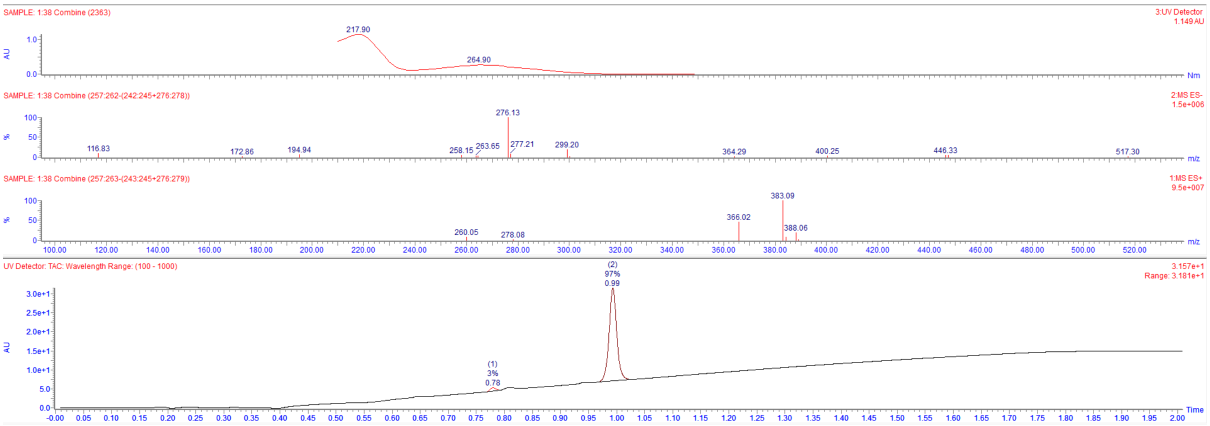

HNMR (CDCl3)

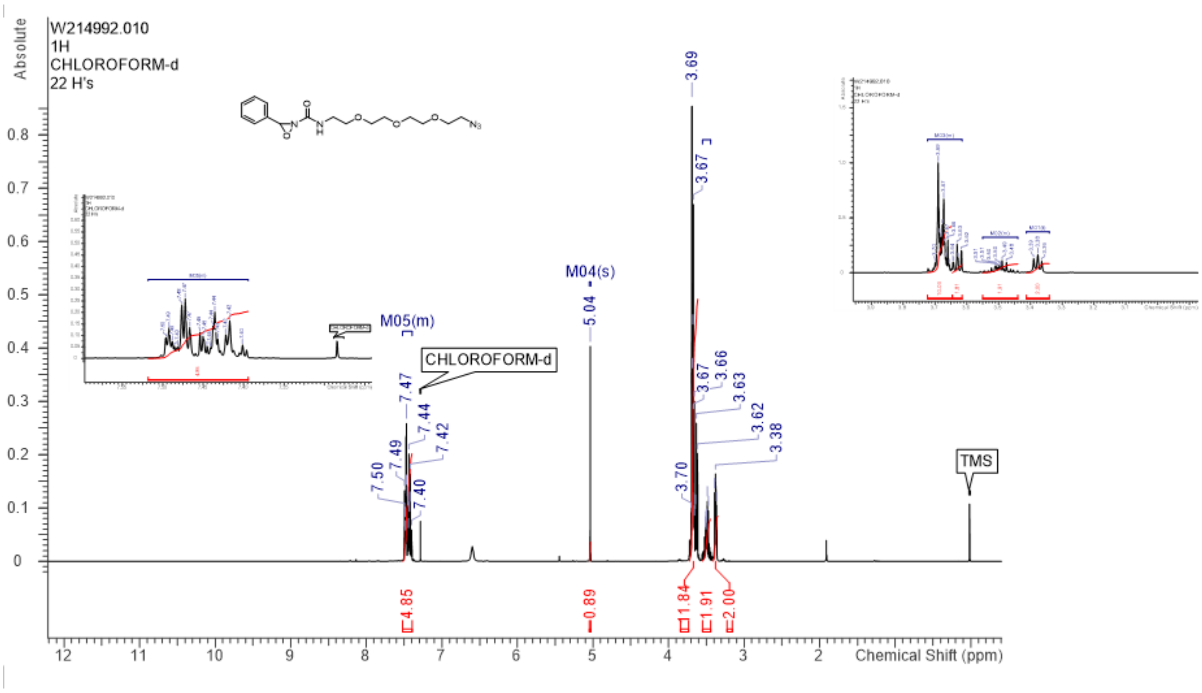

4-(2-(2-(2-(2-Azidoethoxy)ethoxy)ethoxy)ethyl)-1,2,4-triazolidine-3,5-dione **pre-2**

HNMR (DMSO-d6)

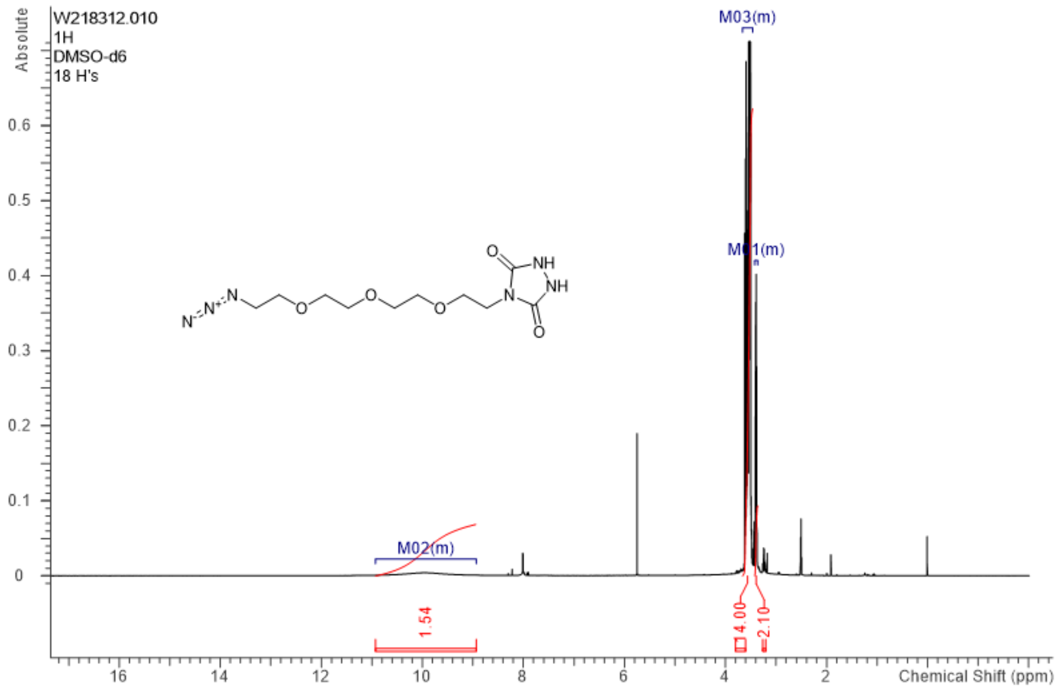

4-(2-(2-(2-(2-azidoethoxy)ethoxy)ethoxy)ethyl)-3H-1,2,4-triazole-3,5(4H)-dione **2** (generated as solution in d3-MeCN)

HNMR (d3-MeCN)

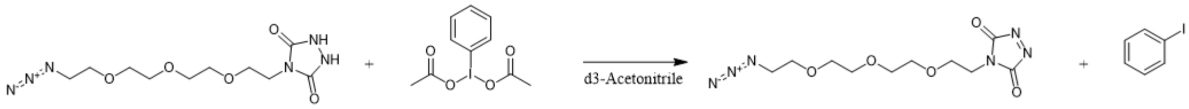

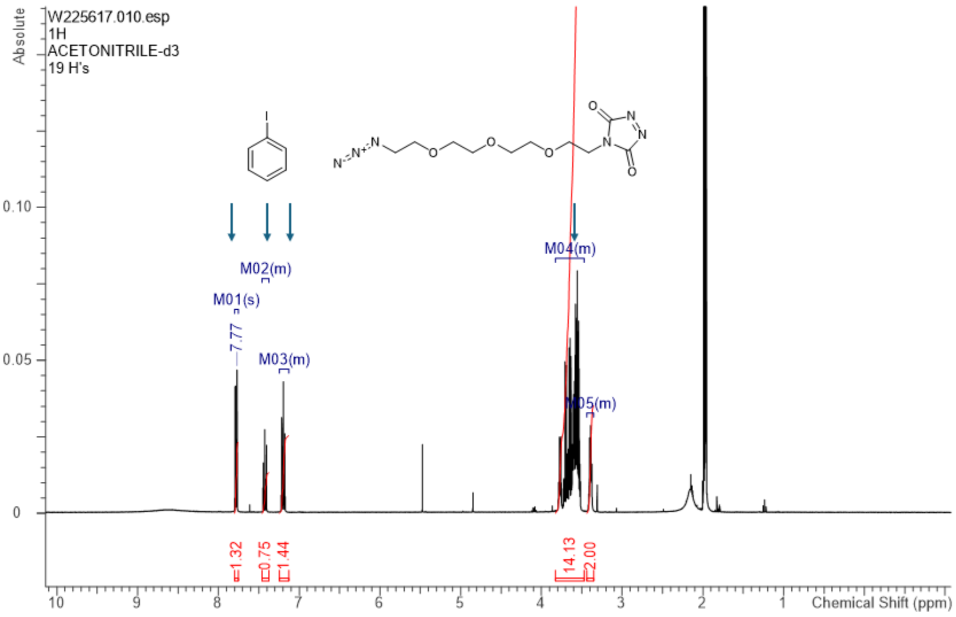

rac-((1R,8S,9s)-Bicyclo[6.1.0]non-4-yn-9-yl)methyl (2-(2-(2-(3-(4,5-dibromo-2-methyl-3,6-dioxo-3,6-dihydropyridazin-1(2H)-yl)propanamido)ethoxy)ethoxy)ethyl)carbamate **3**

LCMS (FOR)

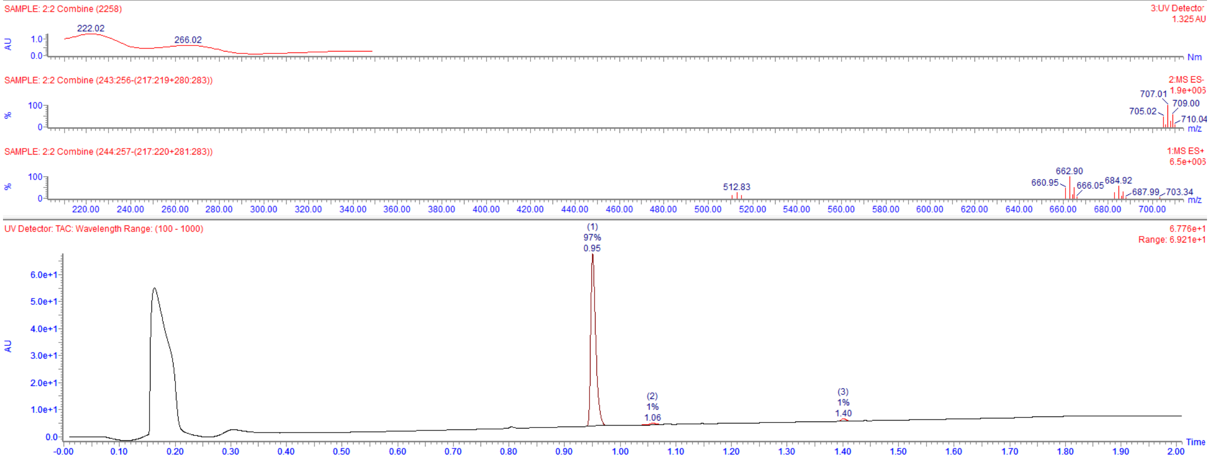

HNMR (DMSO-d6)

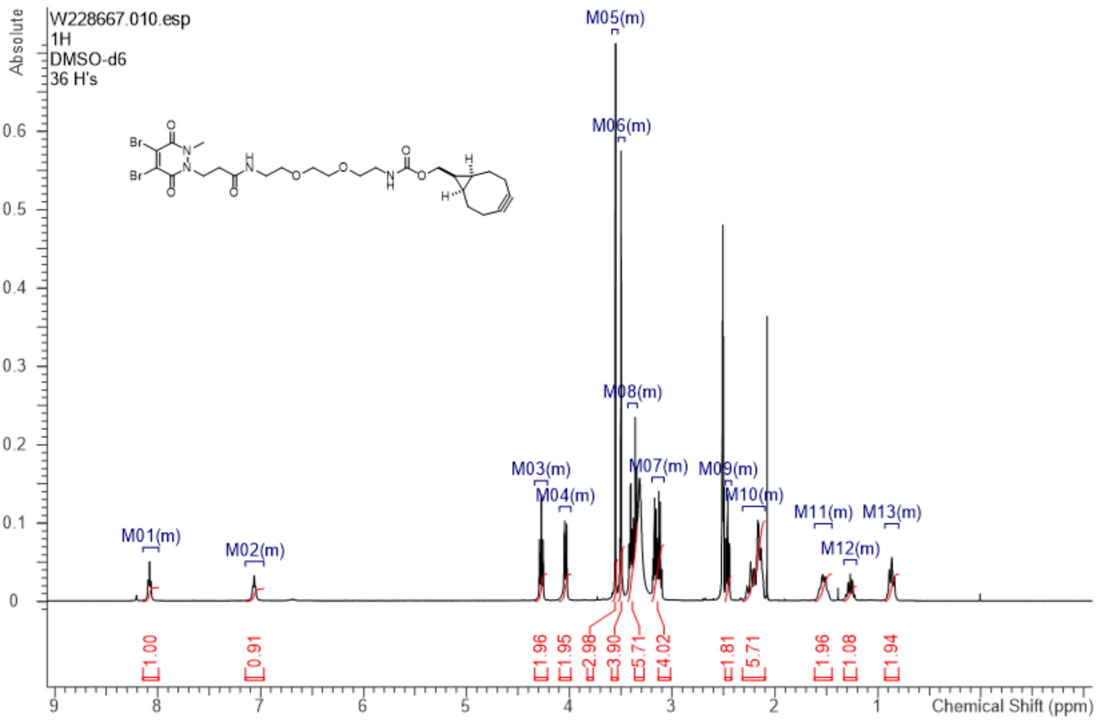

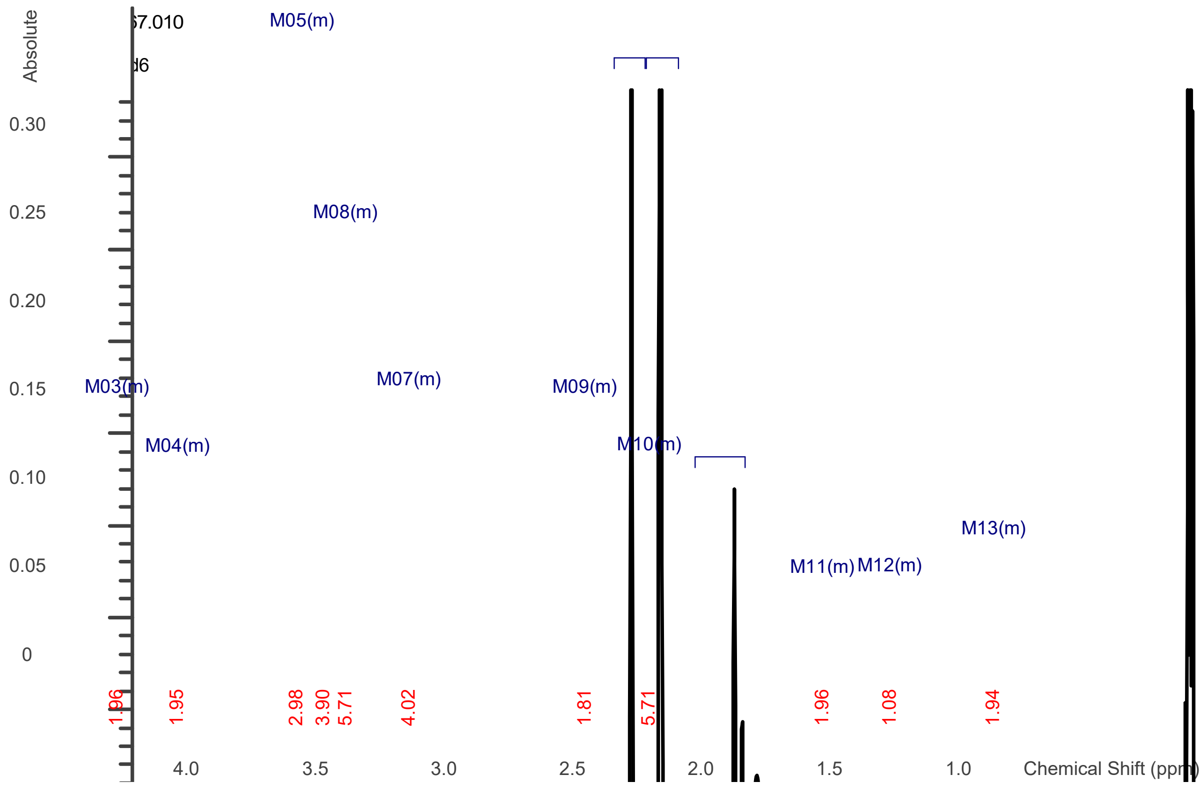

(rac-(1R,8S,9s)-bicyclo[6.1.0]non-4-yn-9-yl)methyl ((S)-3-methyl-1-(((S)-1-((4-((((4-nitrophenoxy)carbonyl)oxy)methyl)phenyl)amino)-1-oxo-5-ureidopentan-2-yl)amino)-1-oxobutan-2-yl)carbamate **5**

LCMS (FOR)

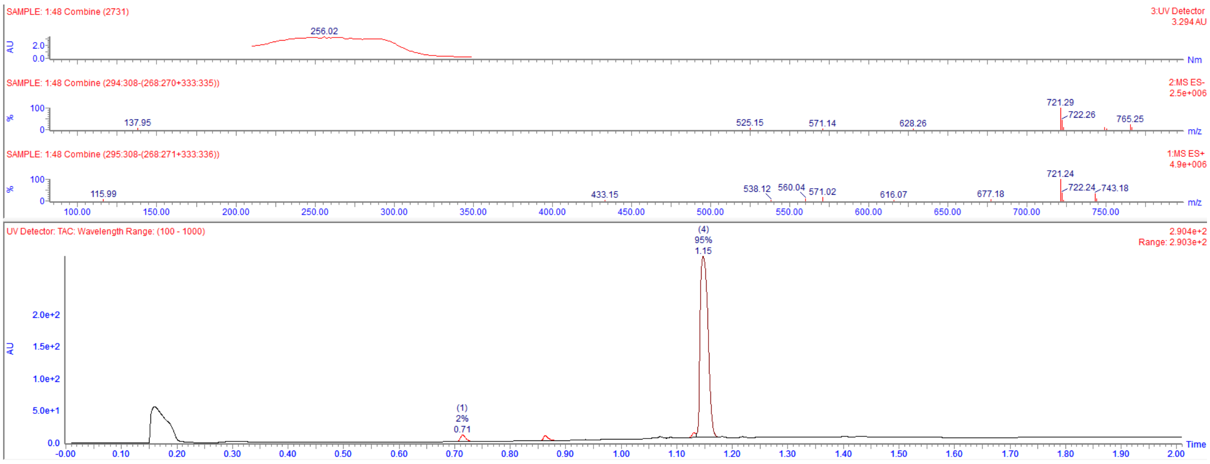

HNMR (DMSO-d6)

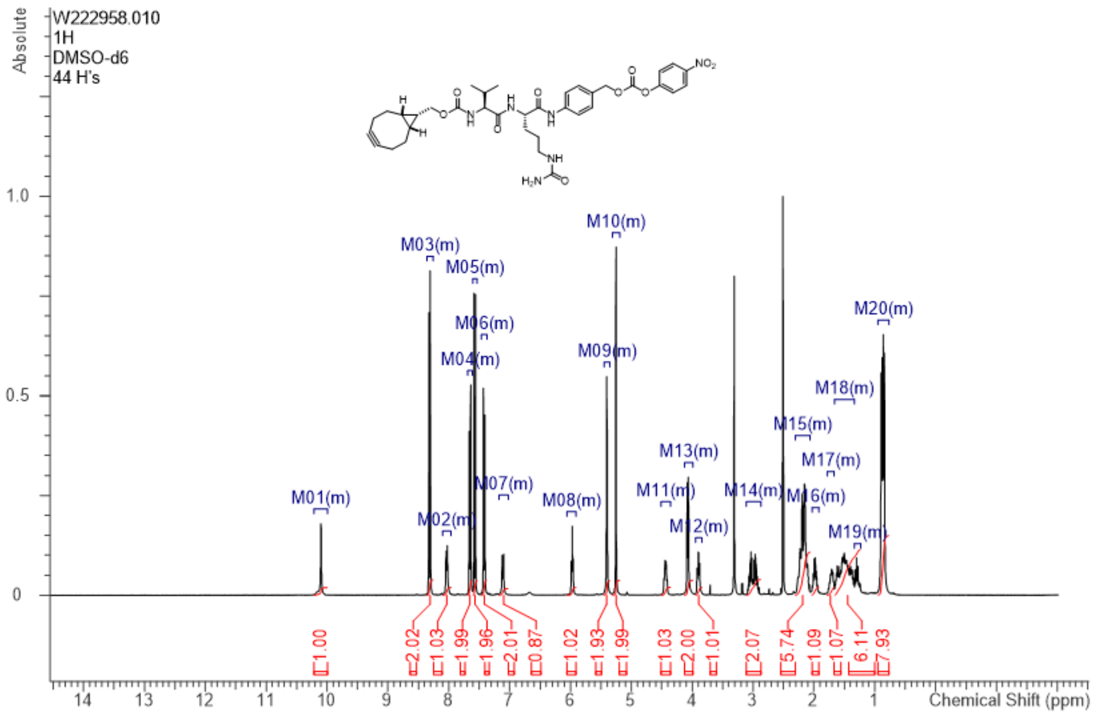
W222958.010
1H
DMSO-d6
44 H's
M01(m)
M02(m)
M03(m)
M04(m)
M05(m)
M06(m)
M07(m)
M08(m)
M09(m)
M10(m)
M11(m)
M12(m)
M13(m)
M14(m)
M15(m)
M16(m)
M17(m)
M18(m)
M19(m)
M20(m)
1.00
2.02
1.03
1.99
1.96
2.01
0.87
1.02
1.93
1.99
1.03
2.00
1.01
2.07
5.74
1.09
1.07
6.11
7.93
Chemical Shift (ppm)
Absolute

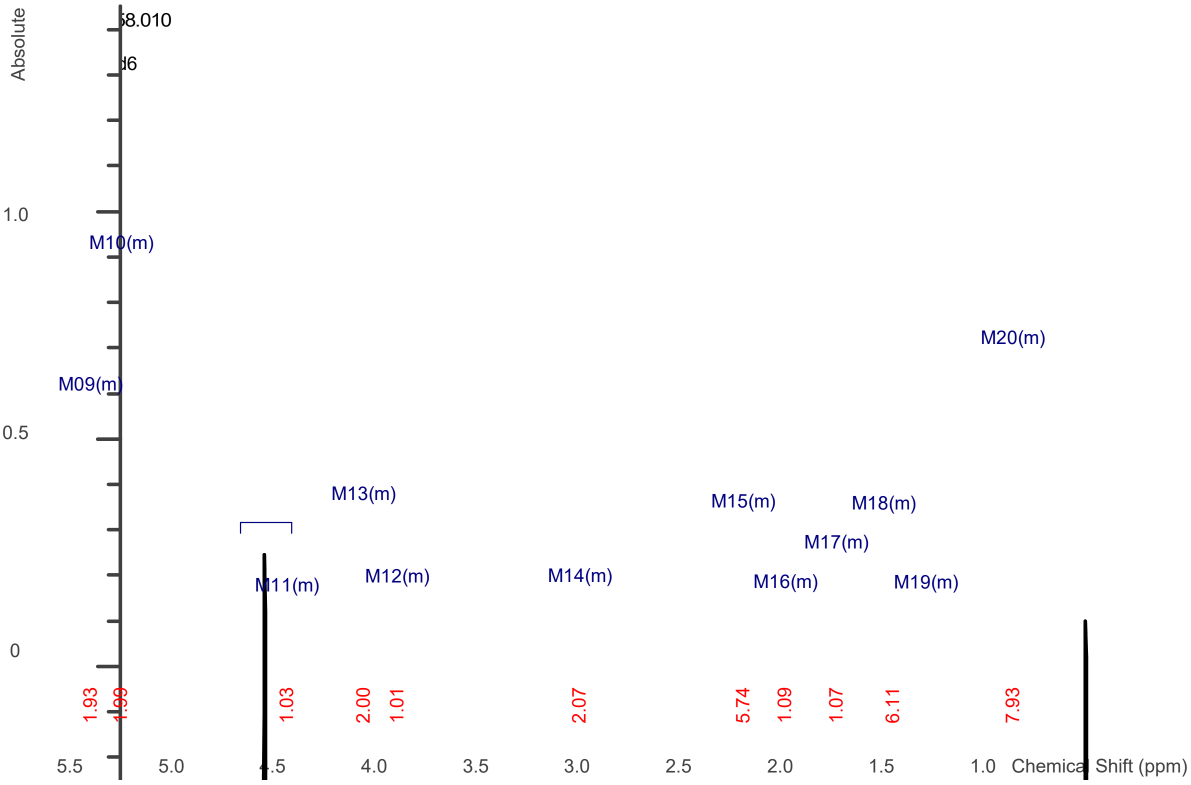
Absolute
M10(m)
M09(m)
M13(m)
M11(m)
M12(m)
M14(m)
M15(m)
M16(m)
M17(m)
M18(m)
M19(m)
M20(m)
1.93
1.99
1.03
2.00
1.01
2.07
5.74
1.09
1.07
6.11
7.93
Chemical Shift (ppm)

(rac-(1R,8S,9s)-Bicyclo[6.1.0]non-4-yn-9-yl)methyl ((S)-3-methyl-1-(((S)-1-((4-((((4-nitrophenoxy)carbonyl)oxy)methyl)phenyl)amino)-1-oxopropan-2-yl)amino)-1-oxobutan-2-yl)carbamate **6**

LCMS (For)

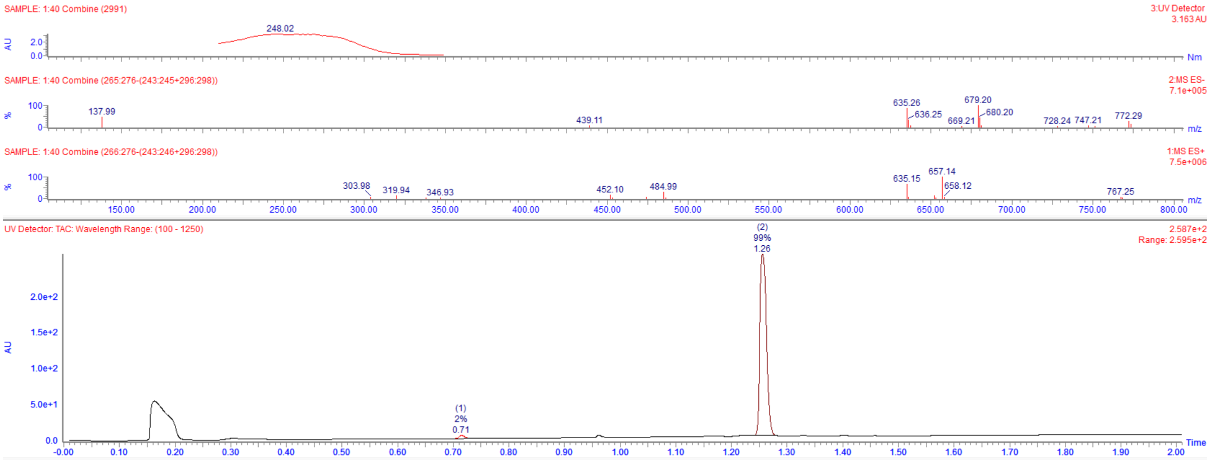

HNMR (DMSO-d6)

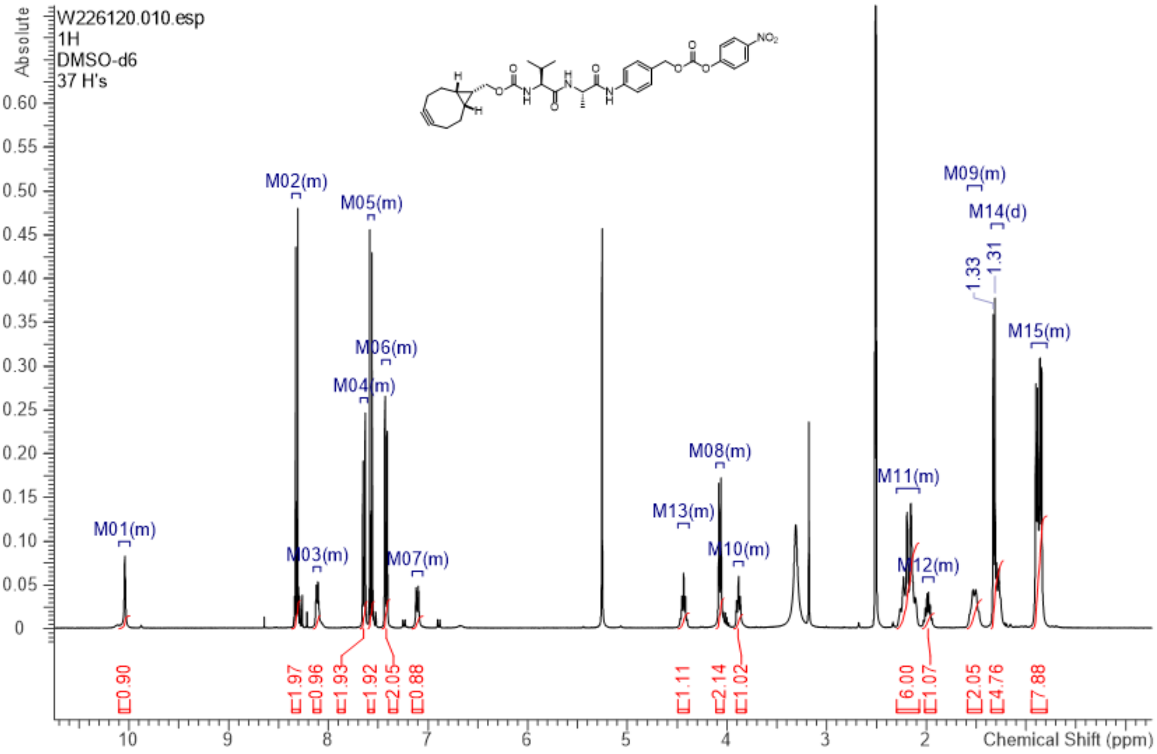

(rac-(1R,8S,9s)-Bicyclo[6.1.0]non-4-yn-9-yl)methyl (20-(4-((((4-nitrophenoxy)carbonyl)-oxy)methyl)phenoxy)-10-oxo-3,6,12,15,18-pentaoxa-9-azaicosyl)carbamate **7**

LCMS (FOR)

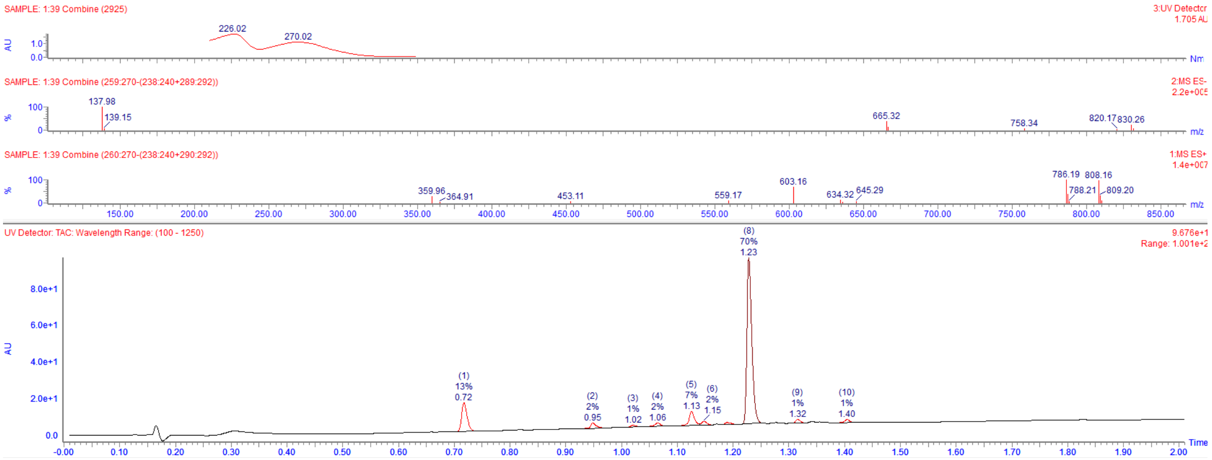

HNMR (DMSO-d6)

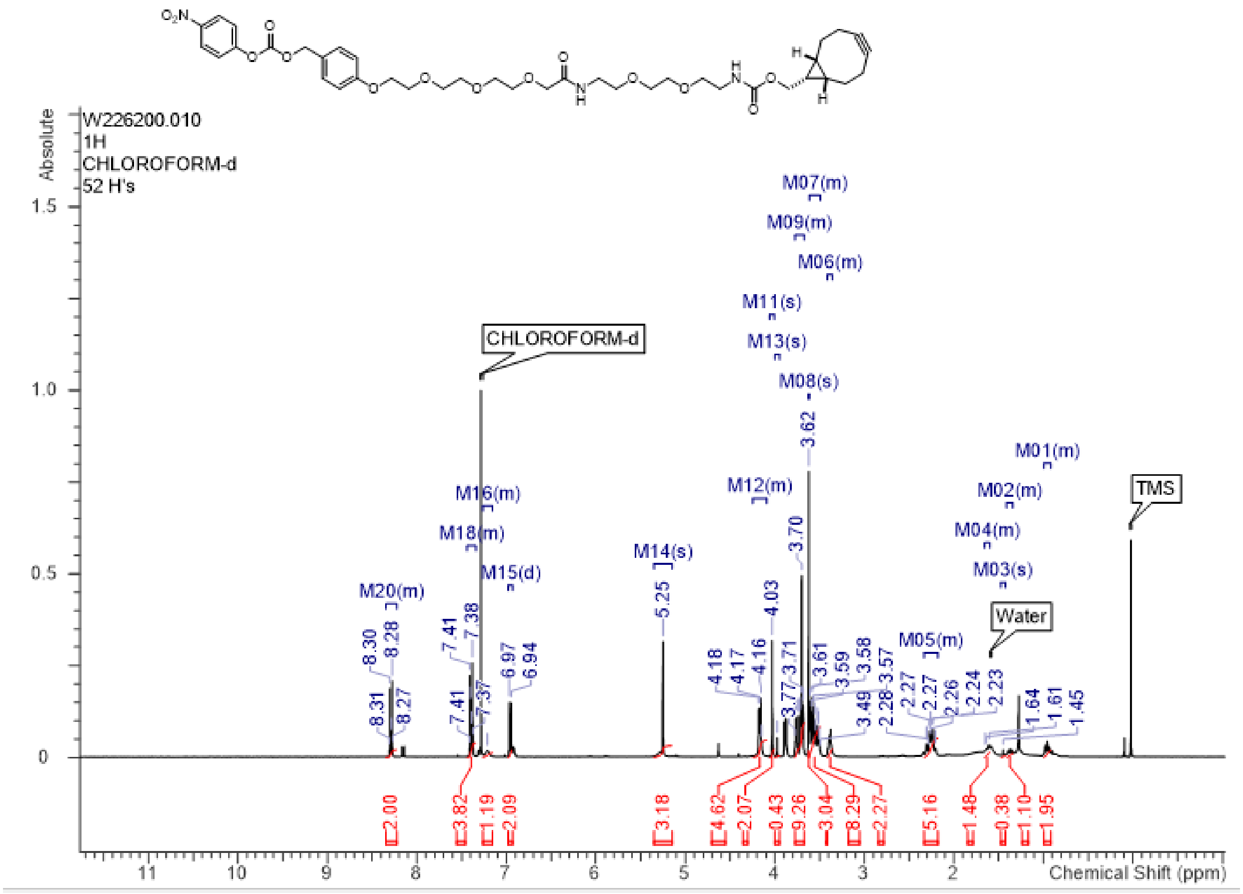

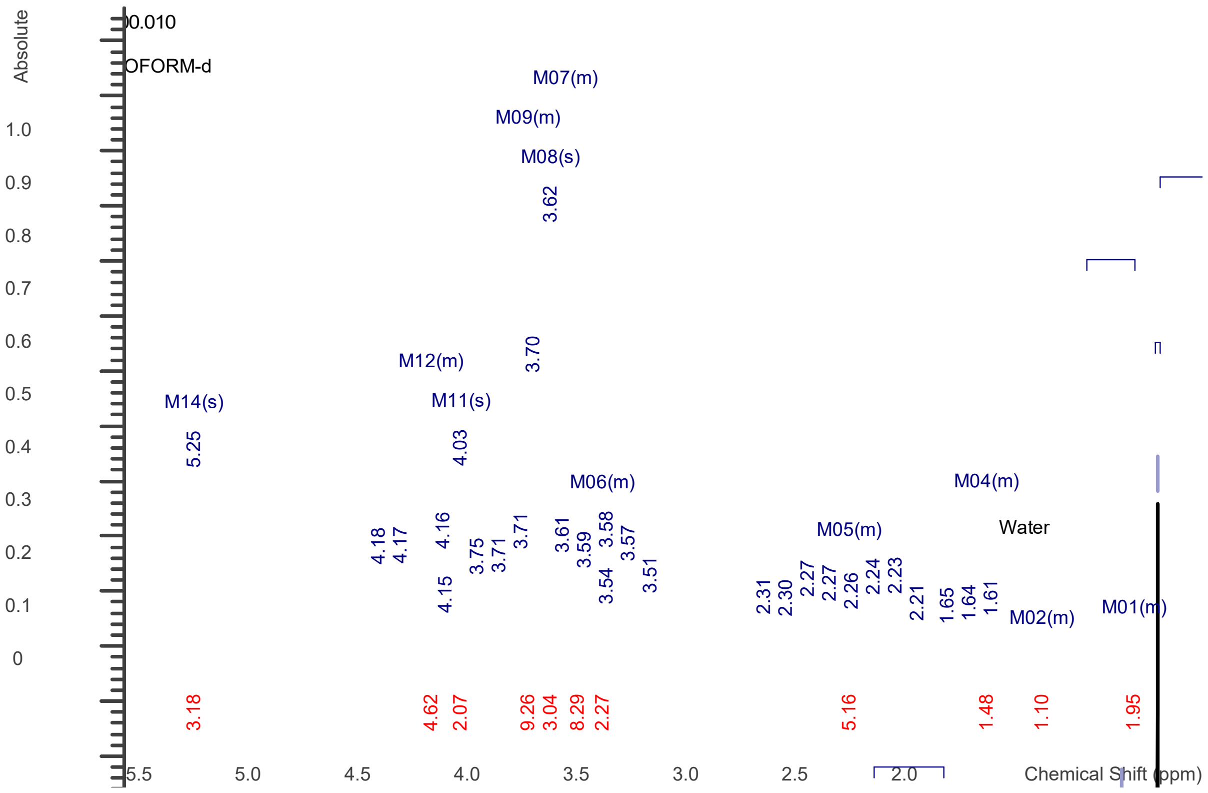

4-((18S,21S)-1-Azido-18-isopropyl-13,16,19-trioxo-21-(3-ureidopropyl)-3,6,9-trioxa-2,17,20-triazadocosan-22-amido)benzyl (4-nitrophenyl) carbonate **8**

LCMS (HPH)

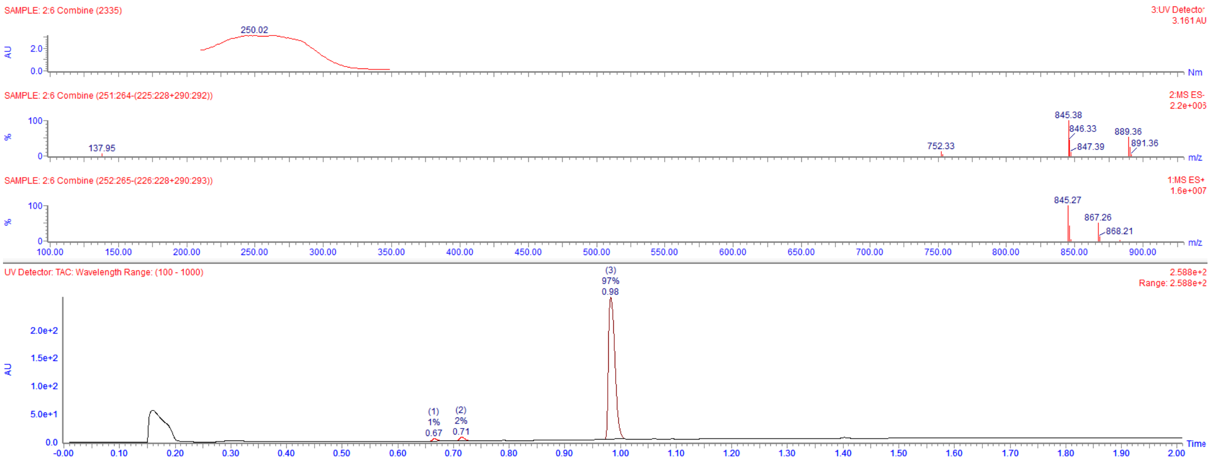

HNMR (DMSO-d6)

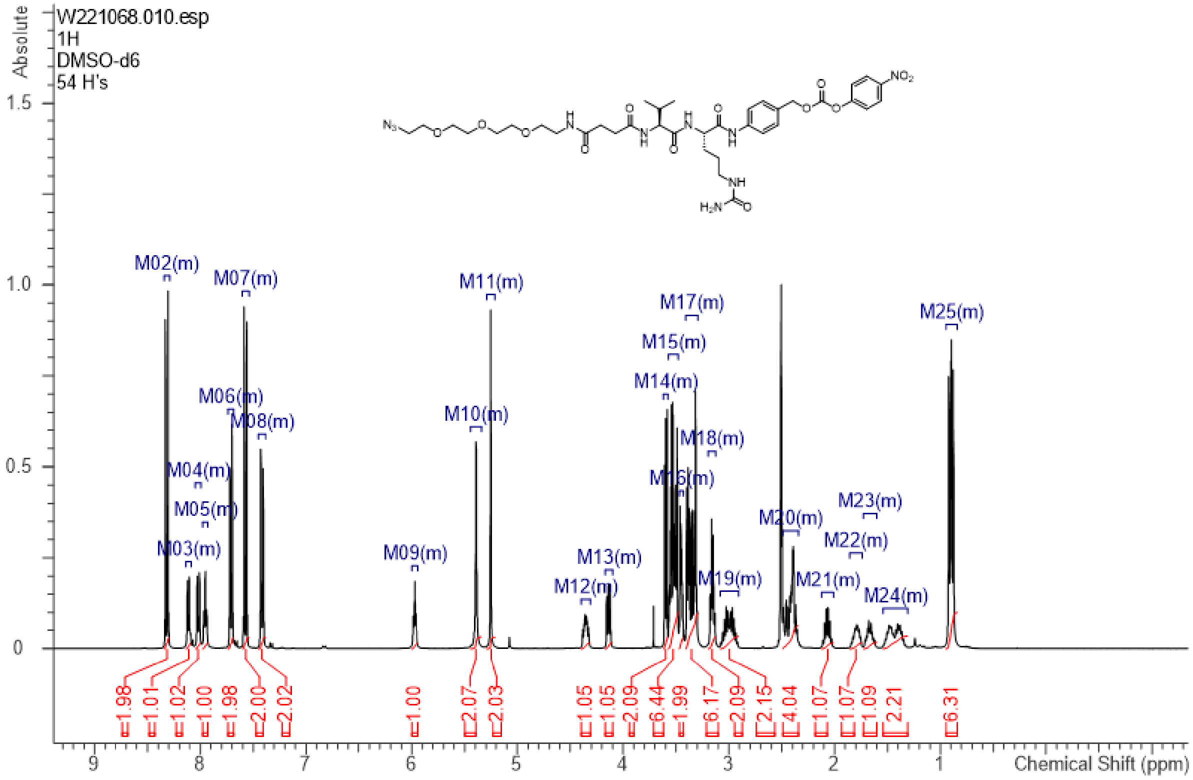
W221068.010.esp
1H
DMSO-d6
54 H's
Absolute
M02(m)
M03(m)
M04(m)
M05(m)
M06(m)
M07(m)
M08(m)
M09(m)
M10(m)
M11(m)
M12(m)
M13(m)
M14(m)
M15(m)
M16(m)
M17(m)
M18(m)
M19(m)
M20(m)
M21(m)
M22(m)
M23(m)
M24(m)
M25(m)
1.98
1.01
1.02
1.00
1.98
2.00
2.02
1.00
2.07
2.03
1.05
1.05
2.09
6.44
1.99
6.17
2.09
2.15
4.04
1.07
1.07
1.09
2.21
6.31
Chemical Shift (ppm)

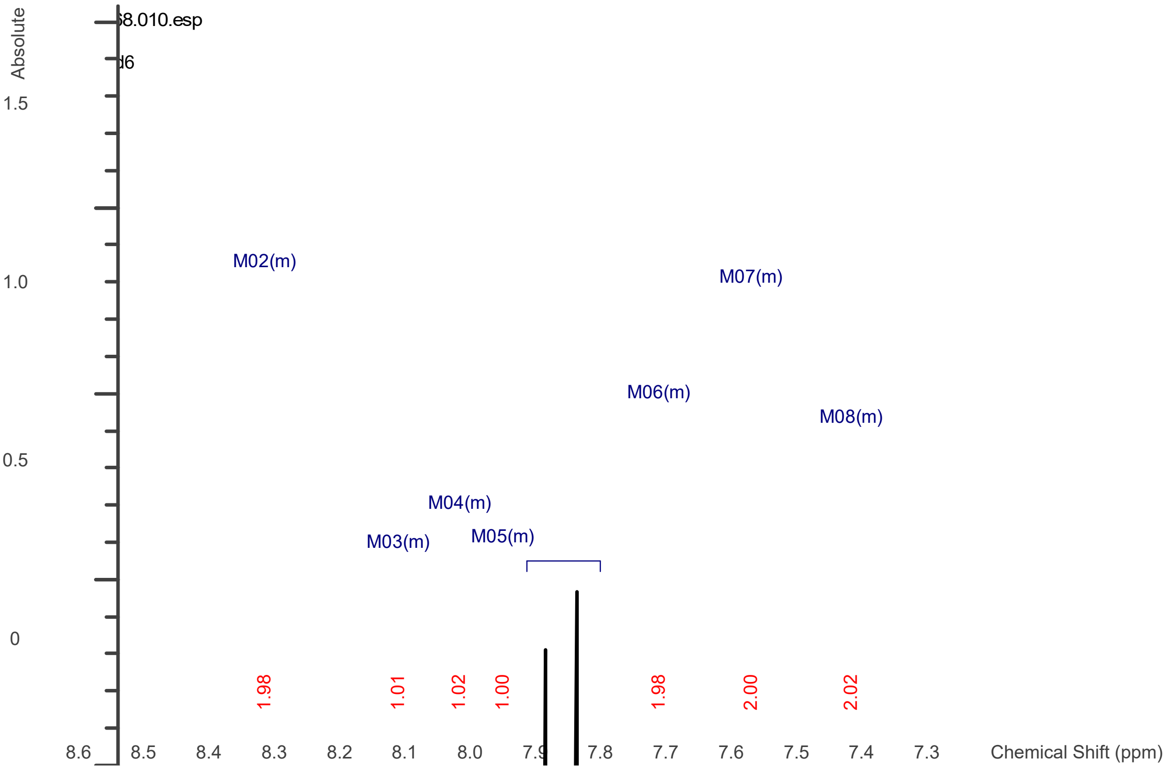
8.010.esp
d6
Absolute
M02(m)
M07(m)
M06(m)
M08(m)
M04(m)
M03(m)
M05(m)
1.98
1.01
1.02
1.00
1.98
2.00
2.02
Chemical Shift (ppm)

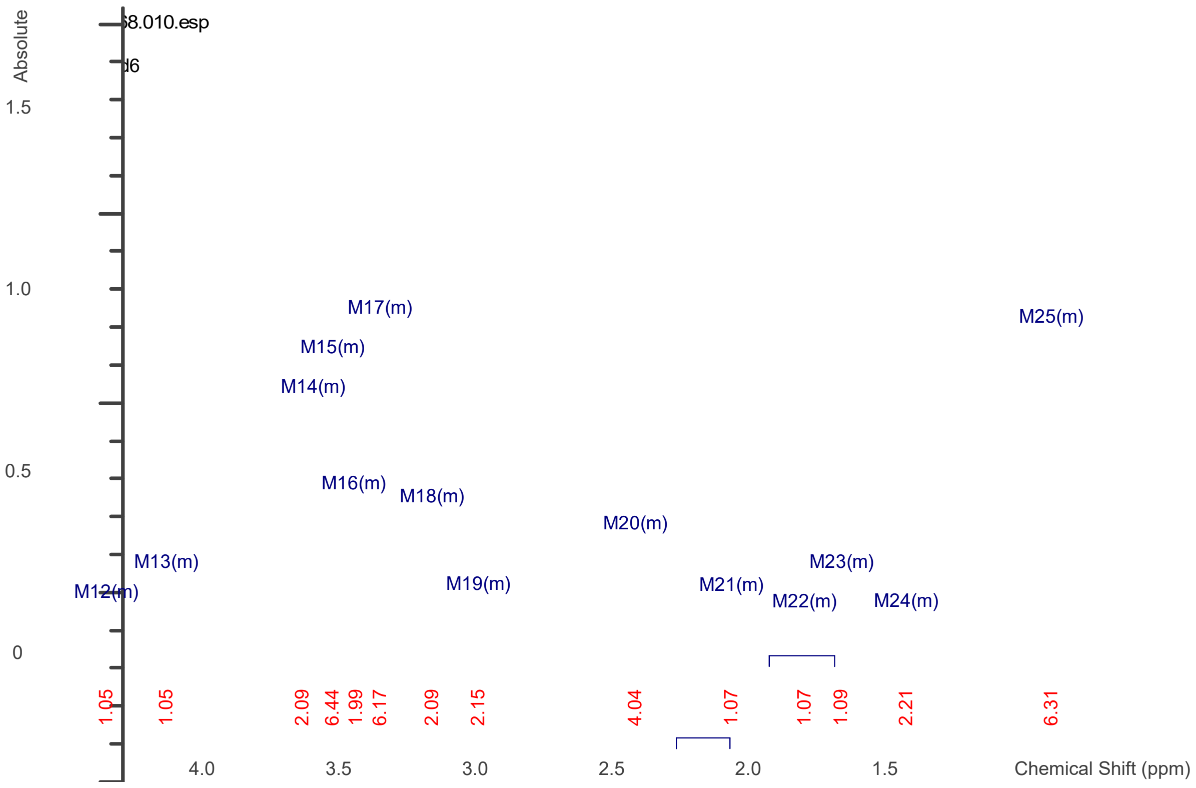

4-((18S,21S)-1-Azido-18-isopropyl-21-methyl-13,16,19-trioxo-3,6,9-trioxa-12,17,20-triazadocosan-22-amido)benzyl (4-nitrophenyl) carbonate **9**

LCMS (HPH)

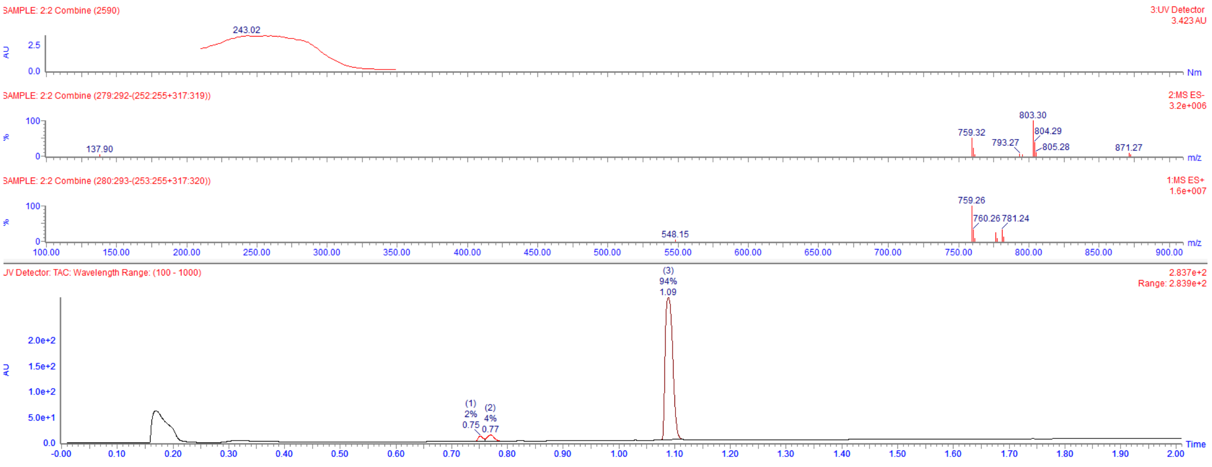

HNMR (DMSO-d6)

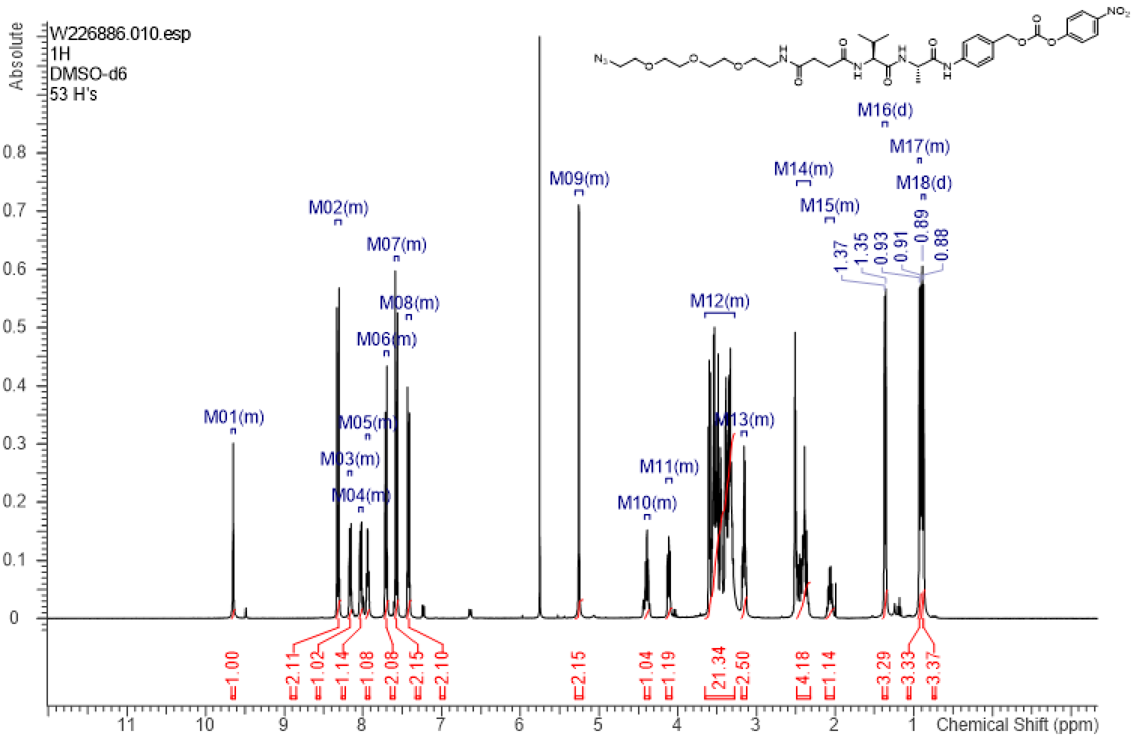

W226886.010.esp
1H
DMSO-d6
53 H's
Absolute
M01(m)
M02(m)
M03(m)
M04(m)
M05(m)
M06(m)
M07(m)
M08(m)
M09(m)
M10(m)
M11(m)
M12(m)
M13(m)
M14(m)
M15(m)
M16(d)
M17(m)
M18(d)
1.00
2.11
1.02
1.14
1.08
2.08
2.15
2.10
2.15
1.04
1.19
21.34
2.50
4.18
1.14
3.29
3.33
3.37
Chemical Shift (ppm)

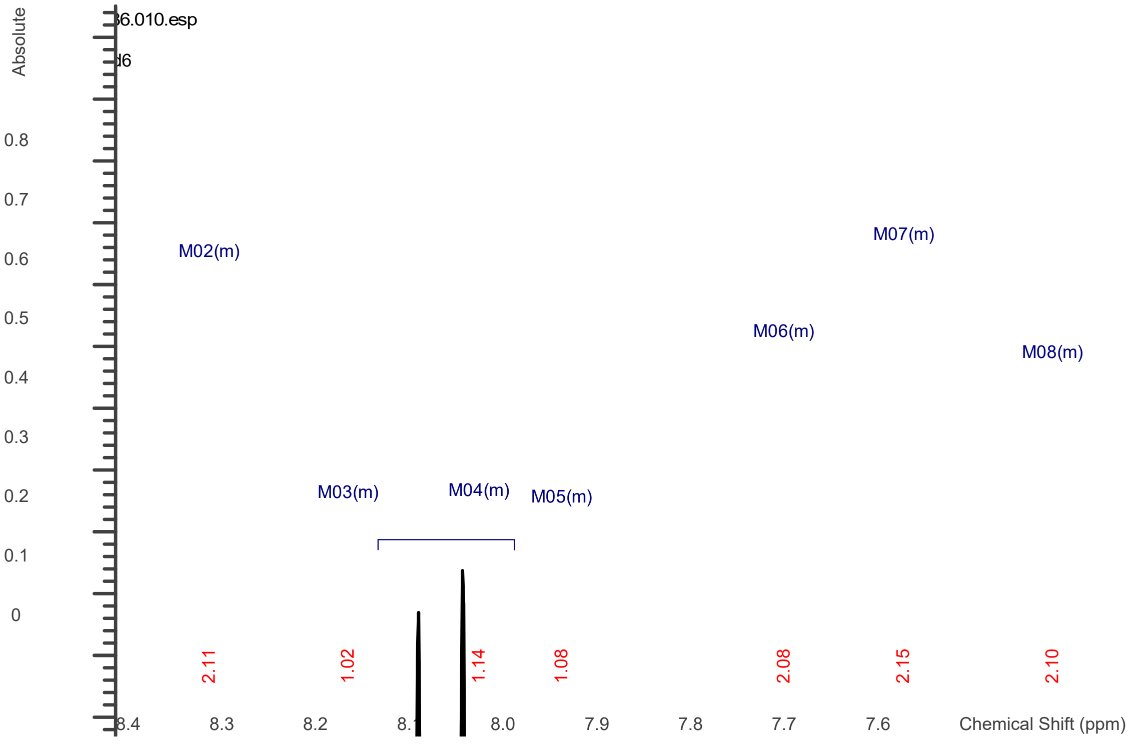

36.010.esp
d6
Absolute
M02(m)
M07(m)
M06(m)
M08(m)
M03(m)
M04(m)
M05(m)
2.11
1.02
1.14
1.08
2.08
2.15
2.10
Chemical Shift (ppm)

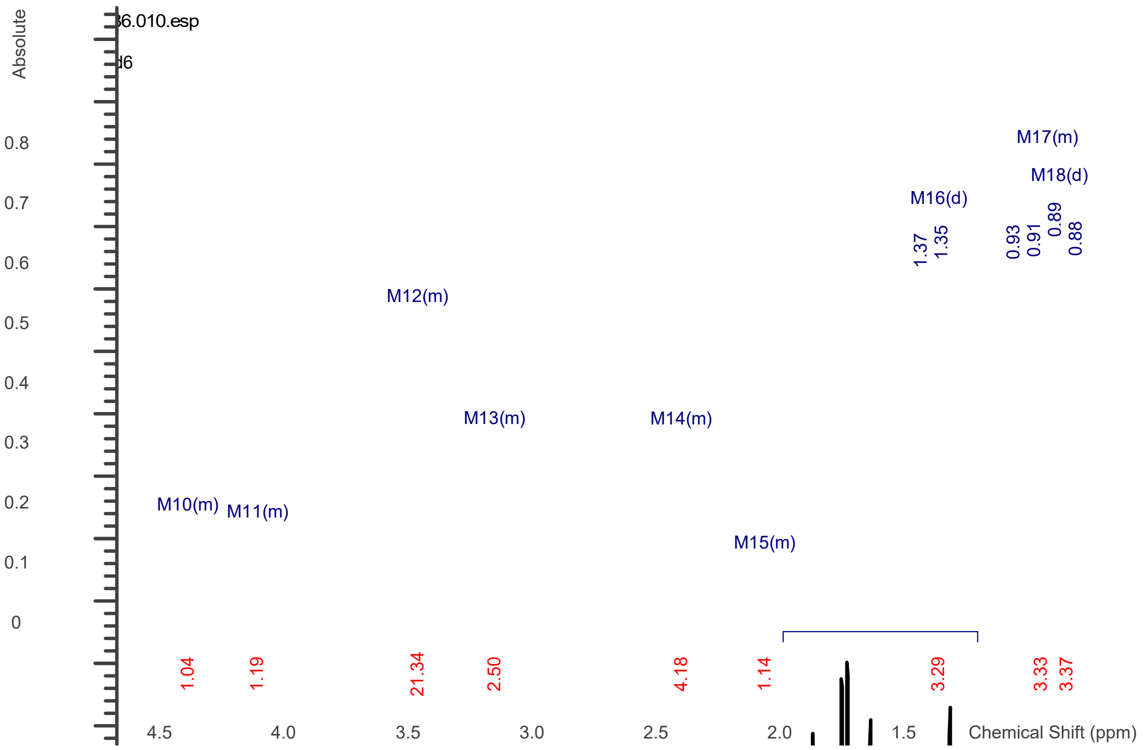

4-((23-Azido-11-oxo-3,6,9,15,18,21-hexaoxa-12-azatricosyl)oxy)benzyl (4-nitrophenyl)-carbonate **10**

LCMS (FOR)

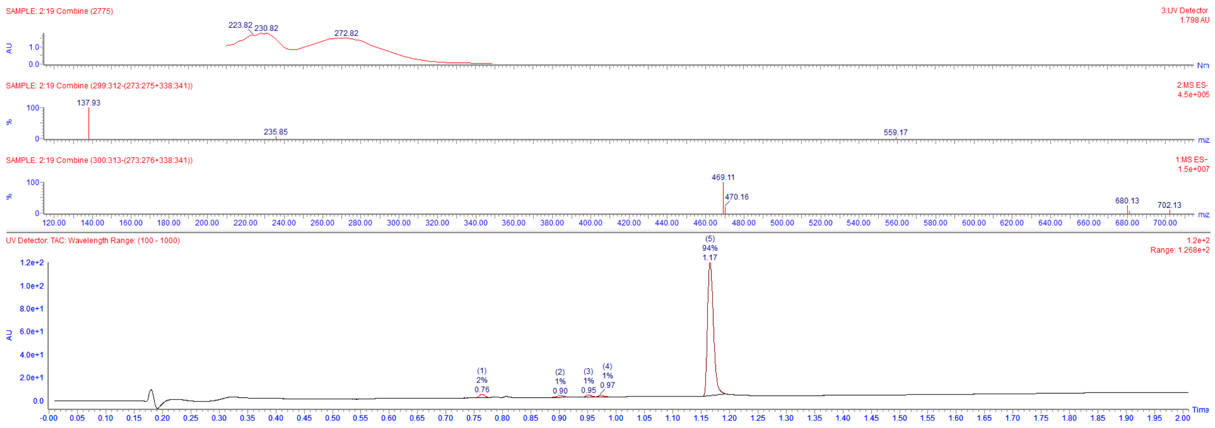

HNMR ($CDCl_3$)

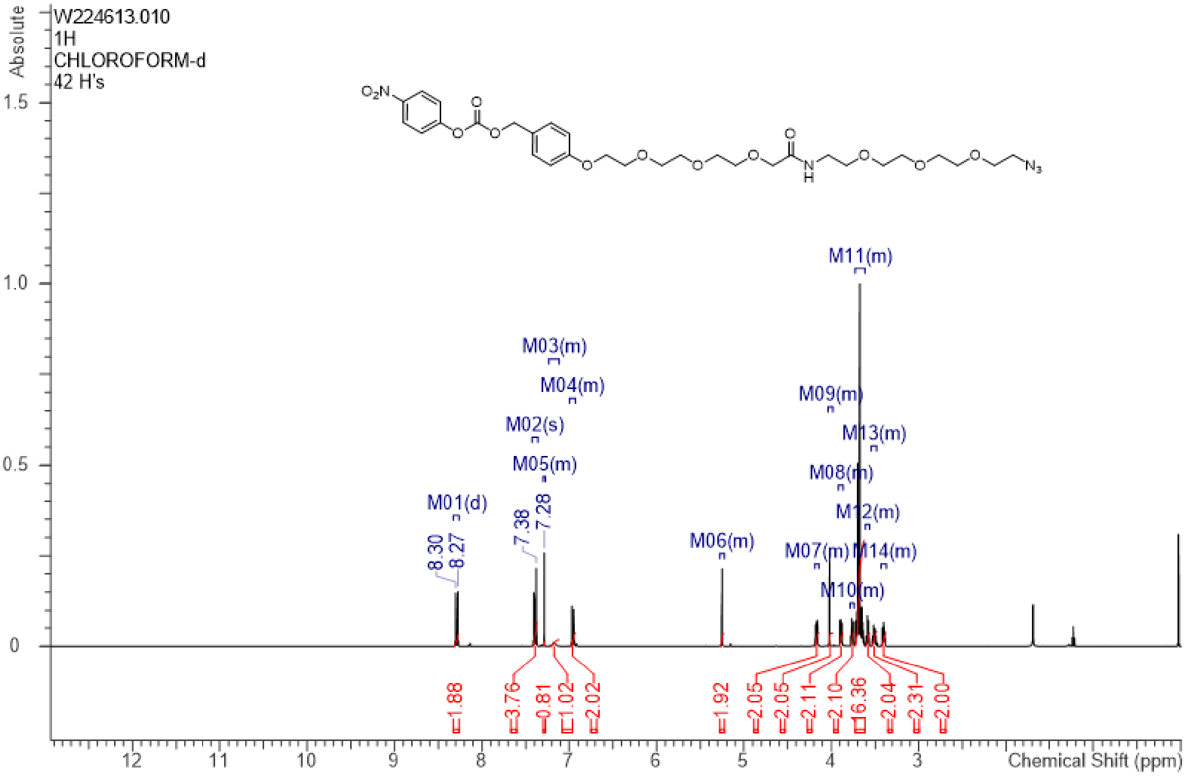
W224613.010
1H
CHLOROFORM-d
42 H's
Absolute
O_2N
N_3
M01(d)
M02(s)
M03(m)
M04(m)
M05(m)
M06(m)
M07(m)
M08(m)
M09(m)
M10(m)
M11(m)
M12(m)
M13(m)
M14(m)
8.30
8.27
7.38
7.28
1.88
3.76
0.81
1.02
2.02
1.92
2.05
2.05
2.11
2.10
16.36
2.04
2.31
2.00
Chemical Shift (ppm)

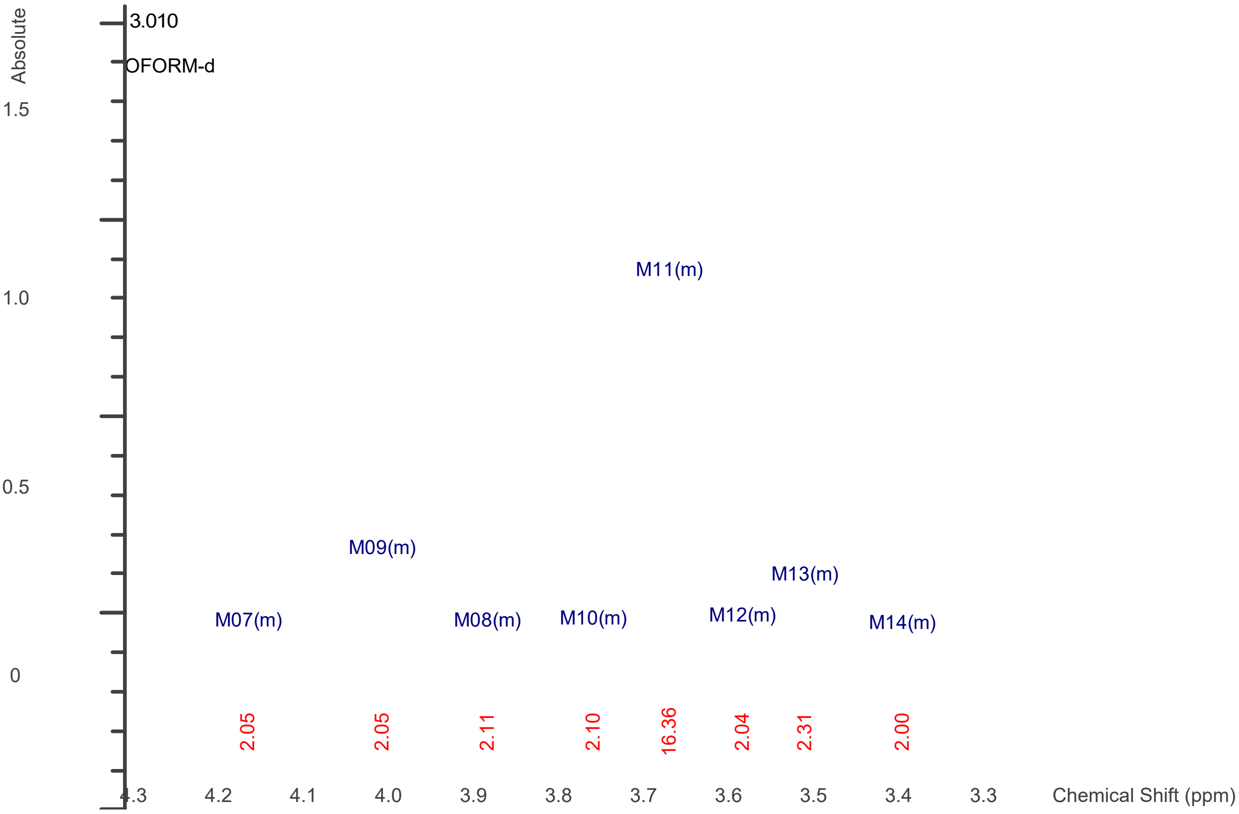
3.010
OFORM-d
Absolute
M07(m)
M09(m)
M08(m)
M10(m)
M11(m)
M12(m)
M13(m)
M14(m)
2.05
2.05
2.11
2.10
16.36
2.04
2.31
2.00
Chemical Shift (ppm)

References:

(1) Zhou, Y.; Wang, G.; Wang, P.; Li, Z.; Yue, T.; Wang, J.; Zou, P., Expanding APEX2 Substrates for Proximity-Dependent Labeling of Nucleic Acids and Proteins in Living Cells. *Angew Chem Int Ed Engl* **2019,** *58* (34), 11763-11767.

(2) Griffiths, R. C.; Smith, F. R.; Long, J. E.; Williams, H. E. L.; Layfield, R.; Mitchell, N. J., Site-Selective Modification of Peptides and Proteins via Interception of Free-Radical-Mediated Dechalcogenation. *Angewandte Chemie International Edition* **2020,** *59* (52), 23659-23667.

(3) Wende, C.; Kulak, N., Fluorophore ATCUN complexes: combining agent and probe for oxidative DNA cleavage. *Chem Commun (Camb)* **2015,** *51* (62), 12395-8.

(4) Mondal, D.; Ford, J.; Pinney, K. G., Improved Methodology for the Synthesis of a Cathepsin B Cleavable Dipeptide Linker, Widely Used in Antibody-Drug Conjugate Research. *Tetrahedron Lett* **2018,** *59* (40), 3594-3599.

(5) Marcher, A.; Nijenhuis, M. A. D.; Gothelf, K. V., A Wireframe DNA Cube: Antibody Conjugate for Targeted Delivery of Multiple Copies of Monomethyl Auristatin E. *Angew Chem Int Ed Engl* **2021,** *60* (40), 21691-21696.

(6) Wei, B.; Gunzner-Toste, J.; Yao, H.; Wang, T.; Wang, J.; Xu, Z.; Chen, J.; Wai, J.; Nonomiya, J.; Tsai, S. P.; Chuh, J.; Kozak, K. R.; Liu, Y.; Yu, S. F.; Lau, J.; Li, G.; Phillips, G. D.; Leipold, D.; Kamath, A.; Su, D.; Xu, K.; Eigenbrot, C.; Steinbacher, S.; Ohri, R.; Raab, H.; Staben, L. R.; Zhao, G.; Flygare, J. A.; Pillow, T. H.; Verma, V.; Masterson, L. A.; Howard, P. W.; Safina, B., Discovery of Peptidomimetic Antibody-Drug Conjugate Linkers with Enhanced Protease Specificity. *J Med Chem* **2018,** *61* (3), 989-1000.

(7) Jensen, M.; Schmidt, S.; Fedosova, N. U.; Mollenhauer, J.; Jensen, H. H., Synthesis and evaluation of cardiac glycoside mimics as potential anticancer drugs. *Bioorg Med Chem* **2011,** *19* (7), 2407-17.

(8) Christian, A. H.; Jia, S.; Cao, W.; Zhang, P.; Meza, A. T.; Sigman, M. S.; Chang, C. J.; Toste, F. D., A Physical Organic Approach to Tuning Reagents for Selective and Stable Methionine Bioconjugation. *J Am Chem Soc* **2019,** *141* (32), 12657-12662.

(9) Elledge, S. K.; Tran, H. L.; Christian, A. H.; Steri, V.; Hann, B.; Toste, F. D.; Chang, C. J.; Wells, J. A., Systematic identification of engineered methionines and oxaziridines for efficient, stable, and site-specific antibody bioconjugation. *Proc. Natl. Acad. Sci.* **2020,** *117* (11), 5733-5740.

(10) Nilo, A.; Allan, M.; Brogioni, B.; Proietti, D.; Cattaneo, V.; Crotti, S.; Sokup, S.; Zhai, H.; Margarit, I.; Berti, F.; Hu, Q. Y.; Adamo, R., Tyrosine-directed conjugation of large glycans to proteins via copper-free click chemistry. *Bioconjug Chem* **2014,** *25* (12), 2105-11.

(11) Ban, H.; Gavrilyuk, J.; Barbas, C. F., 3rd, Tyrosine bioconjugation through aqueous ene-type reactions: a click-like reaction for tyrosine. *J Am Chem Soc* **2010,** *132* (5), 1523-5.

(12) Sato, S.; Nakamura, K.; Nakamura, H., Tyrosine-Specific Chemical Modification with in Situ Hemin-Activated Luminol Derivatives. *ACS Chem Biol* **2015,** *10* (11), 2633-40.

(13) Bahou, C.; Richards, D. A.; Maruani, A.; Love, E. A.; Javaid, F.; Caddick, S.; Baker, J. R.; Chudasama, V., Highly homogeneous antibody modification through optimisation of the synthesis and conjugation of functionalised dibromopyridazinediones. *Org. Biomol. Chem.* **2018,** *16* (8), 1359-1366.

(14) Maruani, A.; Savoie, H.; Bryden, F.; Caddick, S.; Boyle, R.; Chudasama, V., Site-selective multi-porphyrin attachment enables the formation of a next-generation antibody-based photodynamic therapeutic. *Chem Commun (Camb)* **2015,** *51* (83), 15304-7.

(15) Macabuag, N.; Esmieu, W.; Breccia, P.; Jarvis, R.; Blackaby, W.; Lazari, O.; Urbonas, L.; Eznarriaga, M.; Williams, R.; Strijbosch, A.; Van de Bospoort, R.; Matthews, K.; Clissold, C.; Ladduwahetty, T.; Vater, H.; Heaphy, P.; Stafford, D. G.; Wang, H. J.; Mangette, J. E.; McAllister, G.; Beaumont, V.; Vogt, T. F.; Wilkinson, H. A.; Doherty, E. M.; Dominguez, C., Developing HDAC4-Selective Protein Degraders To Investigate the Role of HDAC4 in Huntington's Disease Pathology. *J Med Chem* **2022,** *65* (18), 12445-12459.

(16) Munoz, E.; Chen, G.; Hossain, A.; Wu, S.; Oceguera Nava, E.; Hang, J.; Lee, T.; Zhang, Q.; Wang, G.; Chen, Q. H., Synthesis and biological evaluation of niclosamide PROTACs. *Bioorg Med Chem Lett* **2022,** *72*, 128870.
(17) Clave, G.; Boutal, H.; Hoang, A.; Perraut, F.; Volland, H.; Renard, P. Y.; Romieu, A., A novel heterotrifunctional peptide-based cross-linking reagent for facile access to bioconjugates. Applications to peptide fluorescent labelling and immobilisation. *Org Biomol Chem* **2008,** *6* (17), 3065-78.
(18) Wang, J.; Li, H.; Zou, G.; Wang, L. X., Novel template-assembled oligosaccharide clusters as epitope mimics for HIV-neutralizing antibody 2G12. Design, synthesis, and antibody binding study. *Org Biomol Chem* **2007,** *5* (10), 1529-40.
